# Biology of *Acinetobacter baumannii*: Pathogenesis, Antibiotic Resistance Mechanisms, and Prospective Treatment Options

**DOI:** 10.3389/fcimb.2017.00055

**Published:** 2017-03-13

**Authors:** Chang-Ro Lee, Jung Hun Lee, Moonhee Park, Kwang Seung Park, Il Kwon Bae, Young Bae Kim, Chang-Jun Cha, Byeong Chul Jeong, Sang Hee Lee

**Affiliations:** ^1^National Leading Research Laboratory of Drug Resistance Proteomics, Department of Biological Sciences, Myongji UniversityYongin, South Korea; ^2^DNA Analysis Division, Seoul Institute, National Forensic ServiceSeoul, South Korea; ^3^Department of Dental Hygiene, College of Health and Welfare, Silla UniversityBusan, South Korea; ^4^Biotechnology Program, North Shore Community CollegeDanvers, MA, USA; ^5^Department of Systems Biotechnology, College of Biotechnology and Natural Resources, Chung-Ang UniversityAnseong, South Korea

**Keywords:** antimicrobial resistance, *Acinetobacter baumannii*, treatment option, resistance mechanism, virulence factor

## Abstract

*Acinetobacter baumannii* is undoubtedly one of the most successful pathogens responsible for hospital-acquired nosocomial infections in the modern healthcare system. Due to the prevalence of infections and outbreaks caused by multi-drug resistant *A. baumannii*, few antibiotics are effective for treating infections caused by this pathogen. To overcome this problem, knowledge of the pathogenesis and antibiotic resistance mechanisms of *A. baumannii* is important. In this review, we summarize current studies on the virulence factors that contribute to *A. baumannii* pathogenesis, including porins, capsular polysaccharides, lipopolysaccharides, phospholipases, outer membrane vesicles, metal acquisition systems, and protein secretion systems. Mechanisms of antibiotic resistance of this organism, including acquirement of β-lactamases, up-regulation of multidrug efflux pumps, modification of aminoglycosides, permeability defects, and alteration of target sites, are also discussed. Lastly, novel prospective treatment options for infections caused by multi-drug resistant *A. baumannii* are summarized.

## Introduction

*Acinetobacter* spp. are glucose-non-fermentative, non-motile, non-fastidious, catalase-positive, oxidative-negative, aerobic Gram-negative coccobacilli (Lin and Lan, 2014). Due to clusters of closely related species, it is difficult to distinguish *Acinetobacter* taxonomy using phenotypic traits and chemotaxonomic methods. Because antibiotic susceptibility and clinical relevance are significantly different between different genomic species, exact identification of *Acinetobacter* species are required (Bergogne-Berezin and Towner, 1996; Dijkshoorn et al., 1996; Houang et al., 2003; Lee et al., 2007). Many genomic fingerprinting methods have been developed, including repetitive extragenic palindromic sequence-based polymerase chain reaction (rep-PCR), pulsed-field gel electrophoresis (PFGE), matrix-assisted laser desorption ionization time-of-flight (MALDI-TOF) mass spectrometry, ribotyping, amplified ribosomal DNA restriction analysis, random amplified polymorphic DNA analysis, multilocus sequence typing (MLST), RNA spacer fingerprinting, amplified fragment length polymorphism analysis, and sequence analysis of 16S-23S rRNA intergene spacer regions or the *rpoB* and *gyrB* genes (Koeleman et al., 1998; Chang et al., 2005; La Scola et al., 2006; Croxatto et al., 2012; Higgins et al., 2012; Lee C. R. et al., 2015; Li X. M. et al., 2016).

Among *Acinetobacter* species, *Acinetobacter baumannii* is the most important member associated with hospital-acquired infections worldwide (Lin and Lan, 2014). This aerobic Gram-negative coccobacillus had been regarded as a low-grade pathogen, but it is a successful pathogen responsible for opportunistic infections of the skin, bloodstream, urinary tract, and other soft tissues (Peleg et al., 2008). Because many *A. baumannii* infections have suddenly been reported among veterans and soldiers who served in Iraq and Afghanistan (Centers for Disease and Prevention, 2004), *A. baumannii* is referred to as “Iraqibacter.” Multidrug-resistant (MDR) *A. baumannii* has spread to civilian hospitals in part by cross-infection of injured military patients repatriated from war zones (Peleg et al., 2008). Most *A. baumannii* infections occur in critically ill patients in the intensive care unit (ICU) setting (Fournier and Richet, 2006) and account for up to 20% of infections in ICUs worldwide (Vincent et al., 2009). Furthermore, the frequency of community-acquired *A. baumannii* infections has been increasing gradually (Lin and Lan, 2014). Several virulence factors have been identified by genomic and phenotypic analyses, including outer membrane porins, phospholipases, proteases, lipopolysaccharides (LPS), capsular polysaccharides, protein secretion systems, and iron-chelating systems (Antunes et al., 2011; McConnell et al., 2013; Lin and Lan, 2014).

**Figure 1:**
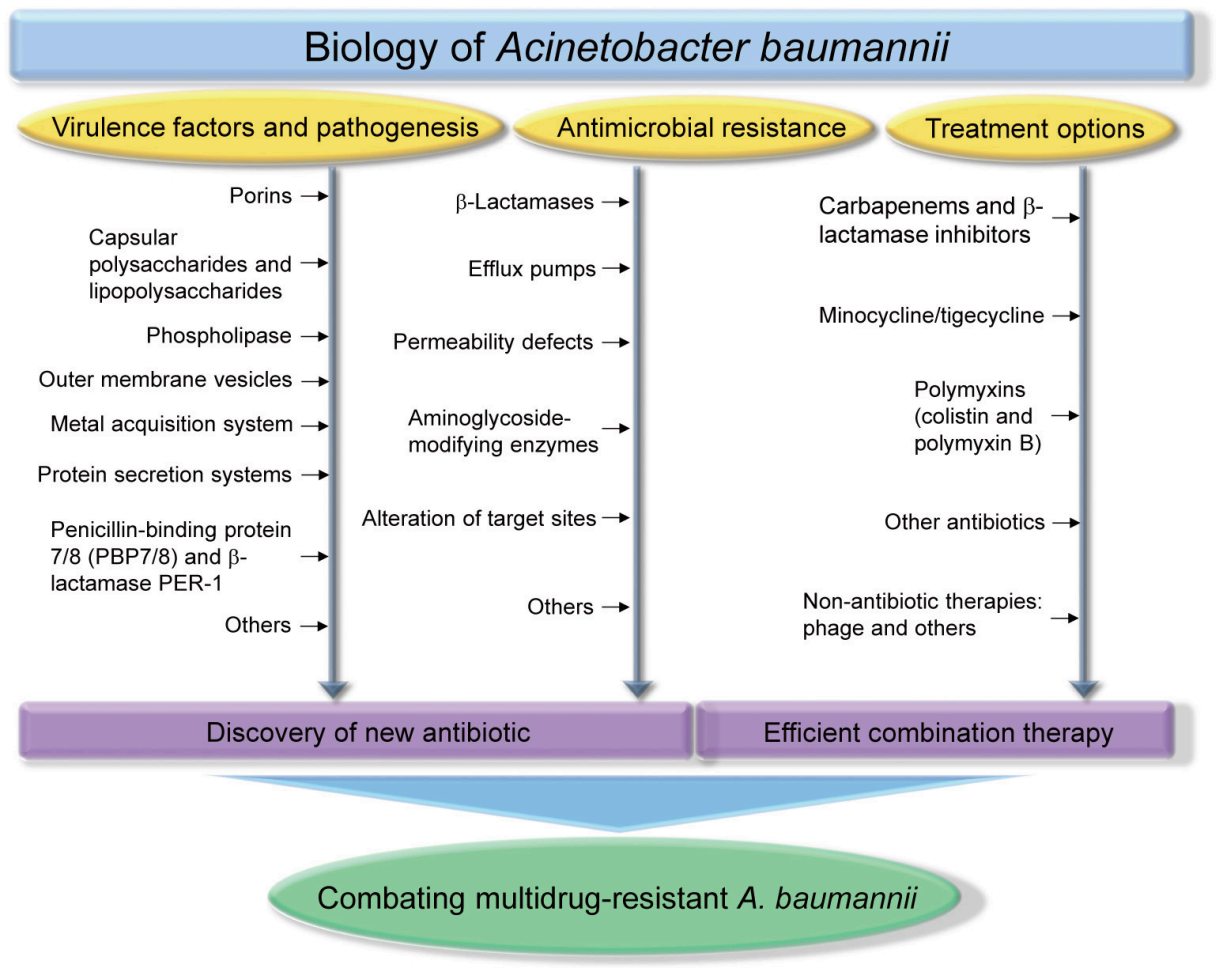
**Biology of *Acinetobacter baumannii***. Studies of virulence factors, pathogenesis, antimicrobial resistance, treatment options of *A. baumannii* will provide an important aid for discovering new antibiotics and determining efficient combination therapy, which are essential strategies for combating multidrug-resistant *A. baumannii* infections.

Many reports have shown that *A. baumannii* rapidly develops resistance to antimicrobials, and multidrug-resistant strains have been isolated (McConnell et al., 2013). The WHO declared that *A. baumannii* is one of the most serious ESKAPE organisms (*Enterococcus faecium, Staphylococcus aureus, Klebsiella pneumoniae, A. baumannii, Pseudomonas aeruginosa*, and *Enterobacter* species) that effectively escape the effects of antibacterial drugs (Boucher et al., 2009). A number of *A. baumannii* resistance mechanisms are known, including enzymatic degradation of drugs, target modifications, multidrug efflux pumps, and permeability defects (Gordon and Wareham, 2010; Kim et al., 2012; Lin and Lan, 2014). In this review, we summarize the virulence factors of *A. baumannii*, antibiotic resistance mechanisms, and the therapeutic options available for treating *A. baumannii* infections. Figure 1 depicts all the features described in this review.

## *Acinetobacter baumannii* virulence factors and pathogenesis

Although recent genomic and phenotypic analyses of *A. baumannii* have identified several virulence factors responsible for its pathogenicity, relatively few virulence factors have been identified in *A. baumannii*, compared to those in other Gram-negative pathogens (McConnell et al., 2013). The proposed *A. baumannii* virulence factors are summarized in Table 1.

**Table 1:** **Identified virulence factors of *Acinetobacter baumannii*.**.

**Virulence factor**	**Proposed role in pathogenesis**	**References**
Porin (OmpA, Omp33-36, Omp22, CarO, OprD-like)	Adherence and invasion, induction of apoptosis, serum resistance, biofilm formation, persistence	Choi et al., 2005, 2008b; Gaddy et al., 2009; Kim et al., 2009; Lee et al., 2010; Fernandez-Cuenca et al., 2011; Smani et al., 2012, 2013; Rumbo et al., 2014; Wang et al., 2014; Huang et al., 2016
Capsular polysaccharide	Growth in serum, survival in tissue infection, biofilm formation	Russo et al., 2010; Iwashkiw et al., 2012; Lees-Miller et al., 2013
Lipopolysaccharide (LPS)	Serum resistance, survival in tissue infection, evasion of the host immune response	Luke et al., 2010; Lin et al., 2012; McQueary et al., 2012; McConnell et al., 2013
Phospholipase (PLC and PLD)	Serum resistance, invasion, *in vivo* survival	Camarena et al., 2010; Jacobs et al., 2010; Stahl et al., 2015; Fiester et al., 2016
Outer membrane vesicle (OMV)	Delivery of virulence factors, horizontal transfer of antibiotic resistance gene	Kwon et al., 2009; Jin et al., 2011; Rumbo et al., 2011; Moon et al., 2012; Jun et al., 2013; Li Z. T. et al., 2015
Iron acquisition system (acinetobactin and NfuA)	*In vivo* survival, persistence, killing of host cells	Gaddy et al., 2012; Penwell et al., 2012; Zimbler et al., 2012; Fiester et al., 2016; Megeed et al., 2016
Zinc acquisition system (ZnuABC and ZigA)	*In vivo* survival	Hood et al., 2012; Nairn et al., 2016
Manganese acquisition system (MumC and MumT)	*In vivo* survival	Juttukonda et al., 2016
Type II protein secretion system	*In vivo* survival	Johnson et al., 2015; Elhosseiny et al., 2016; Harding et al., 2016
Type VI protein secretion system	Killing of competing bacteria, host colonization	Carruthers et al., 2013; Wright et al., 2014; Jones et al., 2015; Repizo et al., 2015; Ruiz et al., 2015
Type V protein secretion system	Biofilm formation, adherence	Bentancor et al., 2012b
Penicillin-binding protein 7/8 and β-lactamase PER-1	Serum resistance, *in vivo* survival, adherence	Sechi et al., 2004; Russo et al., 2009
CipA	Serum resistance, invasion	Koenigs et al., 2016
Tuf	Serum resistance	Koenigs et al., 2015
RecA	*In vivo* survival	Aranda et al., 2011
SurA1	Serum resistance, *in vivo* survival	Liu D. et al., 2016
GigABCD	*In vivo* survival, killing of host cells	Gebhardt et al., 2015
UspA	*In vivo* survival, killing of host cells	Elhosseiny et al., 2015; Gebhardt et al., 2015
GacS and PaaE	Neutrophil influx	Cerqueira et al., 2014; Gebhardt et al., 2015; Bhuiyan et al., 2016
Pili	Adherence, biofilm formation	Tomaras et al., 2003, 2008
OmpR/EnvZ	Killing of host cells	Tipton and Rather, 2016
FhaBC	Adherence, killing of host cells	Perez et al., 2016
AbeD	Killing of host cells	Srinivasan et al., 2015

### Porins

Porins are outer membrane proteins associated with modulating cellular permeability. OmpA is a β-barrel porin and one of the most abundant porins in the outer membrane. In *A. baumannii*, OmpA is the very well-characterized virulence factor with a variety of interesting biological properties identified in *in vitro* model systems (Smith et al., 2007; McConnell et al., 2013). A random mutagenesis screen showed that the *A. baumannii ompA* mutant is defective in inducing apoptosis in human epithelial cells (Choi et al., 2005). Purified OmpA binds host epithelial cells, targets mitochondria, and induces apoptosis by releasing proapoptotic molecules, such as cytochrome c and apoptosis-inducing factor (Choi et al., 2005; Lee et al., 2010). Another study showed that OmpA translocates to the nucleus by a novel monopartite nuclear localization signal and induces cell death (Choi et al., 2008a). OmpA also plays a major role in adherence and invasion of epithelial cells by interacting with fibronectin (Choi et al., 2008b; Gaddy et al., 2009; Smani et al., 2012), and binds to factor H in human serum (Kim et al., 2009), which may allow *A. baumannii* to avoid complement-mediated killing. The *ompA* gene is necessary for persistence of *A. baumannii* in the mouse lung (Wang et al., 2014).

Furthermore, OmpA is also involved in antimicrobial resistance of *A. baumannii* (Sugawara and Nikaido, 2012; Smani et al., 2014). The major *A. baumannii* porin is OmpA, which has 70-fold lower pore-forming activity than that of OmpF (Sugawara and Nikaido, 2012). Furthermore, disrupting the *ompA* gene significantly decreases the minimal inhibitory concentrations (MICs) of several antibiotics (chloramphenicol, aztreonam, and nalidixic acid), suggesting that OmpA participates in the extrusion of antibiotics from the periplasmic space through the outer membrane and couples with inner membrane efflux systems (Smani et al., 2014). OmpA enhances survival and persistence of *A. baumannii* by facilitating surface motility and biofilm formation (Gaddy et al., 2009; Clemmer et al., 2011; McConnell et al., 2013). OmpA also regulates biogenesis of outer membrane vesicles (Moon et al., 2012). These results suggest that the OmpA protein is an attractive target for developing novel antibiotics and prevention strategies. Two recent reports based on immuno-proteomics and reverse vaccinology suggested that OmpA is a potential vaccine candidate against *A. baumannii* (Fajardo Bonin et al., 2014; Hassan et al., 2016). Actually, the OmpA protein is immunogenic in healthy individuals and patients with *A. baumannii* invasive infections (Zhang et al., 2016). In a mouse model of *A. baumannii* infection, mice immunized with OmpA had a significantly higher survival rate than that of control mice (Luo et al., 2012; Lin L. et al., 2013; Zhang et al., 2016).

The 33- to 36-kDa Omp protein (Omp33-36), which acts as a water passage channel, is another outer membrane porin associated with *A. baumannii* cytotoxicity (Smani et al., 2013; Rumbo et al., 2014). The *omp33-36* deletion strain significantly reduces adherence and invasion of human lung epithelial cells and cytotoxicity to these cells (Smani et al., 2013). Deletion of the *omp33-36* gene in a murine sepsis model attenuates lethality and reduces bacterial concentrations in the spleen and lungs (Smani et al., 2013). One study showed that purified Omp33-36 induces apoptosis in several different cell types, including immune and connective tissue cells, by activating caspases and modulating autophagy (Rumbo et al., 2014). Omp33-36 is also involved in antibiotic resistance. *A. baumannii* strain JC10/01 resistant to carbapenem antibiotics (imipenem and meropenem) exhibits loss of Omp33-36 and episomal expression of Omp33-36 in this strain clearly reduces the MICs of imipenem and meropenem (del Mar Tomas et al., 2005).

Omp22 has also been identified as a novel, conserved, and safe antigen for developing effective vaccines to control *A. baumannii* infections (Huang et al., 2016), although the contribution of Omp22 to *A. baumannii* pathogenicity has not been determined. Both active and passive immunizations with Omp22 increase the survival rates of mice, suppress bacterial burdens in the organs and peripheral blood, and reduce serum levels of inflammatory cytokines and chemokines (Huang et al., 2016). Other porins, such as carbapenem-associated outer membrane protein (CarO) and OprD-like, are also virulence-related factors associated with attenuated virulence in a mouse model (Fernandez-Cuenca et al., 2011).

### Capsular polysaccharides and lipopolysaccharides (LPS)

Beyond OmpA, the *A. baumannii* envelope is associated with many factors that contribute to pathogenicity. Among these, capsular exopolysaccharides and LPS are *A. baumannii* pathogenicity factors. Notably, many isolates from patients with *A. baumannii* infections express surface capsular polysaccharides and contain a conserved gene cluster, called the K locus, which may determine production of capsular polysaccharides (Koeleman et al., 2001; Hu et al., 2013; Kenyon and Hall, 2013; Geisinger and Isberg, 2015). A random transposon screening to identify genes essential for growth in an inflammatory exudative fluid lead to the identification of the *ptk* and *epsA* genes, which are predicted to be required for capsule polymerization and assembly (Russo et al., 2010). The *ptk* and *epsA* mutants are deficient in capsule production and have a growth defect in human serum, resulting in a highly significant decrease in survival in soft tissue infection sites (Russo et al., 2010). Mutation in the *pglC* or *pglL* gene, which is responsible for synthesis of the *O*-pentasaccharide found on glycoproteins and capsular polysaccharides, also attenuate lethality in a mouse septicemia model and form abnormal biofilm structures (Iwashkiw et al., 2012; Lees-Miller et al., 2013). Therefore, capsular polysaccharides have been proposed to be a target for protective antibody-based interventions (passive immunization; Russo et al., 2013).

One study showed that capsular polysaccharides are involved in antimicrobial resistance of *A. baumannii* (Geisinger and Isberg, 2015). Mutants deficient in capsular polysaccharides have lower intrinsic resistance to peptide antibiotics. In addition, the presence of antibiotics induces hyperproduction of capsular polysaccharides (Geisinger and Isberg, 2015). Antibiotic-induced production of capsular polysaccharides increases resistance to killing by host complement and increases virulence in a mouse model of systemic infection (Geisinger and Isberg, 2015). That study also demonstrated that increased capsule production after exposure to an antibiotic depends on transcriptional increases in K locus gene expression, and that expression of K locus genes is regulated by the *bfmRS* two-component regulatory system (Geisinger and Isberg, 2015). *bfmR* is a gene essential for growth in human ascites, which is an *ex vivo* medium that reflects the infection environment (Umland et al., 2012), and is important for persistence in the lung in a murine pneumonia model (Wang et al., 2014). BfmS is also a virulence factor that plays an important role in biofilm formation, adherence to eukaryotic cells, and resistance to human serum (Liou et al., 2014). On report showed BfmR-mediated resistance to complement-mediated bactericidal activity and resistance to the clinically important antimicrobials (meropenem and colistin; Russo et al., 2016). However, that study suggested that BfmR effects are independent of capsular polysaccharide production. Therefore, the relationship between BfmRS and capsular polysaccharides must be described in more detail.

LPS is the major component of the outer leaflet of the outer membrane in most Gram-negative bacteria and is an immunoreactive molecule that induces release of tumor necrosis factor and interleukin 8 from macrophages in a Toll-like receptor 4 (TLR4)-dependent manner (Erridge et al., 2007). LPS is composed of an endotoxic lipid A moiety, an oligosaccharide core, and a repetitive O-antigen (Lee et al., 2013b). In *A. baumannii*, LPS plays a major role in virulence and survival of *A. baumannii* (Luke et al., 2010; Lin et al., 2012; McQueary et al., 2012). Mutant cell lacking LpsB glycotransferase have a highly truncated LPS glycoform containing only two carbohydrate residues bound to lipid A, resulting in decreased resistance to human serum and decreased survival in a rat model of soft tissue infection (Luke et al., 2010; McConnell et al., 2013). Inhibiting LpxC, an enzyme involved in the lipid A biosynthesis, dose not inhibit growth of the bacterium, but suppresses *A. baumannii* LPS-mediated activation of TLR4 (Lin et al., 2012). Inhibition of LpxC in mouse model enhances clearance of *A. baumannii* by enhancing opsonophagocytic killing and reduces serum LPS concentration and inflammation, which completely protects mice from lethal infection (Lin et al., 2012; Lee et al., 2013b). These results indicate that blocking LPS synthesis is a powerful strategy for discovering novel antibiotics. Modification of LPS contributes to resistance to antimicrobials. Many studies have shown that modifications in LPS decrease the susceptibility of *A. baumannii* to many clinical important antibiotics, such as colistin (Moffatt et al., 2010; Arroyo et al., 2011; Beceiro et al., 2011; Pelletier et al., 2013; Boll et al., 2015; Chin et al., 2015).

### Phospholipase

Phospholipase is a lipolytic enzyme essential for phospholipid metabolism and is a virulence factor in many bacteria, such as *P. aeruginosa, Legionella monocytogenes*, and *Clostridium perfringens* (Camarena et al., 2010; Flores-Diaz et al., 2016). Three classes of phospholipases, such as phospholipase A (PLA), phospholipase C (PLC), and phospholipase D (PLD) have been defined based on the cleavage site. PLA hydrolyzes fatty acids from the glycerol backbone, whereas PLC cleaves the phosphorylated head group from the phospholipid. PLD is a transphosphatidylase that only cleaves off the head group. Degradation of phospholipids affects the stability of host cell membranes, and the cleaved head group can interfere with cellular signaling, resulting in changes in the host immune response (Songer, 1997; Flores-Diaz et al., 2016). PLC and PLD have been identified as virulence factors in *A. baumannii* (Camarena et al., 2010; Jacobs et al., 2010; Stahl et al., 2015). *Acinetobacter baumannii* ATCC17978 has two PLCs (A1S_0043 and A1S_2055) and inactivation of the A1S_0043 gene leads to a modest reduction in the cytotoxic effect of *A. baumannii* on epithelial cells compared to that of the parental strain (Camarena et al., 2010; Fiester et al., 2016). Disrupting one (A1S_2989) of the two PLD genes present in *A. baumannii* strain 98-37-09 results in reduced resistance to human serum, decreased capacity for invading epithelial cells, and decreased virulence in a murine model of pneumonia (Jacobs et al., 2010). Another report showed that *A. baumannii* ATCC 19606 has three PLD genes and all three play important roles in virulence and host cell invasion in a concerted manner (Stahl et al., 2015). These results suggest that phospholipase enzymes are important virulence factors in *A. baumannii* pathogenesis.

### Outer membrane vesicles (OMVs)

OMVs are spherical, 20–200 nm diameter vesicles secreted by the outer membranes of various Gram-negative pathogenic bacteria (Kulp and Kuehn, 2010). They are composed of LPS, outer membrane and periplasmic proteins, phospholipids, and DNA or RNA, and are recognized as delivery vehicles for bacterial effectors to host cells (Ellis and Kuehn, 2010). OMVs deliver diverse virulence factors to the interior of host cells simultaneously and allow the pathogens to interact with the host without close contact between bacteria and host cells (Jun et al., 2013). Many *A. baumannii* strains secrete OMVs containing various virulence factors, including OmpA (Kwon et al., 2009; Jin et al., 2011; Moon et al., 2012), proteases (Kwon et al., 2009), and phospholipases (Kwon et al., 2009). OMVs derived from *A. baumannii* interact with host cells and deliver bacterial effectors to host cells via lipid rafts, resulting in cytotoxicity (Jin et al., 2011). Purified OMVs of *A. baumannii* ATCC 19606 induce expression of pro-inflammatory cytokine genes in epithelial cells in a dose-dependent manner (Jun et al., 2013). Notably, OMVs treated with proteinase do not induce a significant increase in the expression of pro-inflammatory cytokine genes, suggesting that the membrane proteins in OMVs are responsible for eliciting a potent innate immune response (Jun et al., 2013). One study supports the role of OMVs in *A. baumannii* pathogenesis. An *A. baumannii* strain that produces abundant OMVs with more virulence factors induces a stronger innate immune response and is more cytotoxic compared with those of a strain producing fewer OMVs (Li Z. T. et al., 2015).

Due to the importance of OMVs in *A. baumannii* virulence, several reports have shown that *A. baumannii* OMVs could be used as an acellular vaccine to elevate protective immunity (McConnell et al., 2011; Huang et al., 2014). In a mouse model of disseminated sepsis, vaccination with *A. baumannii* ATCC 19606 strain OMVs protects mice from challenge with homologous bacteria and provides protection against other clinical isolates (McConnell et al., 2011). Similar results were obtained in a pneumonia mouse model. Bacterial burden, inflammatory cell infiltration, and inflammatory cytokine accumulation in the pneumonia model were significantly suppressed by both active and passive immunization with OMVs (Huang et al., 2014). These results indicate that *A. baumannii* OMVs can be used as an acellular vaccine to effectively control *A. baumannii* infections. Interestingly, *A. baumannii* OMVs are also related with the spread of antibiotic resistance and induce the horizontal transfer of the OXA-24 carbapenemase gene (Rumbo et al., 2011).

### Metal acquisition system

Although iron is one of the most abundant elements in environmental and biological systems, ferric iron is relatively unavailable to bacteria in the preferred state, because of its poor solubility (10^−17^ M solubility limit for ferric iron) under aerobic and neutral pH conditions as well as due to chelation by low-molecular-weight compounds, such as heme, or high-affinity iron-binding compounds, such as lactoferrin and transferrin (Rakin et al., 2012; Saha et al., 2013). To overcome this iron limitation, most aerobic bacteria produce a high-affinity iron chelator known as a siderophore (Saha et al., 2013). Siderophores are low molecular weight compounds (400–1,000 kDa) with high affinity for iron. The range of Fe^3+^-siderophore association constants is 10^12^–10^52^ (Saha et al., 2013). Siderophores have been classified into catecholates, hydroxymates, and a mixed type based on the moiety that donates oxygen ligands to coordinate Fe^3+^ (Saha et al., 2013). *Acinetobacter baumannii* also has iron siderophores and acinetobactin, the best-characterized *A. baumannii* siderophore, is a mixed type siderophore with an oxazoline ring derived from threonine (McConnell et al., 2013). Acinetobactin is an *A. baumannii* virulence factor (Gaddy et al., 2012; Penwell et al., 2012; Megeed et al., 2016). Impaired acinetobactin biosynthesis and transport functions significantly reduce the ability of *A. baumannii* ATCC 19606 cells to persist within epithelial cells and cause cell damage and animal death (Gaddy et al., 2012). Mutation in the *entA* gene, which is essential for biosynthesis of the acinetobactin precursor 2,3-dihydroxybenzoic acid, also significantly reduces the capacity of *A. baumannii* ATCC 19606 cells to persist within human alveolar epithelial cells and diminishes the ability to infect and kill *Galleria mellonella* larvae (Penwell et al., 2012). One study showed that acinetobactin production occurs significantly more frequently in MDR *A. baumannii* isolates than that in avirulent isolates (Megeed et al., 2016).

The *A. baumannii* NfuA Fe-S scaffold protein, that participates in the formation of Fe-S clusters and plays a role in cell responses to iron chelation and oxidative stress, has also been identified as a virulence factor (Zimbler et al., 2012). The *nfuA* mutant is more sensitive to reactive oxygen species (ROS), such as hydrogen peroxide and cumene hydroperoxide, and shows significantly reduced growth in human epithelial cells. In addition, a *G. mellonella* infection model showed that more than 50% of injected *G. mellonella* larvae die 6 days after infection with the parental strain, whereas less than 30% of the larvae die when infected with the *nfuA* mutant (Zimbler et al., 2012). One report showed that iron starvation increases production of PLCs, which increase hemolytic activity of *A. baumannii* (Fiester et al., 2016). These reports indicate that iron acquisition functions play a critical role in *A. baumannii* virulence.

The innate immune metal-chelating protein calprotectin inhibits bacterial growth by host-mediated chelation of metals, such as zinc (Zn^2+^ and Zn) and manganese (Mn^2+^ and Mn) (Corbin et al., 2008). However, *A. baumannii* can cause disease in the presence of this nutritional immune protein *in vivo* (Juttukonda et al., 2016). To combat the zinc limitation, *A. baumannii* uses a zinc acquisition system (ZnuABC), which is up-regulated under Zn-limiting conditions, and the *znuB* mutant strain experiences Zn starvation at higher Zn concentrations than that of the wild-type (Hood et al., 2012). ZnuB contributes to the pathogenesis of *A. baumannii* pulmonary infections. Notably, a zinc limitation reduces the imipenem MIC of MDR *A. baumannii* to below the clinical breakpoint for imipenem resistance in *A. baumannii* (Hood et al., 2012), possibly because many carbapenemases are metalloenzymes that require Zn for their hydrolyzing activity. Besides the ZnuABC system, the novel Zn metallochaperone ZigA has been characterized in *A. baumannii* (Nairn et al., 2016). ZigA tightly interacts with Zn, which is required for bacterial growth under Zn starvation conditions and for disseminated infection in mice (Nairn et al., 2016).

The mechanism employed by *A. baumannii* to overcome a Mn limitation has been identified. Calprotectin induces Mn starvation in *A. baumannii*, which increases transcription of an NRAMP (Natural Resistance-Associated Macrophage Proteins) family Mn transporter and a urea carboxylase to resist the antimicrobial activities of calprotectin (Juttukonda et al., 2016). A urea carboxylase enzyme (MumC) is important for growth of *A. baumannii* in the presence of calprotectin and an NRAMP family transporter (MumT) contributes to the fitness of *A. baumannii* in a murine pneumonia model (Juttukonda et al., 2016), suggesting that the two proteins are virulence factors. *Acinetobacter baumannii* can utilize urea as a sole nitrogen source, and this urea utilization is required for MumC (Juttukonda et al., 2016). Based on the contribution of MumC to *A. baumannii* resistance to calprotectin, the authors suggest a connection between metal starvation and metabolic stress, such as nitrogen starvation.

### Protein secretion systems

Several protein secretion systems have been identified in *A. baumannii* (Weber et al., 2015a). The most recently described *A. baumannii* secretion system is a type II secretion system (T2SS) (Johnson et al., 2015). The T2SS is a multi-protein complex that is structurally very similar to type IV pili systems, which is an appendage that is commonly found in Gram-negative bacteria (Korotkov et al., 2012). T2SS translocates a wide range of proteins from the periplasmic space to the extracellular milieu out of the cell or the outer membrane surface. The T2SS is composed of 12–15 proteins comprised of four sub-assemblies: a pseudopilus, a cytoplasmic secretion ATPase, an inner-membrane platform assembly, and a dodecameric outer-membrane complex (Korotkov et al., 2012; Harding et al., 2016). Secretion by T2SS is a two-step process. The target proteins are first translocated to the periplasm by the general secretory (Sec) system or the twin arginine transport (Tat) system, where the target proteins are then secreted out of the cell through the T2SS (Korotkov et al., 2012). Deleting *A. baumannii* genes for the T2SS components, *gspD* or *gspE*, results in loss of LipA secretion, indicating that LipA is a T2SS substrate (Johnson et al., 2015). Because LipA is a lipase that breaks down long-chain fatty acids, *lipA, gspD*, and *gspE* mutant strains are incapable of growing on long-chain fatty acids as a sole carbon source and are defective in *in vivo* growth in a neutropenic murine model of bacteremia (Johnson et al., 2015). The role of a functional T2SS for full virulence of *A. baumannii* has been shown in *G. mellonella* and murine pulmonary infection models (Harding et al., 2016). Lipases (LipA, LipH, and LipAN) and the metallopeptidase CpaA have been identified as T2SS substrates (Elhosseiny et al., 2016; Harding et al., 2016). Notably, two proteins (LipA and CpaA) among these secreted proteins require specific chaperones for secretion. These chaperones are encoded adjacently to their cognate effector, and their inactivation abolishes secretion of LipA and CpaA (Harding et al., 2016).

*Acinetobacter baumannii* also has a type VI secretion system (T6SS). The T6SS was first identified in *Vibrio cholera* and *P. aeruginosa* (Mougous et al., 2006; Pukatzki et al., 2006). Many bacteria use the T6SS to inject effector proteins, providing a colonization advantage during infection of eukaryotic hosts (Mougous et al., 2006) or to kill competing bacteria (Basler et al., 2013). The T6SS leads to DNA release and horizontal gene transfer in *V. cholera*, which may contribute to spread of antibiotic resistance (Borgeaud et al., 2015). The T6SS is composed of many conserved structural proteins and accessory factors, and bears a contractile bacteriophage sheath-like structure forming a needle or spike structure used to penetrate the target cell (Shneider et al., 2013). Hcp is a structural protein forming a polymerized tubular structure that is secreted out of the cell, and VgrGs are involved in attaching effector domains to the spike, and a proline-alanine-alanine-arginine (PAAR) repeat protein forms the sharp tip of the distinctive needle-like structure (Shneider et al., 2013; Zoued et al., 2014).

The presence of T6SS in *A. baumannii* was initially predicted by bioinformatic analysis (Weber et al., 2013). Although the role of T6SS in *A. baumannii* ATCC 17978 has not been determined (Weber et al., 2013), research on *A. baumannii* strain M2 showed that this strain produces a functional T6SS and that the T6SS mediates killing of competing bacteria (Carruthers et al., 2013). Another study showed that the T6SS is active in six pathogenic strains of *A. baumannii* (Ruiz et al., 2015). However, the T6SS seems to play an important role in *A. baumannii* virulence in a strain-specific manner (Repizo et al., 2015). They compared T6SS functionality of several *A. baumannii* strains, including ATCC17978 (a type strain), various MDR strains implicated in hospital outbreaks (Ab242, Ab244, and Ab825), and DSM30011 (a non-clinical isolate). Although the T6SS genomic locus is present in all of these strains, only DSM30011 has a fully active T6SS that mediates *E. coli* killing (Repizo et al., 2015). In addition, the T6SS of DSM30011 is required for host colonization of the *G. mellonella* model organism (Repizo et al., 2015). Similar results were obtained from a comparative analysis of the genomes of MDR *A. baumannii* clinical strains (Wright et al., 2014; Jones et al., 2015). *A. baumannii* isolates of a particular clade exhibit complete loss of the T6SS genomic locus. Therefore, these results suggest that more extensive investigations are required to analyze the role of T6SS in *A. baumannii* virulence, even though this system seems to play an important role in *A. baumannii* virulence in some strains. Notably, one study showed that several MDR *A. baumannii* strains have a large, self-transmissible plasmid that carries negative regulators for T6SS (Weber et al., 2015b). The T6SS is silenced in plasmid-containing, antibiotic-resistant cells, whereas plasmid-losing cells have an active T6SS. Although plasmid-losing cells are capable of T6SS-mediated killing of competing bacteria, they become susceptible to antibiotics (Weber et al., 2015b). This result suggests a molecular switch between T6SS and antibiotic resistance.

The type V system autotransporter Ata has also been characterized in *A. baumannii* (Bentancor et al., 2012a). This is a trimeric membrane protein that mediates biofilm formation, adherence to extracellular matrix components such as collagen I, III, and IV, and virulence in a murine systemic model of *Acinetobacter* infection (Bentancor et al., 2012a). Another experiment using a pneumonia model of infection in immunocompetent and immunocompromised mice showed that Ata is a vaccine candidate against *A. baumannii* infections (Bentancor et al., 2012b). A type IV secretion system present in the plasmid was bioinformatically identified in *A. baumannii* (Liu C. C. et al., 2014), but no experimental evidence describing its function has been presented.

### Penicillin-binding protein 7/8 (PBP7/8) and β-lactamase PER-1

Although PBPs are commonly involved in resistance to β-lactam antibiotics, PBP7/8 encoded by the *pbpG* gene is a virulence factor in *A. baumannii*. The *pbpG* mutant strain grows similar to its wild-type strain in Luria-Bertani medium, but the mutant shows reduced growth in human serum and its survival significantly decreases in rat soft-tissue infection and pneumonia models (Russo et al., 2009). An investigation of bacterial morphology using electron microscopy suggested that loss of PBP7/8 may have affected peptidoglycan structure, which may affect susceptibility to host defense factors (Russo et al., 2009).

Interestingly, β-lactamase PER-1 has been suggested to be an *A. baumannii* virulence factor. PER-1 is an extended-spectrum-β-lactamase (ESBL), but this gene is associated with cell adhesion (Sechi et al., 2004). Nine PER-1-producing strains adhere to the Caco2 cell lines, whereas all PER-1-negative strains are negative for cell adhesion (Sechi et al., 2004). Notably, many β-lactamases are associated with virulence in various pathogenic bacteria, such as *E. coli* (Dubois et al., 2009), *P. aeruginosa* (Moya et al., 2008), and *K. pneumoniae* (Sahly et al., 2008). However, no general mechanisms have been proposed (Beceiro et al., 2013).

### Others

*Acinetobacter baumannii* CipA is a novel plasminogen binding and complement inhibitory protein that mediates serum resistance (Koenigs et al., 2016). CipA-binding plasminogen is converted to active plasmin that degrades fibrinogen and complement C3b, which contributes to serum resistance of *A. baumannii*. Therefore, the *cipA* mutant strain is efficiently killed by human serum and also shows a defect in the penetration of endothelial monolayers (Koenigs et al., 2016). Similar to CipA, the *A. baumannii* translation elongation factor Tuf is also a plasminogen-binding protein. Tuf-binding plasminogen can be converted to active plasmin, which proteolytically degrades fibrinogen as well as component C3b (Koenigs et al., 2015). RecA, which is involved in homologous recombination and the SOS response, has been identified as an *A. baumannii* virulence factor. The *recA* mutant shows significantly reduced survival within macrophages and decreases lethality in a mouse model of systemic infection (Aranda et al., 2011). The surface antigen protein 1 (SurA1) plays an important role in fitness and virulence of *A. baumannii* (Liu D. et al., 2016). Serum resistance of the *surA1* mutant significantly decreases compared with that of the wild-type strain CCGGD201101. In the *G. mellonella* insect model, a *surA1* mutant strain exhibits a lower survival rate and decreased dissemination (Liu D. et al., 2016).

A growth analysis of 250,000 *A. baumannii* transposon mutants within *G. mellonella* larvae identified 300 genes required for survival or growth of *A. baumannii* inside *G. mellonella* larvae (Gebhardt et al., 2015). The 300 genes were classified into six categories of micronutrient acquisition, cysteine metabolism/sulfur assimilation, aromatic hydrocarbon metabolism, cell envelope/membrane/wall, stress response genes, antibiotic resistance, and transcriptional regulation. Among them, four transcriptional regulators required for growth in *G. mellonella* larvae were called the *gig* (growth in *Galleria*) genes. Loss of these genes (*gigA*-*D*) led to a significant defect in both growth within and killing of *G. mellonella* larvae (Gebhardt et al., 2015). This study identified stress proteins, such as UspA, as factors required for growth in *G. mellonella*. Another study showed that UspA is essential for pneumonia and sepsis pathogenesis of *A. baumannii* (Elhosseiny et al., 2015). Among the 300 genes, several genes are involved in aromatic hydrocarbon metabolism (Gebhardt et al., 2015). Another study showed that GacS, which is a transcriptional factor that regulates expression of genes, such as *paaE*, and is responsible for the phenylacetic acid catabolic pathway, affects *A. baumannii* virulence (Cerqueira et al., 2014). Experiments using a *paaE* deletion mutant confirmed the role of aromatic hydrocarbon metabolism in *A. baumannii* virulence (Cerqueira et al., 2014), but its molecular mechanism remains unknown. Interestingly, a recent report showed that accumulation of phenylacetate in *A. baumannii* induces rapid neutrophil influx to a localized site of infection and increases bacterial clearance (Bhuiyan et al., 2016). They suggested that phenylacetate is a neutrophil chemoattractant inducing bacterial-guided neutrophil chemotaxis. This report may reveal a novel molecular mechanism about the role of the phenylacetic acid catabolic pathway in *A. baumannii* virulence.

Biofilm formation plays an important role in immune evasion by *A. baumannii* (de Breij et al., 2010), and pili are essential for *A. baumannii* adherence to and biofilm formation on abiotic surfaces as well as virulence (Tomaras et al., 2003, 2008). Notably, imipenem treatment of the imipenem-resistant *A. baumannii* isolate induces expression of important genes responsible for synthesis of type IV pili (Dhabaan et al., 2015), suggesting that the ability to overproduce pili confers a biological advantage to *A. baumannii*.

Other virulence-related proteins have been identified, including OmpR/EnvZ (Tipton and Rather, 2016), FhaBC (Perez et al., 2016), and the resistance-nodulation-division-type membrane transporter AbeD (Srinivasan et al., 2015), but their molecular mechanisms remain unknown.

## Antimicrobial resistance of *A. baumannii*

*Acinetobacter baumannii* has become one of the most successful pathogens in modern healthcare because of its amazing ability to acquire antimicrobial resistance. Several strains of *A. baumannii* are highly resistant to most clinically available antibiotics (Lin and Lan, 2014). *A. baumannii* has a number of resistance mechanisms, including β-lactamases, aminoglycoside-modifying enzymes, efflux pumps, permeability defects, and modifications of target sites. The accumulation of several resistance mechanisms in *A. baumannii* has gradually decreased the number of antibiotic classes available to treat *A. baumannii* infections in clinical practice. Table 2 shows the antibiotic resistance mechanisms found in *A. baumannii*. We will discuss the details below.

**Table 2:** **Resistance mechanisms in *Acinetobacter baumannii***.

**Resistance mechanism**	**Class/subgroup**	**Protein**	**References**
β-Lactamases	Class A	TEM-1	Chen et al., 2006; Adams et al., 2008; Krizova et al., 2013
		TEM-92	Endimiani et al., 2007
		GES-1	Al-Agamy et al., 2016
		GES-5	Al-Agamy et al., 2016
		GES-11	Moubareck et al., 2009; Bogaerts et al., 2010; Chihi et al., 2016
		GES-12	Bogaerts et al., 2010
		GES-14	Bogaerts et al., 2010
		PER-1	Jeong et al., 2005; Poirel et al., 2005a; Aly et al., 2016
		PER-2	Pasteran et al., 2006
		PER-7	Bonnin et al., 2011b
		CTX-M-2	Nagano et al., 2004
		CTX-M-15	Potron et al., 2011
		SCO-1	Poirel et al., 2007
		VEB-1	Fournier et al., 2006; Naas et al., 2006; Pasteran et al., 2006; Adams et al., 2008; Poirel et al., 2009
		KPC-2	Martinez et al., 2016
		KPC-10	Robledo et al., 2010
		CARB-4	Ramirez et al., 2010b
		CARB-10	Potron et al., 2009
	Class B	IMP-1	Tognim et al., 2006
		IMP-2	Riccio et al., 2000
		IMP-4	Chu et al., 2001
		IMP-5	Koh et al., 2007
		IMP-6	Gales et al., 2003
		IMP-8	Lee M. F. et al., 2008
		IMP-11	Yamamoto et al., 2011
		IMP-19	Yamamoto et al., 2011
		IMP-24	Lee M. F. et al., 2008
		VIM-1	Tsakris et al., 2006, 2008; Papa et al., 2009
		VIM-2	Yum et al., 2002; Lee M. F. et al., 2008
		VIM-3	Lee M. F. et al., 2008
		VIM-4	Tsakris et al., 2008; Papa et al., 2009
		VIM-11	Lee M. F. et al., 2008
		NDM-1	Chen et al., 2011; Pfeifer et al., 2011; Bonnin et al., 2012; Voulgari et al., 2016
		NDM-2	Espinal et al., 2011
		NDM-3	Kumar, 2016
		SIM-1	Lee et al., 2005
	Class C	AmpC	Bou and Martinez-Beltran, 2000; Corvec et al., 2003; Segal et al., 2004; Hujer et al., 2005; Heritier et al., 2006; Liu and Liu, 2015
	Class D		
	OXA-2 subgroup	OXA-21	Vila et al., 1997
	OXA-10 subgroup	OXA-128	Giannouli et al., 2009
	OXA-20 subgroup	OXA-37	Navia et al., 2002
	OXA-23 subgroup	OXA-23	Heritier et al., 2005b; Naas et al., 2005; Corvec et al., 2007; Koh et al., 2007; Perez et al., 2007; Valenzuela et al., 2007; Wang et al., 2007; Adams et al., 2008; Stoeva et al., 2008; Kohlenberg et al., 2009; Kuo et al., 2010; Mugnier et al., 2010; Bonnin et al., 2011a; Lee et al., 2011; Lin et al., 2011b; Koh et al., 2012; Mosqueda et al., 2013; Chagas et al., 2014; Principe et al., 2014; Li Y. et al., 2015
		OXA-133	Mendes et al., 2009
		OXA-239	Gonzalez-Villoria et al., 2016
	OXA-24 subgroup	OXA-24	Bou et al., 2000b; Merino et al., 2010; Acosta et al., 2011; Pailhories et al., 2016
		OXA-25, OXA-26, OXA-27	Afzal-Shah et al., 2001
		OXA-40	Heritier et al., 2003; Lolans et al., 2006; Quinteira et al., 2007; Ruiz et al., 2007
		OXA-72	Wang et al., 2007; Lu et al., 2009; Goic-Barisic et al., 2011; Dortet et al., 2016; Kuo et al., 2016
		OXA-143	Higgins et al., 2009
		OXA-182	Kim et al., 2010
	OXA-51 subgroup	OXA-51	Brown et al., 2005; Hu et al., 2007; Ruiz et al., 2007; Adams et al., 2008; Chen et al., 2010; Fang et al., 2016
		OXA-64, OXA-65, OXA-66, OXA-68, OXA-70, OXA-71	Hamouda et al., 2010; Biglari et al., 2016
		OXA-69, OXA-75, OXA-76, OXA-77	Heritier et al., 2005a
		OXA-79, OXA-80, OXA-104, OXA-106~ OXA-112	Evans et al., 2007
		OXA-82, OXA-83, OXA-83, OXA-84	Turton et al., 2006b; Evans et al., 2007
		OXA-86, OXA-87	Vahaboglu et al., 2006
		OXA-88, OXA-91, OXA-93, OXA-94, OXA-95, OXA-96	Koh et al., 2007
		OXA-92	Tsakris et al., 2007
		OXA-113	Naas et al., 2007
	OXA-58 subgroup	OXA-58	Dijkshoorn et al., 1996; Poirel et al., 2005b; Pournaras et al., 2006; Chen et al., 2008; Qi et al., 2008; Donnarumma et al., 2010; Gogou et al., 2011; Ravasi et al., 2011; Hou and Yang, 2015
		OXA-96	Koh et al., 2007
		OXA-97	Poirel et al., 2008
	OXA-143 subgroup	OXA-253	de Sa Cavalcanti et al., 2016
	OXA-235 subgroup	OXA-235	Higgins et al., 2013
Efflux pumps	Resistance-nodulation-division superfamily	AdeABC	Magnet et al., 2001; Marchand et al., 2004; Peleg et al., 2007; Ruzin et al., 2007; Lin et al., 2015; Sun et al., 2016
		AdeFGH	Coyne et al., 2010; He X. et al., 2015
		AdeIJK	Damier-Piolle et al., 2008
	Major facilitator superfamily	TetA	Ribera et al., 2003a
		TetB	Vilacoba et al., 2013
		CmlA	Coyne et al., 2011
		CraA	Roca et al., 2009
		AmvA	Rajamohan et al., 2010
		AbaF	Sharma et al., 2016
	Multidrug and toxic compound extrusion family	AbeM	Su et al., 2005
	Small multidrug resistance family	AbeS	Srinivasan et al., 2009
	Other efflux pumps	EmrAB-TolC	Nowak-Zaleska et al., 2016
		A1S_1535, A1S_2795, and ABAYE_0913	Li L. et al., 2016
Permeability defects	Porin	OmpA	Smani et al., 2014; Wu et al., 2016
		CarO	Mussi et al., 2005, 2007; Siroy et al., 2005; Catel-Ferreira et al., 2011; Jin et al., 2011
		Omp22-33	Bou et al., 2000a
		Omp33-36	del Mar Tomas et al., 2005
		Omp37	Quale et al., 2003
		Omp43	Dupont et al., 2005
		Omp44	Quale et al., 2003
		Omp47	Quale et al., 2003
Aminoglycoside-modifying enzymes	Aminoglycoside acetyltransferases	AAC3 (*aacC1, aacC2*)	Nemec et al., 2004
		AAC(6′) (*aacA4*)	Doi et al., 2004; Cho et al., 2009; Zhu et al., 2009; Lin et al., 2010; Lin M. F. et al., 2013; Bakour et al., 2014
	Aminoglycoside adenyltransferases	ANT(2″) (*aadB*)	Nemec et al., 2004
		ANT(3″) (*aadA1*)	Cho et al., 2009; Lin et al., 2010; Lin M. F. et al., 2013
	Aminoglycoside phosphotransferases	APH(3′) (*aphA1*)	Gallego and Towner, 2001
		APH(3″)	Cho et al., 2009
Alteration of target sites	Change of penicillin binding protein(PBP)	PBP2	Gehrlein et al., 1991
	16S rRNA methylation	ArmA	Yu et al., 2007; Cho et al., 2009; Karthikeyan et al., 2010; Brigante et al., 2012; Hong et al., 2013; Bakour et al., 2014; Tada et al., 2014
	Ribosomal protection	TetM	Ribera et al., 2003b
	DNA gyrase	GyrA/ParC	Higgins et al., 2004
	Dihydrofolate reductase	DHFR	Mak et al., 2009; Lin M. F. et al., 2013
		FolA	Mak et al., 2009
	Lipopolysaccharide	PmrC, LpxA, LpxC, LpxD	Adams et al., 2009; Moffatt et al., 2010; Arroyo et al., 2011
Other mechanisms	S-adenosyl-L-methionine-dependent methyltransferase	Trm	Chen et al., 2014; Trebosc et al., 2016
	1-Acyl-sn-3-phosphate acyltransferase	PlsC	Li X. et al., 2015
	Peptidase C13 family	Abrp	Li X. et al., 2016
	Cell division proteins	BlhA, ZipA, ZapA, and FtsK	Knight et al., 2016
	SOS response	RecA	Aranda et al., 2011, 2014; Norton et al., 2013

### β-lactamases

Inactivation of β-lactams by β-lactamases is a major antibiotic resistance mechanism in *A. baumannii*. Based on sequence homology, β-lactamases are grouped into molecular classes, A, B, C, and D (Jeon et al., 2015). All four classes of β-lactamases were identified in *A. baumannii*. Recent studies have shown that *A. baumannii* has natural competence to incorporate exogenous DNA and its genome has foreign DNA at high frequencies, implying frequent horizontal gene transfer in this pathogen (Ramirez et al., 2010a; Touchon et al., 2014; Traglia et al., 2014). Additionally, albumin, a main protein in blood, enhances natural competence of *A. baumannii* (Traglia et al., 2016). Therefore, natural competence of *A. baumannii* may contribute to identification of a large number of β-lactamases in this threatening human pathogen.

Class A β-lactamases inhibited by clavulanate hydrolyze penicillins and cephalosporins more efficiently than carbapenems, except for some KPC type enzymes (Jeon et al., 2015). A number of class A β-lactamases, including TEM, SHV, GES, CTX-M, SCO, PER, VEB, KPC, and CARB, have been identified in *A. baumannii* (Table 2). Some of these enzymes, such as TEM-1, CARB-4, and SCO-1, are narrow-spectrum β-lactamases, whereas other enzymes (e.g., PER-1, TEM-92, CARB-10, SHV-5, PER-2, CTX-M-2, CTX-M-15, VEB-1, GES-14, and PER-7) are ESBLs. Some carbapenemases, such as GES-14 and KPC-2, have been detected in *A. baumannii* (Moubareck et al., 2009; Bogaerts et al., 2010).

Unlike the serine-dependent β-lactamases (classes A, C, and D), class B β-lactamases are metallo-β-lactamases (MBLs) that require zinc or another heavy metal for catalysis (Jeon et al., 2015). Due to a broad substrate spectrum, MBLs catalyze the hydrolysis of virtually all β-lactam antibiotics including carbapenems, but not monobactams (Jeon et al., 2015). A variety of class B β-lactamases have been identified in *A. baumannii* (Table 2).

Class C β-lactamases pose therapeutic problems because they can confer resistance to cephamycins (cefoxitin and cefotetan), penicillins, cephalosporins, and β-lactamase inhibitor combinations, but are not significantly inhibited by clinically used β-lactamase inhibitors, such as clavulanic acid (Jeon et al., 2015). *Acinetobacter baumannii* has an intrinsic AmpC cephalosporinase (Gordon and Wareham, 2010). An analysis of 23 MDR *A. baumannii* clinical isolates in Taiwan showed that all isolates had AmpC-type β-lactamases (Lin et al., 2011a). Several clinical isolates of *A. baumannii* have the *ampC* gene transcribed from a strong promoter contained within a putative insertion sequence element (IS*Aba1*-like sequence), which results in high resistance to ceftazidime (Corvec et al., 2003; Segal et al., 2004). This sequence has been identified in ceftazidime-resistant *A. baumannii* isolates, but is absent in ceftazidime-susceptible *A. baumannii* isolates (Heritier et al., 2006).

Class D β-lactamases are called OXAs (oxacillinases), because they commonly hydrolyze isoxazolylpenicillin oxacillin much faster than benzylpenicillin (Jeon et al., 2015). More than 400 OXA-type enzymes have been identified and many variants actually possess carbapenemase activity. The presence of carbapenem-hydrolyzing class D β-lactamases or MBLs is one of the major carbapenem resistance mechanisms in *A. baumannii* (Lin and Lan, 2014). The subgroups of carbapenem-hydrolyzing OXAs, such as the OXA-23, OXA-24, OXA-51, and OXA-58 subgroups, are prevalent in *A. baumannii* (Table 2). The OXA-23 enzyme was first identified in an *A. baumannii* isolate in the United Kingdom in 1985 (Perez et al., 2007). The *bla*_OXA-23_ gene has been disseminated worldwide, and the frequency of OXA-23-producing *A. baumannii* strains is significantly high (Mugnier et al., 2010; Al-Agamy et al., 2016). One recent report from Lebanon showed 76.5% of 119 *A. baumannii* isolates are resistant to carbapenems, and OXA-23 β-lactamases have been found in 82 isolates (Al Atrouni et al., 2016). Insertion of IS*Aba1* in the *bla*_OXA-23_ promoter sequence has been reported to be associated with overexpression of *bla*_OXA-23_, *bla*_OXA-51_, or *bla*_OXA-58_ in *A. baumannii* (Turton et al., 2006a). One report from India showed that *bla*_OXA-51_ and *bla*_OXA-23_ were present in all 103 carbapenem-resistant *A. baumannii* isolates and almost 80% of the isolates had IS*Aba1* upstream of the *bla*_OXA-23_ gene, indicating the prevalence of the IS*Aba1* insertion (Vijayakumar et al., 2016).

### Efflux pumps

Efflux pumps are associated with resistance against many different classes of antibiotics, such as imipenem (Hu et al., 2007) and tigecycline (Peleg et al., 2007; Ruzin et al., 2007), in *A. baumannii*. Reversal of antibiotic resistance by efflux pump inhibitors, such as 1-(1-naphthylmethyl)-piperazine and carbonyl cyanide 3-chlorophenyl-hydrazone, supports the importance of efflux pumps in *A. baumannii* antibiotic resistance (Pannek et al., 2006; Deng et al., 2014). Four categories of efflux pumps, such as the resistance-nodulation-division superfamily, the multidrug and toxic compound extrusion family, the major facilitator superfamily, and the small multidrug resistance family transporters, are related to antimicrobial resistance in *A. baumannii* (Table 2; Lin and Lan, 2014).

AdeABC in the resistance-nodulation-division superfamily is associated with aminoglycoside resistance (Magnet et al., 2001) and with decreasing susceptibility to tigecycline (Ruzin et al., 2007) and non-fluoroquinolone antibiotics (Higgins et al., 2004). AdeABC seems to be cryptic in wild-type *A. baumannii* because of stringent control by the AdeRS two-component system (Marchand et al., 2004), but point mutations or insertion of the IS*Aba1* sequence in the *adeS* gene leads to overexpression of AdeABC (Marchand et al., 2004; Sun et al., 2012, 2016; Hammerstrom et al., 2015). Cell density (Fernando and Kumar, 2012) and the BaeSR two-component system (Lin et al., 2014, 2015), which is involved in an envelope stress response, also seem to regulate transcription of the *adeA* gene and thus affect tigecycline susceptibility. Other resistance-nodulation-division type efflux pumps, such as AdeFGH and AdeIJK, are synergistically associated with tigecycline resistance (Damier-Piolle et al., 2008). AdeFGH and AdeIJK expression is regulated by the LysR-type transcriptional regulator AdeL and the TetR-type transcriptional regulator AdeN (Coyne et al., 2010; Rosenfeld et al., 2012).

*Acinetobacter baumannii* clinical isolates possess a strong ability to form biofilms (Rodriguez-Bano et al., 2008). Notably, the subinhibitory concentrations of antibiotics encountered by low-dose therapy seem to strongly induce biofilm formation (Kaplan, 2011). A recent result revealed the mechanism. Overexpression of the AdeFGH efflux pump by low-dose antimicrobial therapy increases the synthesis and transport of autoinducer molecules, which induce biofilm formation (He X. et al., 2015). These results suggest a link between low-dose antimicrobial therapy and a high risk for biofilm infections caused by *A. baumannii*.

CmlA and CraA are major facilitator superfamily efflux pumps related with chloramphenicol (Fournier et al., 2006; Roca et al., 2009), and TetA is associated with tetracycline resistance (Ribera et al., 2003a). The novel efflux pump AmvA mediates resistance to different classes of antibiotics, disinfectants, detergents, and dyes, such as erythromycin, acriflavine, benzalkonium chloride, and methyl viologen (Rajamohan et al., 2010). AbaF was recently identified as a novel efflux pump associated with fosfomycin resistance (Sharma et al., 2016).

AbeM is in the multidrug and toxic compound extrusion family and confers resistance to imipenem and fluoroquinolones (Su et al., 2005). AbeS is the small multidrug resistance family transporter and affects resistance to various antimicrobial compounds. Deletion of the *abeS* gene results in increased susceptibility to various antimicrobial compounds, such as chloramphenicol, nalidixic acid, and erythromycin (Srinivasan et al., 2009).

Some other efflux pumps, such as MacAB-TolC (Kobayashi et al., 2001) and EmrAB-TolC (Lomovskaya and Lewis, 1992), have been well described in *E. coli*, but their role in *A. baumannii* has been recently explored. The EmrAB-TolC efflux pump is also present in *A. baumannii* where it conferred resistance to netilmicin, tobramycin, and imipenem (Nowak-Zaleska et al., 2016). Another report identified three novel efflux pumps (A1S_1535, A1S_2795, and ABAYE_0913) in *A. baumannii* using multiplexed phenotypic screening (Li L. et al., 2016). A1S_1535 confers resistance to various antibiotics, including gentamicin, kanamycin, chloroxylenol, oxytetracycline, 1,10-phenanthroline, and chloramphenicol (Li L. et al., 2016). A1S_2795 is the first major facilitator superfamily efflux pump found to confer resistance to the sulphonamide sulfathiazole, and ABAYE_0913 is associated with resistance to chloramphenicol and fusidic acid (Li L. et al., 2016).

### Permeability defects

A change in envelope permeability can influence antibiotic resistance. For example, porins form channels that allow transport of molecules across the outer membrane and play a significant role in *A. baumannii* virulence (Table 1). Because porins affect membrane permeability, they also play a significant role in the mechanism of resistance. Reduced expression of some porins, including CarO (Mussi et al., 2005, 2007; Siroy et al., 2005; Catel-Ferreira et al., 2011; Jin et al., 2011), Omp22-33 (Bou et al., 2000a), Omp33-36 (del Mar Tomas et al., 2005; Hood et al., 2010), Omp37 (Quale et al., 2003), Omp43 (Dupont et al., 2005), Omp44 (Quale et al., 2003), and Omp47 (Quale et al., 2003), is associated with carbapenem resistance in *A. baumannii*. Loss of Omp29 in *A. baumannii* producing OXA-51-like or OXA-23-like carbapenemases results in increased imipenem resistance (Jeong et al., 2009; Fonseca et al., 2013). OmpA is also related with resistance to aztreonam, chloramphenicol, and nalidixic acid (Smani et al., 2014). One study showed that OmpA and CarO physically interact with OXA-23 carbapenemase, and these interactions are associated with antibiotic resistance (Wu et al., 2016). These results provide a novel view to increase understanding of bacterial antibiotic resistance mechanisms.

Besides outer membrane proteins, envelope components, such as LPS and peptidoglycans, also affects antibiotic resistance of *A. baumannii*. Loss or modification of LPS decreases membrane integrity and increases colistin resistance in *A. baumannii* (Adams et al., 2009; Moffatt et al., 2010).

### Aminoglycoside-modifying enzymes

Aminoglycoside-modifying enzymes are the major mechanism by which *A. baumannii* confers resistance to aminoglycosides. Aminoglycoside-modifying enzymes can be classified into acetyltransferases, adenyltransferases, and phosphotransferases. These enzymes are typically present on transposable elements and are transferred among pathogenic bacteria (Lin and Lan, 2014). Several reports show that many MDR *A. baumannii* isolates produce a combination of aminoglycoside-modifying enzymes (Gallego and Towner, 2001; Nemec et al., 2004). A study from China identified a MDR *A. baumannii* strain carrying four aminoglycoside-modifying enzymes (Zhu et al., 2009). Another study from Greece reported that all *A. baumannii* strains contain aminoglycoside-modifying enzymes (Ploy et al., 1994), indicating the high prevalence of these enzymes in *A. baumannii*.

### Alteration of target sites

Modifications in antibiotic target sites for antibiotics can induce antibiotic resistance in *A. baumannii*. In the absence of other known resistance mechanisms, only overexpression of altered PBPs with a low affinity for imipenem induce imipenem resistance (Gehrlein et al., 1991). Quinolone resistance is associated with modifications in GyrA (one subunit of DNA gyrase) and ParC (one subunit of topoisomerase IV) in epidemiologically unrelated *A. baumannii* isolates (Vila et al., 1995). *Acinetobacter baumannii* TetM, which has 100% homology with *S. aureus* TetM, has been proposed to be associated with tetracycline resistance through ribosomal protection (Ribera et al., 2003b). Similar to other pathogenic bacteria, dihydrofolate reductases (DHFR and FolA) responsible for trimethoprim resistance have been found in nosocomial MDR *A. baumannii* isolates (Mak et al., 2009; Lin M. F. et al., 2013; Taitt et al., 2014). The 16S rRNA methylase ArmA responsible for aminoglycoside resistance is also found in many *A. baumannii* strains and always coexists with OXA type carbapenemases such as OXA-23 (Yu et al., 2007; Cho et al., 2009; Karthikeyan et al., 2010; Brigante et al., 2012; Hong et al., 2013; Bakour et al., 2014; Tada et al., 2014; Hasani et al., 2016). As described above, many studies have shown that modifications or/and loss of LPS decrease the susceptibility of *A. baumannii* to many clinical important antibiotics, such as colistin.

### Others

AdeABC is associated with decreased susceptibility to tigecycline (Ruzin et al., 2007). However, some clinical isolates without overexpressed AdeABC, AdeFGH, and AdeIJK have decreased susceptibility to tigecycline. Several reports have suggested the mechanism. One study analyzed eight *A. baumannii* clinical isolates and revealed that the deletion mutation in the *trm* gene, which encodes S-adenosyl-L-methionine-dependent methyltransferase, decreases susceptibility to tigecycline (Chen et al., 2014). The same result was reported using a highly efficient and versatile genome-editing platform enabling markerless modification of the *A. baumannii* genome. Deletion of AdeR, a transcription factor that regulates AdeABC efflux pump expression in tigecycline-resistant *A. baumannii*, reduces the MIC of tigecycline. However, 60% of the clinical isolates remained nonsusceptible to tigecycline after the *adeR* deletion according to a highly efficient and versatile genome-editing platform (Trebosc et al., 2016). Whole-genome sequencing in two tigecycline-resistant *adeR* deletion strains revealed that a mutation in the *trm* gene makes the *adeR* mutant resistant to tigecycline. In addition, a *trm* disruption was identified in most tigecycline-resistant clinical isolates (Trebosc et al., 2016). However, its exact mechanism was not determined. Another study revealed that a frameshift mutation in *plsC*, encoding 1-acyl-*sn*-glycerol-3-phosphate acyltransferase, is associated with decreased susceptibility to tigecycline (Li X. et al., 2015).

The *abrp* gene, which encodes the peptidase C13 family, is associated with decreased susceptibility to tetracycline, minocycline, doxycycline, tigecycline, chloramphenicol, and fosfomycin (Li X. et al., 2016). Deletion of *abrp* increases cell membrane permeability, displays slower cell growth rate, and confers reduced susceptibility to these antibiotics (Liu X. et al., 2016). However, its exact mechanism was not determined. Some genes involved in cell division, including *blhA, zipA, zapA*, and *ftsK*, are associated with intrinsic β-lactam resistance in *A. baumannii* (Knight et al., 2016).

Increased expression of mutagenesis-related genes, such as the SOS response genes, is a well-understood mechanism of *E. coli* and other bacteria to obtain antibiotic resistance (Cirz and Romesberg, 2007). *Acinetobacter baumannii* also seems to have an inducible DNA damage response in which RecA plays a major regulatory role and seems to acquire antibiotic resistances under clinically relevant DNA-damaging conditions (Aranda et al., 2011, 2014; Norton et al., 2013). Furthermore, RecA is involved in the *A. baumannii* pathogenicity (Aranda et al., 2011).

## Prospective treatment options

Although carbapenems are effective antibiotics to treat *A. baumannii* infections (Cisneros and Rodriguez-Bano, 2002; Turner et al., 2003), the rate of carbapenem-resistant *A. baumannii* isolates has been increasing gradually (Mendes et al., 2010; Kuo et al., 2012; Su et al., 2012). Only a few effective antibiotic options are available to treat MDR *A. baumannii* infections (Gordon and Wareham, 2009; Lee J. H. et al., 2015, 2016). To combat MDR or pandrug-resistant (PDR) *A. baumannii*, which are resistant to all available antibiotics, combination therapies, including colistin/imipenem, colistin/meropenem, colistin/rifampicin, colistin/tigecycline, colistin/sulbactam, colistin/teicoplanin, and imipenem/sulbactam, have been extensively studied. Prospective treatment options of *Acinetobacter baumannii* infections are summarized in Table 3. We will discuss the most recent published reports.

**Table 3:** **Prospective treatment options of *Acinetobacter baumannii* infections**.

**Drugs**	**Type of research**	**Type of *A. baumannii***	**Findings**	**References**
Carbapenem +ampicillin+sulbactam+	*In vivo*	Carbapenem-resistant	Combination therapy with ampicillin-sulbactam and meropenem is effective against skin and soft tissue infection	Hiraki et al., 2013
	*In vivo*	Multidrug-resistant	The combination of a carbapenem and ampicillin/sulbactam was associated with a better outcome than the combination of a carbapenem and amikacin, or a carbapenem alone	Kuo et al., 2007
Carbapenem +minocycline	*In vitro*	Multidrug-resistant	Minocycline in combination with rifampicin, imipenem, and colistin showed bactericidal synergy in most of the isolates which did not harbor the *tetB* gene, but the combinations were not synergistic in *tetB*-positive isolates	Rodriguez et al., 2015
Carbapenem +tigecycline+colistin	Case report	Multidrug-resistant, colistin-susceptible	A patient with bacteremia had a favorable clinical outcome by a meropenem/colistin/tigecycline combination therapy	Candel et al., 2010
Carbapenem +colistin	*In vitro*/case report	Extensively drug-resistant, colistin-susceptible	Effective; 80% of patients were treated successfully	Ozbek and Senturk, 2010
	*In vitro*	Multidrug-resistant, colistin-susceptible	Imipenem/colistin showed best synergy effects	Pongpech et al., 2010
	*In vitro*/case report	Multidrug-resistant, colistin-susceptible	Meropenem/colistin can inhibit bacterial regrowth at 24 h	Lee C. H. et al., 2008
	*In vitro*	Colistin-susceptible and colistin-resistant	Subinhibitory meropenem/colistin showed synergy against 49 of 52 strains at 24 h	Pankuch et al., 2008
	*In vitro*	Extensively drug-resistant, colistin-susceptible	Combinations of colistin/rifampicin, colistin/meropenem, colistin/minocycline and minocycline/meropenem are synergistic	Liang et al., 2011
	A retrospective study	Extensively drug-resistant, colistin-susceptible	Colistin/carbapenem and colistin/sulbactam resulted in significantly higher microbiological eradication rates, relatively higher cure and 14-day survival rates, and lower in-hospital mortality compared to colistin monotherapy in patients with bloodstream infections	Batirel et al., 2014
	*In vitro*	Carbapenem-resistant, colistin-susceptible	Synergistic effects against all 12 isolates	Liu X. et al., 2016
	*In vivo*	Extensively drug-resistant, colistin-susceptible and colistin-resistant	Colistin/fusidic acid and colistin/rifampicin were synergistic in a murine thigh-infection model; The colistin-meropenem combination was also effective when the colistin MIC is ≤32 mg/L.	Fan et al., 2016
	*In vitro*	Extensively drug-resistant	The daptomycin-colistin combination was the most effective; the colistin/imipenem combination was also effective	Cordoba et al., 2015
Carbapenem +colistin+rifampicin	Case report	Multidrug-resistant, colistin-susceptible	Successful treatment by a meropenem/colistin/rifampicin combination therapy in a case of multifocal infection	Biancofiore et al., 2007
Carbapenem+plazomicin	*In vitro*	Carbapenem-resistant	Synergistic activity	Garcia-Salguero et al., 2015
Imipenem+polymyxin B	*In vitro*	Carbapenem-resistant	Doripenem, meropenem, or imipenem displayed similar pharmacodynamics in combination with polymyxin B	Lenhard et al., 2016b
Meropenem+ polymyxin B	*In vitro*	Multidrug-resistant	Combinations of polymyxin B/meropenem and polymyxin B/meropenem/fosfomycin showed high synergistic activity	Menegucci et al., 2016
	*In vitro*/*in vivo*	Carbapenem-resistant	Intensified meropenem dosing in combination with polymyxin B synergistically killed carbapenem-resistant strains, irrespective of the meropenem MIC	Lenhard et al., 2016a
	*In vitro*	Carbapenem-resistant	Doripenem, meropenem, or imipenem displayed similar pharmacodynamics in combination with polymyxin B	Lenhard et al., 2016b
Doripenem+tigecycline	*In vitro*	Multidrug-resistant, doripenem-resistant	Synergistic activity	Principe et al., 2013
Doripenem+colistin	*In vitro*	Multidrug-resistant, doripenem-resistant	Synergistic activity	Principe et al., 2013
Doripenem+polymyxin B	*In vitro*	Carbapenem-resistant	Doripenem, meropenem, or imipenem displayed similar pharmacodynamics in combination with polymyxin B	Lenhard et al., 2016b
	*In vitro*	Polymyxin-heteroresistant	The polymyxin B/doripenem combination resulted in rapid and extensive initial killing within 24 h, which was sustained over 10 days	Rao et al., 2016a
Doripenem+amikacin	*In vitro*	Multidrug-resistant, doripenem-resistant	Synergistic activity	Principe et al., 2013
Ampicillin+sulbactam	*In vitro/in vivo*	Multi-drug resistant	Ampicillin/sulbactam therapy significantly decreased the risk of death in patients with bloodstream infections	Smolyakov et al., 2003
Sulbactam+colistin	A retrospective study	Extensively drug-resistant, colistin-susceptible	Colistin/carbapenem and colistin/sulbactam resulted in significantly higher microbiological eradication rates, relatively higher cure and 14-day survival rates, and lower in-hospital mortality compared to colistin monotherapy in patients with bloodstream infections	Batirel et al., 2014
	A retrospective study	Multidrug-resistant	The colistin/sulbactam combination therapy is promising in patients with ventilator-associated pneumonia	Kalin et al., 2014
Tazobactam+colistin	*In vivo*	Colistin-susceptible	Tazobactam plus colistin showed synergy	Sakoulas et al., 2016
Minocycline+colistin	*In vitro*	Extensively drug-resistant	Combinations of colistin/rifampicin, colistin/meropenem, colistin/minocycline and minocycline/meropenem are synergistic	Liang et al., 2011
	*In vitro*/*in vivo*	Minocycline-resistant	Minocycline/colistin synergistically killed minocycline-resistant isolates; minocycline/colistin also significantly improved the survival of mice and reduced the number of bacteria present in the lungs of mice	Yang et al., 2016
	*In vitro*	Multidrug-resistant	Minocycline in combination with rifampicin, imipenem, and colistin showed bactericidal synergy in most of the isolates which did not harbor the *tetB* gene, but the combinations were not synergistic in *tetB*-positive isolates	Rodriguez et al., 2015
Minocycline+rifampicin	*In vivo*	Multidrug-resistant	Synergistic effect of minocycline/rifampicin and minocycline/amikacin combinations in a mouse lung infection model	He S. et al., 2015
	*In vitro*	Multidrug-resistant	Minocycline in combination with rifampicin, imipenem, and colistin showed bactericidal synergy in most of the isolates which did not harbor the *tetB* gene, but the combinations were not synergistic in *tetB*-positive isolates	Rodriguez et al., 2015
Minocycline+amikacin	*In vivo*	Multidrug-resistant	Synergistic effect of minocycline/rifampicin and minocycline/amikacin combinations in a mouse lung infection model	He S. et al., 2015
Tigecycline+colistin	*In vitro*	Carbapenem-resistant, colistin-susceptible	Good synergy	Ozbek and Senturk, 2010; Sheng et al., 2011
	*In vitro*	Extensively drug-resistant, colistin-susceptible	Good synergy	Dizbay et al., 2010
	*In vitro*	Tigecycline-non-susceptible	Good synergy	Principe et al., 2009
	*In vitro*	Carbapenem-resistant, colistin-susceptible and colistin-resistant	Good synergy	Peck et al., 2012
	*In vitro*/*in vivo*	Extensively drug-resistant	*In vitro* synergistic activity; no statistically significant differences were found between colistin, tigecycline, and combination treatments in terms of efficacy on bacterial counts in lung tissue of a rat pneumonia model	Mutlu Yilmaz et al., 2012
Tigecycline+polymyxin B	*In vitro*	Carbapenem-resistant, polymyxin-heteroresistant	Combination of polymyxin B-with higher tigecycline concentrations result in sustained bactericidal activity	Rao et al., 2016b
	*In vitro*	Carbapenem-resistant	Synergistic effects in combination therapy with simulated exposures of polymyxin B and tigecycline at an aggressive dose	Hagihara et al., 2014
Tigecycline+amikacin	*In vitro*	Multidrug-resistant	Synergistic bactericidal activities	Moland et al., 2008
Colistin+rifampicin	*In vitro*/*in vivo*	Multidrug-resistant, colistin-susceptible	Efficacy *in vitro* and in experimental models of pneumonia and meningitis	Pachon-Ibanez et al., 2010
	Case report	Carbapenem-resistant, colistin-susceptible	Efficacy in 7 of 10 patients with ventilator-associated pneumonia	Song et al., 2008
	Case report	Multidrug-resistant, colistin-susceptible	Efficacy in 22 of 29 critically ill patients with pneumonia and bacteremia	Bassetti et al., 2008
	*In vivo*	Multidrug-resistant, colistin-susceptible	Synergistic effect in prolonging survival	Pantopoulou et al., 2007
	Clinical trial	Multidrug-resistant, colistin-susceptible	Favorable for all 26 nosocomial infection patients	Motaouakkil et al., 2006
	*In vitro*	Carbapenem-resistant, colistin-susceptible	Effective for strains highly resistant to imipenem and moderately resistant to rifampicin	Montero et al., 2004
	*In vitro*	Multidrug-resistant, colistin-susceptible	Synergistic effect against 11 of 13 isolates	Hogg et al., 1998
	*In vitro*	Extensively drug-resistant	Combinations of colistin/rifampicin, colistin/meropenem, colistin/minocycline and minocycline/meropenem are synergistic	Liang et al., 2011
	*In vitro*	Multidrug-resistant, colistin-susceptible	Colistin/rifampicin was fully synergistic against 4 of 5 isolates; colistin/meropenem and colistin/azithromycin were synergistic against 3 of 5 isolates; colistin/doxycycline was partially synergistic or additive against 5 isolates	Timurkaynak et al., 2006
	Case report	Carbapenem-resistant, colistin-susceptible	Rifampicin/colistin and ampicillin/sulbactam resulted in microbiological clearance in 9 of 14 critically ill patients	Petrosillo et al., 2005
	*In vitro*	Carbapenem-resistant, colistin-heteroresistant	Rifampicin/colistin and imipenem/colistin were synergistic against heteroresistant isolates and prevented the development of colistin-resistant strains	Rodriguez et al., 2010
	Case report	Carbapenem-resistant, colistin-susceptible	Synergistic effect in patients with ventilator-associated pneumonia	Aydemir et al., 2013
	*In vivo*	Extensively drug-resistant, colistin-susceptible and colistin-resistant	Colistin/fusidic acid and colistin/rifampicin were synergistic in a murine thigh-infection model; The colistin-meropenem combination was also effective when the colistin MIC is ≤32 mg/L.	Fan et al., 2016
	*In vitro*	Colistin-resistant	The most effective combinations were colistin-rifampin and colistin-teicoplanin; both combinations showed synergistic effect against 8 of 9 colistin-resistant strains	Bae et al., 2016
Colistin+teicoplanin	*In vitro*/*in vivo*	Multidrug-resistant, colistin-susceptible	Synergistic effect of colistin/daptomycin and colistin/teicoplanin in a mouse model	Cirioni et al., 2016
	*In vitro*	Multidrug-resistant, colistin-susceptible	Significant synergy	Wareham et al., 2011
	*In vitro*	Colistin-resistant	The most effective combinations were colistin-rifampin and colistin-teicoplanin; both combinations showed synergistic effect against 8 of 9 colistin-resistant strains	Bae et al., 2016
Colistin+daptomycin	*In vitro*/*in vivo*	Multidrug-resistant, colistin-susceptible	Synergistic effect of colistin/daptomycin and colistin/teicoplanin in a mouse model	Cirioni et al., 2016
	*In vitro*	Extensively drug-resistant	The daptomycin-colistin combination was the most effective; the colistin/imipenem combination was also effective	Cordoba et al., 2015
Colistin+vancomycin	*In vitro*/*in vivo*	Multidrug-resistant, colistin-susceptible	Highly active both in vitro and in an animal model of *Galleria mellonella*	Hornsey and Wareham, 2011
Colistin+fosfomycin	*In vitro*	Carbapenem-resistant, colistin-susceptible	Good synergy; no synergy between colistin and sulbactam, colistin and imipenem	Santimaleeworagun et al., 2011
Colistin+fusidic acid	*In vitro*	Carbapenem-resistant, colistin-susceptible and colistin-resistant	*In vitro* synergy between colistin and fusidic acid that is comparable to the synergy between colistin and vancomycin; the synergy with fusidic acid is strain-dependent and applicable to strains for which the colistin MICs are relatively low	Bowler et al., 2016
	*In vitro*	Carbapenem-resistant, colistin-susceptible and colistin-resistant	Robust synergy between fusidic acid and colistin against multidrug-resistant clinical strains, including some colistin-resistant strains	Phee et al., 2015
	*In vivo*	Extensively drug-resistant, colistin-susceptible and colistin-resistant	Colistin/fusidic acid and colistin/rifampicin were synergistic in a murine thigh-infection model; The colistin-meropenem combination was also effective when the colistin MIC is ≤32 mg/L.	Fan et al., 2016
Colistin+amikacin	Case report	Multidrug-resistant, colistin-susceptible	Successful clinical and microbiological outcomes	Fulnecky et al., 2005
Colistin+trimethoprim-sulfamethoxazole	*In vitro*	Carbapenem-resistant	Colistin/trimethoprim-sulfamethoxazole killed effectively all carbapenem-resistant strains	Nepka et al., 2016
Polymyxin B+netropsin	*In vitro*/*in vivo*	Colistin-resistant	The survival of infected *Galleria mellonella* was significantly higher when treated with polymyxin B and netropsin in combination than when treated with polymyxin B or netropsin alone	Chung et al., 2016
Trimethoprim-sulfamethoxazole	*In vitro*	Carbapenem-resistant	Trimethoprim-sulfamethoxazole killed effectively all carbapenem-resistant strains	Nepka et al., 2016
Novobiocin	*In vitro*	Carbapenem-susceptible	Inhibition of frequency of the occurrence of rifampin resistance mutants	Jara et al., 2015
Bacteriophages	*In vitro*/*in vivo*	Carbapenem-resistant, carbapenem-susceptible	Strong lytic activities and the improvement of survival rates	Jeon et al., 2016; Kusradze et al., 2016
Endolysin (LysABP-01)+colistin	*In vitro*	Multidrug-resistant	Synergistic activity	Thummeepak et al., 2016
Artilysins	*In vitro*	Carbapenem-resistant, carbapenem-susceptible	Artilysins are effective *in vitro* and *in vivo*	Briers et al., 2014; Yang et al., 2015; Defraine et al., 2016; Thandar et al., 2016
Antimicrobial peptides	*In vitro*	Multidrug-resistant	Good antimicrobial activities	Pires et al., 2015; Barksdale et al., 2016
Rose bengal+ carbapenem	*In vitro*	Carbapenem-resistant	Imipenem or meropenem with rose bengal showed synergistic effects	Chiu et al., 2016
β-Aminoketone (MD3)+colistin	*In vitro*	Colistin-susceptible, colistin-resistant	Synergistic effect targeting to strains with specific colistin resistance mechanisms; synergy against both colistin-susceptible strains and colistin-resistant strains with mutations in *pmrB* and phosphoethanolamine modification of lipid A, but not against colistin-resistant strains with loss of lipopolysaccharide	Martinez-Guitian et al., 2016
Bulgecin A+ carbapenem	*In vitro*	Carbapenem-resistant	Synergistic activity	Skalweit and Li, 2016
Farnesol+colistin	*In vitro*	Colistin-resistant	Farnesol increased sensitivity to colistin for colistin-resistant strains	Kostoulias et al., 2015
Oleanolic acid+gentamicin or kanamycin	*In vitro*	Carbapenem-susceptible	Synergistic activity	Shin and Park, 2015
Cyanide 3-chlorophenylhydrazone (CCCP)+colistin	*In vitro*	Colistin-resistant	CCCP reversed colistin resistance and inhibited the regrowth of the resistant subpopulation	Ni et al., 2016
	*In vitro*	Colistin-resistant	Synergistic activity	Park and Ko, 2015
ABEPI1 or ABEPI2+minocycline	*In vitro*	Carbapenem-susceptible	Synergistic activity	Blanchard et al., 2014
Gallium nitrate	*In vitro/in vivo*	Multidrug-resistant	Good antimicrobial activities; protection of *Galleria mellonella* larvae from lethal *A. baumannii* infection; synergistic activity with colistin	Antunes et al., 2012
Gallium protoporphyrin IX	*In vitro/in vivo*	Multidrug-resistant	Good antimicrobial activities	Arivett et al., 2015
Gallium nitrate+colistin	*In vitro*/*in vivo*	Multidrug-resistant	Good antimicrobial activities; protection of *Galleria mellonella* larvae from lethal *A. baumannii* infection; synergistic activity with colistin	Antunes et al., 2012
D-amino acids	*In vitro*/*in vivo*	Carbapenem-susceptible	Some D-amino acids (D-histidine and D-cysteine) can inhibit bacterial growth, biofilm formation and adherence to eukaryotic cells	Rumbo et al., 2016
*Bifidobacterium breve* strain Yakult	*In vivo*	Multidrug-resistant	Protection against fatal intestinal infection in a murine infection model	Asahara et al., 2016
Clarithromycin	*In vivo*	Multidrug-resistant	Inhibition of bacterial growth and biofilm formation; immunomodulator	Konstantinidis et al., 2016
Lysophosphatidylcholine+carbapenem	*In vivo*	Multidrug-resistant strain	Lysophosphatidylcholine in combination with colistin, tigecycline, or imipenem markedly enhanced the bacterial clearance from the spleen and lungs and reduced bacteremia and mouse mortality rates	Parra Millan et al., 2016
Lysophosphatidylcholine+tigecycline	*In vivo*	Multidrug-resistant strain	Lysophosphatidylcholine in combination with colistin, tigecycline, or imipenem markedly enhanced the bacterial clearance from the spleen and lungs and reduced bacteremia and mouse mortality rates	Parra Millan et al., 2016
Lysophosphatidylcholine+colistin	*In vivo*	Multidrug-resistant strain	Lysophosphatidylcholine in combination with colistin, tigecycline, or imipenem markedly enhanced the bacterial clearance from the spleen and lungs and reduced bacteremia and mouse mortality rates	Parra Millan et al., 2016

### Carbapenems and β-lactamase inhibitors

Carbapenems, including imipenem, meropenem, and doripenem, have generally been considered the agents to treat *A. baumannii* infections, due to their effective activity against this organism and their favorable safety (Doi et al., 2015). However, the decreased susceptibility of *A. baumannii* to carbapenems has forced clinicians and researchers to explore alternative therapeutic approaches (Doi et al., 2015). Because carbapenem-resistant *A. baumannii* strains are often resistant to all other commonly used antibiotics as well, these strains remain susceptible to only limited antibiotics, such as minocycline/tigecycline and polymyxins (colistin and polymyxin B; Lin and Lan, 2014; Doi et al., 2015). Carbapenem therapies combined with a few effective antibiotics was extensively tested and many cases showed a synergistic effect against *A. baumannii* infections (Table 3). However, recent increase of tigecycline- or colistin-resistant *A. baumannii* increasingly poses a serious threat to public health worldwide (Peleg et al., 2007; Hornsey et al., 2010; Cai et al., 2012).

Sulbactam is a β-lactamase inhibitor and also has affinity for penicillin-binding proteins of *A. baumannii* (Rafailidis et al., 2007; Doi et al., 2015). Combined therapy of ampicillin with sulbactam is effective for treating bloodstream infections due to MDR *A. baumannii* (Smolyakov et al., 2003). Ampicillin/sulbactam/carbapenem combination therapy is also effective for treating MDR *A. baumannii* bacteremia (Kuo et al., 2007) and skin and soft tissue infection of carbapenem-resistant *A. baumannii* (Hiraki et al., 2013), but not in ventilator-associated pneumonia (Kalin et al., 2014). The population pharmacokinetics and pharmacodynamics of sulbactam were determined in critically ill patients with severe sepsis caused by *A. baumannii* (Jaruratanasirikul et al., 2016) and in patients with impaired renal function (Yokoyama et al., 2015). Another β-lactamase inhibitor tazobactam increases the activity of peptide antibiotics, such as colistin and daptomycin, in a murine model of *A. baumannii* pneumonia (Sakoulas et al., 2016). The authors suggested that β-lactamase inhibitors may exert similar effects in potentiating peptide antibiotics, because of structural similarities between β-lactamase inhibitors and peptide antibiotics (Sakoulas et al., 2016).

### Minocycline/tigecycline

Minocycline, is a broad-spectrum tetracycline antibiotic that has been proposed for treating drug-resistant *A. baumannii* based on its high degree of susceptibility to this drug and its favorable pharmacokinetic profile (Ritchie and Garavaglia-Wilson, 2014). The mean susceptibility rate of *A. baumannii* to minocycline is approximately 80% worldwide (Castanheira et al., 2014). Therefore, minocycline therapy has high treatment success rates and good tolerability (Ritchie and Garavaglia-Wilson, 2014). However, since the introduction of minocycline, approximately 20% of *A. baumannii* isolates are not susceptible to minocycline. The TetB efflux pump is the main determinants of minocycline resistance (Vilacoba et al., 2013). Minocycline therapy combined with colistin is effective for treating minocycline-resistant *A. baumannii* infections (Yang et al., 2016), and minocycline therapy combined with rifampicin, colistin, or imipenem has a synergistic effect in most of isolates without the *tetB* gene, but combined therapies are not synergistic in isolates with the *tetB* gene (Rodriguez et al., 2015).

Tigecycline is the first glycylcycline class antibiotic that exhibits bacteriostatic activity by binding to the 30S ribosomal subunit, and is active against *A. baumannii* infections (Pachon-Ibanez et al., 2004; Anthony et al., 2008; Koomanachai et al., 2009). Tigecycline shows a synergistic effect with some classes of antibiotics, such as amikacin (Moland et al., 2008) and colistin (Mutlu Yilmaz et al., 2012). However, limitations of tigecycline use have emerged with its increasing use. Tigecycline is less effective than imipenem to treat pneumonia in a murine pneumonia model (Pichardo et al., 2010). β-Lactam or carbapenem instead of tigecycline was recommended for *A. baumannii* infections with tigecycline MIC of more than 2 mg/L, due to high mortality from the tigecycline treatment (Curcio and Fernandez, 2008). In a study of 266 patients with MDR *A. baumannii* infections, tigecycline-based therapy was not more effective than non-tigecycline-based therapies (Lee Y. T. et al., 2013). Tigecycline resistance associated with overexpression of efflux pumps, such as AdeABC, has been reported in clinical isolates of *A. baumannii* (Peleg et al., 2007; Ruzin et al., 2007; Hornsey et al., 2010, 2011). Multiple MDR *A baumannii* clones resistant to tigecycline have been reported in many medical centers (Navon-Venezia et al., 2007). Therefore, tigecycline can only be used in limited cases for treating *A. baumannii* infections.

### Polymyxins (colistin and polymyxin B)

Polymyxins are a group of polycationic peptide antibiotics that were discovered more than 60 years ago and exhibit potent efficacy against most Gram-negative bacteria (Liu Q. et al., 2014; Lee C. R. et al., 2016). Among all five polymyxins (A–E), only polymyxin B and E (colistin) with a one amino acid difference are used clinically. Colistin is a key component of combination therapies used to treat MDR *A. baumannii* infections (Cai et al., 2012). The rate of colistin resistance (10.4%) in MDR *A. baumannii* isolates is lower than that of rifampicin (47.8%) or tigecycline (45.5%) resistance (Chang et al., 2012). Similar results were reported in another study (Muthusamy et al., 2016). Therefore, colistin seems to be the only effective antimicrobial agent against MDR *A. baumannii* infections. Many colistin-based combined therapies, including colistin/rifampicin (Liang et al., 2011; Aydemir et al., 2013), colistin/minocycline (Liang et al., 2011), colistin/carbapenem (Liang et al., 2011; Batirel et al., 2014; Liu X. et al., 2016), colistin/sulbactam (Batirel et al., 2014), colistin/tigecycline (Principe et al., 2009; Ozbek and Senturk, 2010; Sheng et al., 2011; Peck et al., 2012), colistin/daptomycin (Cirioni et al., 2016), colistin/fusidic acid (Bowler et al., 2016; Fan et al., 2016), and colistin/teicoplanin (Wareham et al., 2011; Cirioni et al., 2016), are synergistic *in vivo* or *in vitro* against *A. baumannii* infections. Colistin therapy combined with rifampin or fusidic acid seems to be the most effective for treating a MDR *A. baumannii* in a murine thigh-infection model (Fan et al., 2016). Another report comparing colistin/daptomycin, colistin/imipenem, and imipenem/ertapenem showed that the daptomycin-colistin combination was the most effective (Cordoba et al., 2015).

Unfortunately, the emergence of colistin-resistant *A. baumannii* strains has increased worldwide (Cai et al., 2012). The mechanisms of colistin resistance include loss of LPS (Moffatt et al., 2010) and the addition of phosphoethanolamine to LPS by the PmrAB two-component system (Adams et al., 2009). Mutations in *pmrA* and *pmrB* activate *pmrC*, which adds phosphoethanolamine to the hepta-acylated form of lipid A (Beceiro et al., 2011). Interestingly, an investigation of the *in vitro* activities of various antimicrobial combinations against colistin-resistant *A. baumannii* showed that the most effective combinations against colistin-resistant *A. baumannii* are colistin-rifampin and colistin-teicoplanin, indicating that colistin is the most common constituent of antimicrobial combinations even against colistin-resistant *A. baumannii* (Bae et al., 2016). Similarly, minocycline therapy in combination with colistin is effective to treat infections caused by minocycline-resistant *A. baumannii*. Minocycline/colistin therapy significantly improves survival of mice infected with minocycline-resistant *A. baumannii* and reduces the number of bacteria present in the lungs of mice (Yang et al., 2016).

A urinary tract *Enterococcus faecalis* isolate that apparently requires vancomycin to grow was reported in 1994, and this phenomenon is called “antimicrobial agent dependence.” Colistin dependence was reported in an *A. baumannii*–*A. calcoaceticus* complex (Hawley et al., 2007). Partial colistin dependence has been detected in several LPS-deficient strains with mutations in *lpxA, lpxC*, and *lpxD* (Garcia-Quintanilla et al., 2015). Many colistin-susceptible *A. baumannii* isolates develop colistin dependence *in vitro* after exposure to colistin (Hong et al., 2016). Although the clinical implication of colistin dependence and its molecular mechanism remain unclear, it is interesting that patients with colistin-dependent *A. baumannii* isolates show a high rate of treatment failure (Hong et al., 2016).

Unlike colistin, polymyxin B is not converted from a prodrug form into an active form; thus, plasma concentrations of polymyxin B more quickly reach target levels (Sandri et al., 2013). In addition, polymyxin B is available for direct parenteral administration (Zavascki et al., 2007). Despite the favorable pharmacokinetics of polymyxin B, dose-related nephrotoxicity limits the concentration of polymyxin B used in combination therapy (Dubrovskaya et al., 2015). Therefore, almost all studies on polymyxins are carried out for colistin. However, because some carbapenems have comparatively safer dose modulation to optimize killing during combination therapy (Cannon et al., 2014), several studies have analyzing the pharmacodynamics of carbapenems in combination with polymyxin B (Lenhard et al., 2016a,b; Rao et al., 2016a). One study showed that intensified meropenem dosing combined with polymyxin B is a good strategy to treat carbapenem-resistant *A. baumannii*, regardless of the meropenem MIC (Lenhard et al., 2016a). Combination therapy with doripenem and polymyxin B also showed similar results. Early aggressive dosing of doripenem combined with polymyxin B is effective for treating heteroresistant *A. baumannii* infections (Rao et al., 2016a). A combined pharmacodynamics analysis of four different carbapenems with polymyxin B showed that doripenem, meropenem, or imipenem display similar pharmacodynamics in combination, and the decision to use carbapenem in combination with polymyxin B is usually based on toxicodynamic profiles (Lenhard et al., 2016b). Polymyxin B also shows good bactericidal activity in combination with high tigecycline concentrations (Hagihara et al., 2014; Rao et al., 2016b). Therefore, polymyxin B combination therapies seem to be one of the most promising options for minimizing the emergence of polymyxin resistance. Increasing the dose intensity of polymyxin B amplifies polymyxin B resistance in *A. baumannii* (Cheah et al., 2016; Tsuji et al., 2016). In conclusion, although polymyxin B displays dose-related nephrotoxicity, it is a potential therapeutic alternative to colistin when use together with intensified doses of other antibiotics. Large-scale screening of *Streptomyces* secondary metabolites was performed to develop a novel combination therapy using minimal concentrations of polymyxin B, and the reliable polymyxin synergist netropsin was identified (Chung et al., 2016). Survival of *G. mellonella* infected with colistin-resistant clinical *A. baumannii* isolates is significantly higher when treated with polymyxin B combined with netropsin than when treated with polymyxin B or netropsin alone (Chung et al., 2016).

### Other antibiotics

Trimethoprim-sulfamethoxazole is a two antibiotics combination that exerts a synergistic effect by inhibiting successive steps in the folate synthesis pathway against a number of bacteria (Wormser et al., 1982). The *in vitro* killing activity of trimethoprim-sulfamethoxazole against carbapenem-resistant *A. baumannii* was recently studied. Trimethoprim-sulfamethoxazole alone effectively kills all carbapenem-resistant *A. baumannii* strains and trimethoprim-sulfamethoxazole combined with colistin also rapidly kills all strains for up to 24 h (Nepka et al., 2016). These results suggest that trimethoprim-sulfamethoxazole might be an effective therapy for severe carbapenem-resistant *A. baumannii* infections. Plazomicin is a next-generation aminoglycoside synthetically derived from sisomicin that enhances activity against many MDR Gram-negative bacteria (Garcia-Salguero et al., 2015). A synergistic effect was observed with carbapenems along with plazomicin during treatment of *A. baumannii* infections (Garcia-Salguero et al., 2015), indicating the potential utility of plazomicin combined with carbapenems.

The inducible DNA damage response in *A. baumannii* plays an important role in acquiring antibiotic resistance under clinically relevant DNA-damaging conditions (Aranda et al., 2011, 2014; Norton et al., 2013). The aminocoumarin novobiocin is a well-established antimicrobial agent that inhibits the DNA damage response in Gram-positive bacteria by interfering with ATPase activity of DNA gyrase (Schroder et al., 2013). One study showed that novobiocin also inhibits acquisition of antimicrobial resistance in MDR *A. baumannii* through DNA damage-induced mutagenesis (Jara et al., 2015).

### Non-antibiotic therapies: phage and others

The worldwide spread of MDR pathogens has renewed interest in the therapy using bacteriophage, which is a virus that infects and lyses bacteria. Various lytic *A. baumannii* bacteriophages, such as *vB_Ab-M-G7* (Kusradze et al., 2016) and Bϕ-C62 (Jeon et al., 2016), have been used to treat infections caused by MDR *A. baumannii*. Bacteriophage-encoded endolysin has also received attention. Endolysin is a lytic enzyme that degrades the cell wall of bacterial hosts and shows promise as a novel class of antibacterials with a unique mode of action (Defraine et al., 2016). For example, endolysin from *A. baumannii* bacteriophage ØABP-01 degrades the crude cell wall of *A. baumannii* strains and elevates antibacterial activity when combined with colistin (Thummeepak et al., 2016). However, most Gram-negative pathogens are generally not susceptible to endolysins, due to their protective outer membrane (Lee et al., 2013a). To overcome this problem, endolysins have recently been engineered with specific outer membrane-destabilizing peptides to obtain the ability to penetrate outer membrane and these engineered endolysins are called “artilysins” (Rodriguez-Rubio et al., 2016). Several engineered artilysins have been developed to combat MDR *A. baumannii* and show highly effective antimicrobial activity against *A. baumannii* (Briers et al., 2014; Yang et al., 2015; Defraine et al., 2016; Thandar et al., 2016). These results suggest that artilysins can be a treatment option for MDR *A. baumannii*. The diversity of the phage population was determined by analysis of viromes, endolysins, and CRISPR spacers (Davison et al., 2016). These results can be used to assist in finding an effective endolysin for combating MDR *A. baumannii*. Various peptides, such as American alligator plasma peptide (Barksdale et al., 2016) and antimicrobial peptide dendrimer G3KL (Pires et al., 2015), have *in vitro* antimicrobial activity against MDR *A. baumannii*. However, the use of antimicrobial enzymes or peptides also has some important problems, such as their short half-life in serum and high production costs compared with those of smaller molecules.

An *in silico* analysis predicted that OXA-58, OXA-23, and OXA-83 are translocated to the periplasm via the Sec system (Liao et al., 2015; Chiu et al., 2016). A SecA inhibitor (rose bengal) inhibits periplasmic translocation of these carbapenem-hydrolyzing class D β-lactamases, indicating that these β-lactamases are selectively released via a Sec-dependent system (Liao et al., 2015; Chiu et al., 2016). Imipenem or meropenem combined with rose bengal shows synergistic effects for carbapenem-resistant *A. baumannii* clinical isolates (Chiu et al., 2016). Similarly, β-aminoketone (MD3), an inhibitor of bacterial type I signal peptidases that cleaves the amino-terminal signal peptides of translocated proteins, shows a synergistic effect when combined with colistin against colistin-resistant *A. baumannii* strains (Martinez-Guitian et al., 2016).

Bulgecin A is a natural product of *P. mesoacidophila* and a lytic transglycosylase inhibitor that works synergistically with β-lactams (Skalweit and Li, 2016). Bulgecin A restores the efficacy of meropenem in suppressing growth of carbapenem-resistant *A. baumannii* strains, suggesting that Bulgecin A may be an adjunctive compound to extend the life of carbapenems against *A. baumannii* infections (Skalweit and Li, 2016). Similarly, farnesol, a natural product of *Candida albicans* for quorum-sensing, disrupts *A. baumannii* cell membrane integrity, alters cell morphology, and increases sensitivity of MDR *A. baumannii* strains to colistin (Kostoulias et al., 2015). Many herbal active compounds have potent antibacterial activities against many bacteria including carbapenem-resistant *A. baumannii* (Lin et al., 2015). For example, oleanolic acid is a triterpenoid compound that widely exists in food, medicinal herbs, and many plants and can potently inhibit various pathogenic bacteria. One study showed that oleanolic acid increases aminoglycoside uptake by changing membrane permeability and energy metabolism in *A. baumannii* (Shin and Park, 2015).

Cyanide 3-chlorophenylhydrazone (CCCP) is an efflux pump inhibitor that decreases the MIC of colistin in colistin-susceptible and colistin-resistant *A. baumannii* strains (Park and Ko, 2015; Ni et al., 2016). Other efflux pump inhibitors, such as ABEPI1 and ABEPI2, inhibit efflux-mediated minocycline tolerance of *A. baumannii*. Adding these compounds during growth in human serum leads to the accumulation of minocycline within *A. baumannii* and inhibits efflux potential of the bacterium (Blanchard et al., 2014).

Gallium is a semi-metallic element in group 13 of the periodic table that binds to biological complexes containing Fe^3+^ and disrupts essential redox-driven biological processes (Bernstein, 1998). Gallium has been used as a simple inorganic or organic salt or complexed with organic compounds. Several studies have shown that gallium nitrate or gallium protoporphyrin IX could be a viable therapeutic option for treating MDR *A. baumannii* (Antunes et al., 2012; Arivett et al., 2015). Some d-amino acids, such as d-His and d-Cys, inhibit bacterial growth, biofilm formation, and adherence to eukaryotic cells in *A. baumannii* (Rumbo et al., 2016).

Probiotics are “live microorganisms that confer a health benefit on the host when administered in adequate amounts” (Reid et al., 2005) and assist in protecting against MDR *A. baumannii* infections. For example, the ability of the probiotic *Bifidobacterium breve* to protect against MDR *A. baumannii* infections has been investigated (Asahara et al., 2016). This probiotic markedly potentiates protection against fatal intestinal infections caused by MDR *A. baumannii* (Asahara et al., 2016). With probiotics, immunomodulators, such as lysophosphatidylcholine (Parra Millan et al., 2016) and macrolide antibiotics such as clarithromycin (Konstantinidis et al., 2016), can reduce *A. baumannii* infection severity by stimulating the immune response, when combined with antibiotics such as colistin, tigecycline, or imipenem.

## Conclusion

The number of studies about *A. baumannii* is increasing dramatically because of its increasing clinical importance. Use of animal models has produced important data regarding virulence factors that contribute to *A. baumannii* pathogenesis. Notably, some studies on metal acquisition and protein secretion systems are interesting. Besides iron acquisition systems such as acinetobactin, the discovery of zinc and manganese acquisition systems in *A. baumannii* broadens our understanding of *A. baumannii* pathogenesis. More extensive studies on various protein secretion systems present in *A. baumannii* are required. About 300 genes required for *in vivo* survival of *A. baumannii* were identified using transposon screening in *G. mellonella* larvae (Gebhardt et al., 2015). Because many of these genes were not known to be associated with *A. baumannii* pathogenesis, more detailed studies are required to determine whether these genes are related to the pathogenesis of *A. baumannii*. In addition, transposon screening in other model animals will provide novel insight into *A. baumannii* pathogenesis. Knowledge of virulence factors responsible for *A. baumannii* pathogenicity will be the cornerstone for developing novel antibiotics. For example, LPS is an important virulence factor and LpxC inhibitor, which inhibits LPS synthesis, completely protects mice from lethal infection (Lin et al., 2012). These results indicate that blocking LPS synthesis is a powerful strategy for discovering novel antibiotics. However, despite recent extensive studies about *A. baumannii* pathogenesis, the toxicity and pathogenicity of *A. baumannii* remain unclear.

Recent interest about *A. baumannii* is mostly due to its seemingly endless capacity to acquire antibiotic resistance. *A. baumannii* has almost all bacterial resistance mechanisms. All class β-lactamases have been detected in *A. baumannii* and the frequency of carbapenem-resistant *A. baumannii* isolates is very high. Furthermore, almost all *A. baumannii* contain aminoglycoside-modifying enzymes and many efflux pumps responsible for resistance to various clinically important antibiotics have been identified in *A. baumannii*. Due to these abilities, available antibiotics to treat *A. baumannii* infections are significantly limited. Colistin is used as the antibiotic treatment of last resort, due to its relatively low resistance rate. However, emergence of colistin-resistant *A. baumannii* strains has increased worldwide with increasing use of colistin. Notably, some more recent studies have proposed that another polymyxin antibiotic, polymyxin B, is a potential therapeutic alternative to colistin (Lenhard et al., 2016a,b; Rao et al., 2016a; Repizo et al., 2015). Polymyxin B has not been a good antibiotic owing to dose-dependent nephrotoxicity, but recent reports show that a novel combination therapy with carbapenems or tigecycline using minimal concentrations of polymyxin B can be a good strategy to treat carbapenem-resistant *A. baumannii* infections. These results indicate the requirement for extensive studies that analyze the pharmacodynamics of polymyxin B in combination therapy.

Various trials to identify a novel alternative to carbapenem or colistin have been performed. Among them, engineered endolysins (artilysins) are particularly interesting, despite evident defects. A lytic enzyme degrading peptidoglycan of bacteria is a promising novel class of antimicrobial agents due to its unique mode of action. Similar to β-lactam antibiotics that are one of the most successful antibiotics, inhibition of peptidoglycan synthesis is a promising target of antimicrobial agents. Because lytic enzymes directly degrade peptidoglycans, but not proteins, the possibility of the emergence of a resistance mechanism is relatively low. In addition, enzymes with relatively high molecular weight are not inhibited by efflux pumps. If the short stability of artilysin in serum and high cost in its production compared with small molecules can be resolved, the improved artilysin can be a good treatment option for carbapenem- or colistin-resistant *A. baumannii* infections. In conclusion, novel, rationally designed strategies and screening-based approaches are required to discover new classes of antibiotics. If we continue to take all efforts at maintaining the effectiveness of antibiotics and developing novel antibiotics, effective control of *A. baumannii* infections can be successful.

## Author contributions

CL, JL, MP, and SL contributed to the conception and the design of the review and CL, JL, MP, KP, IB, YK, CC, BJ, and SL researched and wrote the review.

## Funding

This review was supported by the Cooperative Research Program for Agriculture Science and Technology Development (No. PJ01103103) of Rural Development Administration in Republic of Korea; the Environmental Health Action Program (No. 2016001350004) funded by the Ministry of Environment (MOE) in Republic of Korea; and the National Research Foundation of the Ministry of Education, Republic of Korea (NRF-2015R1C1A1A02037470).

### Conflict of interest statement

The authors declare that the research was conducted in the absence of any commercial or financial relationships that could be construed as a potential conflict of interest.

## References

[B1] Acosta J. Merino M. Viedma E. Poza M. Sanz F. Otero J. R.. (2011). Multidrug-resistant *Acinetobacter baumannii* Harboring OXA-24 carbapenemase, Spain. Emerg. Infect Dis. 17, 1064–1067. 10.3201/eid/1706.091866. 21749771 PMC3358182

[B2] Adams M. D. Goglin K. Molyneaux N. Hujer K. M. Lavender H. Jamison J. J.. (2008). Comparative genome sequence analysis of multidrug-resistant *Acinetobacter baumannii*. J. Bacteriol. 190, 8053–8064. 10.1128/JB.00834-08 18931120 PMC2593238

[B3] Adams M. D. Nickel G. C. Bajaksouzian S. Lavender H. Murthy A. R. Jacobs M. R.. (2009). Resistance to colistin in *Acinetobacter baumannii* associated with mutations in the PmrAB two-component system. Antimicrob. Agents Chemother. 53, 3628–3634. 10.1128/AAC.00284-09 19528270 PMC2737849

[B4] Afzal-Shah M. Woodford N. Livermore D. M. (2001). Characterization of OXA-25, OXA-26, and OXA-27, molecular class D β-lactamases associated with carbapenem resistance in clinical isolates of *Acinetobacter baumannii*. Antimicrob. Agents Chemother. 45, 583–588. [Epub ahead of print]. 10.1128/AAC.45.2.583-588.2001 11158758 PMC90330

[B5] Al-Agamy M. H. Jeannot K. El-Mahdy T. S. Shibl A. M. Kattan W. Plesiat P.. (2016). First Detection of GES-5 Carbapenemase-Producing *Acinetobacter baumannii* Isolate. Microb. Drug Resist. [Epub ahead of print]. 10.1089/mdr.2016.0152 27854148

[B6] Al Atrouni A. Hamze M. Jisr T. Lemarie C. Eveillard M. Joly-Guillou M. L.. (2016). Wide spread of OXA-23-producing carbapenem-resistant *Acinetobacter baumannii* belonging to clonal complex II in different hospitals in Lebanon. Int. J. Infect. Dis. 52, 29–36. 10.1016/j.ijid.2016.09.017 27663910

[B7] Aly M. M. Abu Alsoud N. M. Elrobh M. S. Al Johani S. M. Balkhy H. H. (2016). High prevalence of the PER-1 gene among carbapenem-resistant *Acinetobacter baumannii* in Riyadh, Saudi Arabia. Eur. J. Clin. Microbiol. Infect Dis. 35, 1759–1766. 10.1007/s10096-016-2723-8 27527351

[B8] Anthony K. B. Fishman N. O. Linkin D. R. Gasink L. B. Edelstein P. H. Lautenbach E. (2008). Clinical and microbiological outcomes of serious infections with multidrug-resistant gram-negative organisms treated with tigecycline. Clin. Infect. Dis. 46, 567–570. 10.1086/526775 18199038

[B9] Antunes L. C. Imperi F. Carattoli A. Visca P. (2011). Deciphering the multifactorial nature of *Acinetobacter baumannii* pathogenicity. PLoS ONE 6:e22674. 10.1371/journal.pone.0022674 21829642 PMC3148234

[B10] Antunes L. C. Imperi F. Minandri F. Visca P. (2012). *In vitro* and *in vivo* antimicrobial activities of gallium nitrate against multidrug-resistant *Acinetobacter baumannii*. Antimicrob. Agents Chemother. 56, 5961–5970. 10.1128/AAC.01519-12 22964249 PMC3486561

[B11] Aranda J. Bardina C. Beceiro A. Rumbo S. Cabral M. P. Barbe J.. (2011). *Acinetobacter baumannii* RecA protein in repair of DNA damage, antimicrobial resistance, general stress response, and virulence. J. Bacteriol. 193, 3740–3747. 10.1128/JB.00389-11 21642465 PMC3147500

[B12] Aranda J. Lopez M. Leiva E. Magan A. Adler B. Bou G.. (2014). Role of *Acinetobacter baumannii* UmuD homologs in antibiotic resistance acquired through DNA damage-induced mutagenesis. Antimicrob. Agents Chemother. 58, 1771–1773. 10.1128/AAC.02346-13 24342640 PMC3957856

[B13] Arivett B. A. Fiester S. E. Ohneck E. J. Penwell W. F. Kaufman C. M. Relich R. F.. (2015). Antimicrobial activity of Gallium Protoporphyrin IX against *Acinetobacter baumannii* strains displaying different antibiotic resistance phenotypes. Antimicrob. Agents Chemother. 59, 7657–7665. 10.1128/AAC.01472-15 26416873 PMC4649216

[B14] Arroyo L. A. Herrera C. M. Fernandez L. Hankins J. V. Trent M. S. Hancock R. E. (2011). The *pmrCAB* operon mediates polymyxin resistance in *Acinetobacter baumannii* ATCC 17978 and clinical isolates through phosphoethanolamine modification of lipid A. Antimicrob. Agents Chemother. 55, 3743–3751. 10.1128/AAC.00256-11 21646482 PMC3147623

[B15] Asahara T. Takahashi A. Yuki N. Kaji R. Takahashi T. Nomoto K. (2016). Protective effect of a synbiotic against multidrug-resistant *Acinetobacter baumannii* in a Murine Infection Model. Antimicrob. Agents Chemother. 60, 3041–3050. 10.1128/AAC.02928-15 26953197 PMC4862511

[B16] Aydemir H. Akduman D. Piskin N. Comert F. Horuz E. Terzi A.. (2013). Colistin vs. the combination of colistin and rifampicin for the treatment of carbapenem-resistant *Acinetobacter baumannii* ventilator-associated pneumonia. Epidemiol. Infect. 141, 1214–1222. 10.1017/S095026881200194X 22954403 PMC9151808

[B17] Bae S. Kim M. C. Park S. J. Kim H. S. Sung H. Kim M. N.. (2016). *In vitro* synergistic activity of antimicrobial agents in combination against clinical isolates of colistin-resistant *Acinetobacter baumannii*. Antimicrob. Agents Chemother. 60, 6774–6779. 10.1128/AAC.00839-16 27600048 PMC5075085

[B18] Bakour S. Alsharapy S. A. Touati A. Rolain J. M. (2014). Characterization of *Acinetobacter baumannii* clinical isolates carrying *bla*_OXA-23_carbapenemase and 16S rRNA methylase armA genes in Yemen. Microb. Drug Resist. 20, 604–609. 10.1089/mdr.2014.0018 24901296

[B19] Barksdale S. M. Hrifko E. J. Chung E. M. van Hoek M. L. (2016). Peptides from American alligator plasma are antimicrobial against multi-drug resistant bacterial pathogens including *Acinetobacter baumannii*. BMC Microbiol. 16:189. 10.1186/s12866-016-0799-z 27542832 PMC4992317

[B20] Basler M. Ho B. T. Mekalanos J. J. (2013). Tit-for-tat: type VI secretion system counterattack during bacterial cell-cell interactions. Cell 152, 884–894. 10.1016/j.cell.2013.01.042 23415234 PMC3616380

[B21] Bassetti M. Repetto E. Righi E. Boni S. Diverio M. Molinari M. P.. (2008). Colistin and rifampicin in the treatment of multidrug-resistant *Acinetobacter baumannii* infections. J. Antimicrob. Chemother. 61, 417–420. 10.1093/jac/dkm509 18174197

[B22] Batirel A. Balkan I. I. Karabay O. Agalar C. Akalin S. Alici O.. (2014). Comparison of colistin-carbapenem, colistin-sulbactam, and colistin plus other antibacterial agents for the treatment of extremely drug-resistant *Acinetobacter baumannii* bloodstream infections. Eur. J. Clin. Microbiol. Infect. Dis. 33, 1311–1322. 10.1007/s10096-014-2070-6 24532009

[B23] Beceiro A. Llobet E. Aranda J. Bengoechea J. A. Doumith M. Hornsey M.. (2011). Phosphoethanolamine modification of lipid A in colistin-resistant variants of Acinetobacter baumannii mediated by the *pmrAB* two-component regulatory system. Antimicrob. Agents Chemother. 55, 3370–3379. 10.1128/AAC.00079-11 21576434 PMC3122444

[B24] Beceiro A. Tomas M. Bou G. (2013). Antimicrobial resistance and virulence: a successful or deleterious association in the bacterial world? Clin. Microbiol. Rev. 26, 185–230. 10.1128/CMR.00059-12 23554414 PMC3623377

[B25] Bentancor L. V. Camacho-Peiro A. Bozkurt-Guzel C. Pier G. B. Maira-Litran T. (2012a). Identification of Ata, a multifunctional trimeric autotransporter of *Acinetobacter baumannii*. J. Bacteriol. 194, 3950–3960. 10.1128/JB.06769-11 22609912 PMC3416510

[B26] Bentancor L. V. Routray A. Bozkurt-Guzel C. Camacho-Peiro A. Pier G. B. Maira-Litran T. (2012b). Evaluation of the trimeric autotransporter Ata as a vaccine candidate against *Acinetobacter baumannii* infections. Infect. Immun. 80, 3381–3388. 10.1128/IAI.06096-11 22825448 PMC3457567

[B27] Bergogne-Berezin E. Towner K. J. (1996). *Acinetobacter* spp. as nosocomial pathogens: microbiological, clinical, and epidemiological features. Clin. Microbiol Rev. 9, 148–165. 8964033 10.1128/cmr.9.2.148 PMC172888

[B28] Bernstein L. R. (1998). Mechanisms of therapeutic activity for gallium. Pharmacol. Rev. 50, 665–682. 9860806

[B29] Bhuiyan M. S. Ellett F. Murray G. L. Kostoulias X. Cerqueira G. M. Schulze K. E.. (2016). *Acinetobacter baumannii* phenylacetic acid metabolism influences infection outcome through a direct effect on neutrophil chemotaxis. Proc. Natl. Acad. Sci. U.S.A. 113, 9599–9604. 10.1073/pnas.1523116113 27506797 PMC5003227

[B30] Biancofiore G. Tascini C. Bisa M. Gemignani G. Bindi M. L. Leonildi A.. (2007). Colistin, meropenem and rifampin in a combination therapy for multi-drug-resistant *Acinetobacter baumannii* multifocal infection. A case report. Minerva. Anestesiol. 73, 181–185. 17159765

[B31] Biglari S. Hanafiah A. Mohd Puzi S. Ramli R. Rahman M. Lopes B. S. (2016). Antimicrobial resistance mechanisms and genetic diversity of multidrug-resistant *Acinetobacter baumannii* isolated from a teaching hospital in Malaysia. Microb. Drug Resist. [Epub ahead of print]. 10.1089/mdr.2016.0130 27854165

[B32] Blanchard C. Barnett P. Perlmutter J. Dunman P. M. (2014). Identification of *Acinetobacter baumannii* serum-associated antibiotic efflux pump inhibitors. Antimicrob. Agents Chemother. 58, 6360–6370. 10.1128/AAC.03535-14 25114126 PMC4249429

[B33] Bogaerts P. Naas T. El Garch F. Cuzon G. Deplano A. Delaire T.. (2010). GES extended-spectrum β-lactamases in *Acinetobacter baumannii* isolates in Belgium. Antimicrob. Agents Chemother. 54, 4872–4878. 10.1128/AAC.00871-10 20805394 PMC2976171

[B34] Boll J. M. Tucker A. T. Klein D. R. Beltran A. M. Brodbelt J. S. Davies B. W.. (2015). Reinforcing lipid A acylation on the cell surface of *Acinetobacter baumannii* promotes cationic antimicrobial peptide resistance and desiccation survival. MBio 6, e00478–e00415. 10.1128/mBio.00478-15 25991684 PMC4442142

[B35] Bonnin R. A. Poirel L. Licker M. Nordmann P. (2011a). Genetic diversity of carbapenem-hydrolysing β-lactamases in *Acinetobacter baumannii* from Romanian hospitals. Clin. Microbiol. Infect. 17, 1524–1528. 10.1111/j.1469-0691.2011.03622.x 21883667

[B36] Bonnin R. A. Poirel L. Naas T. Pirs M. Seme K. Schrenzel J.. (2012). Dissemination of New Delhi metallo-β-lactamases-1-producing *Acinetobacter baumannii* in Europe. Clin. Microbiol. Infect. 18, E362–E365. 10.1111/j.1469-0691.2012.03928.x 22738206

[B37] Bonnin R. A. Potron A. Poirel L. Lecuyer H. Neri R. Nordmann P. (2011b). PER-7, an extended-spectrum β-lactamases with increased activity toward broad-spectrum cephalosporins in *Acinetobacter baumannii*. Antimicrob. Agents Chemother. 55, 2424–2427. 10.1128/AAC.01795-10 21383087 PMC3088246

[B38] Borgeaud S. Metzger L. C. Scrignari T. Blokesch M. (2015). The type VI secretion system of *Vibrio cholerae* fosters horizontal gene transfer. Science. 347, 63–67. 10.1126/science.1260064 25554784

[B39] Bou G. Cervero G. Dominguez M. A. Quereda C. Martinez-Beltran J. (2000a). Characterization of a nosocomial outbreak caused by a multiresistant *Acinetobacter baumannii* strain with a carbapenem-hydrolyzing enzyme: high-level carbapenem resistance in *A. baumannii* is not due solely to the presence of β-lactamases. J. Clin. Microbiol. 38, 3299–3305. 10970374 10.1128/jcm.38.9.3299-3305.2000 PMC87377

[B40] Bou G. Martinez-Beltran J. (2000). Cloning, nucleotide sequencing, and analysis of the gene encoding an AmpC β-lactamases in *Acinetobacter baumannii*. Antimicrob. Agents Chemother. 44, 428–432. 10.1128/AAC.44.2.428-432.2000 10639377 PMC89698

[B41] Bou G. Oliver A. Martinez-Beltran J. (2000b). OXA-24, a novel class D β-lactamases with carbapenemase activity in an *Acinetobacter baumannii* clinical strain. Antimicrob. Agents Chemother. 44, 1556–1561. 10.1128/AAC.44.6.1556-1561.2000 10817708 PMC89912

[B42] Boucher H. W. Talbot G. H. Bradley J. S. Edwards J. E. Gilbert D. Rice L. B.. (2009). Bad bugs, no drugs: no ESKAPE! An update from the Infectious Diseases Society of America. Clin. Infect. Dis. 48, 1–12. 10.1086/595011 19035777

[B43] Bowler S. L. Spychala C. N. McElheny C. L. Mettus R. T. Doi Y. (2016). *In vitro* activity of fusidic acid-containing combinations against carbapenem-resistant *Acinetobacter baumannii* clinical strains. Antimicrob. Agents Chemother. 60, 5101. 10.1128/AAC.01124-16 27270280 PMC4958153

[B44] Briers Y. Walmagh M. Van Puyenbroeck V. Cornelissen A. Cenens W. Aertsen A.. (2014). Engineered endolysin-based “Artilysins” to combat multidrug-resistant gram-negative pathogens. MBio 5, e01379–14. 10.1128/mBio.01379-14 24987094 PMC4161244

[B45] Brigante G. Migliavacca R. Bramati S. Motta E. Nucleo E. Manenti M.. (2012). Emergence and spread of a multidrug-resistant *Acinetobacter baumannii* clone producing both the carbapenemase OXA-23 and the 16S rRNA methylase ArmA. J. Med. Microbiol. 61, 653–661. 10.1099/jmm.0.040980-0 22282459

[B46] Brown S. Young H. K. Amyes S. G. (2005). Characterisation of OXA-51, a novel class D carbapenemase found in genetically unrelated clinical strains of *Acinetobacter baumannii* from Argentina. Clin. Microbiol. Infect. 11, 15–23. 10.1111/j.1469-0691.2004.01016.x 15649299

[B47] Cai Y. Chai D. Wang R. Liang B. Bai N. (2012). Colistin resistance of *Acinetobacter baumannii*: clinical reports, mechanisms and antimicrobial strategies. J. Antimicrob. Chemother. 67, 1607–1615. 10.1093/jac/dks084 22441575

[B48] Camarena L. Bruno V. Euskirchen G. Poggio S. Snyder M. (2010). Molecular mechanisms of ethanol-induced pathogenesis revealed by RNA-sequencing. PLoS Pathog. 6:e1000834. 10.1371/journal.ppat.1000834 20368969 PMC2848557

[B49] Candel F. J. Calvo N. Head J. Sanchez A. Matesanz M. Culebras E.. (2010). A combination of tigecycline, colistin, and meropenem against multidrug-resistant *Acinetobacter baumannii* bacteremia in a renal transplant recipient: pharmacodynamic and microbiological aspects. Rev. Esp. Quimioter. 23, 103–108. 20559610

[B50] Cannon J. P. Lee T. A. Clark N. M. Setlak P. Grim S. A. (2014). The risk of seizures among the carbapenems: a meta-analysis. J. Antimicrob. Chemother. 69, 2043–2055. 10.1093/jac/dku111 24744302

[B51] Carruthers M. D. Nicholson P. A. Tracy E. N. Munson R. S. Jr. (2013). *Acinetobacter baumannii* utilizes a type VI secretion system for bacterial competition. PLoS ONE 8:e59388. 10.1371/journal.pone.0059388 23527179 PMC3602014

[B52] Castanheira M. Mendes R. E. Jones R. N. (2014). Update on *Acinetobacter* species: mechanisms of antimicrobial resistance and contemporary *in vitro* activity of minocycline and other treatment options. Clin. Infect. Dis. 59 (Suppl. 6), S367–S373. 10.1093/cid/ciu706 25371512

[B53] Catel-Ferreira M. Coadou G. Molle V. Mugnier P. Nordmann P. Siroy A.. (2011). Structure-function relationships of CarO, the carbapenem resistance-associated outer membrane protein of *Acinetobacter baumannii*. J. Antimicrob. Chemother. 66, 2053–2056. 10.1093/jac/dkr267 21705362

[B54] Centers for Disease Prevention (2004). *Acinetobacter baumannii* infections among patients at military medical facilities treating injured U.S. service members, 2002–2004. MMWR Morb. Mortal. Wkly. Rep. 53, 1063–1066. 15549020

[B55] Cerqueira G. M. Kostoulias X. Khoo C. Aibinu I. Qu Y. Traven A.. (2014). A global virulence regulator in *Acinetobacter baumannii* and its control of the phenylacetic acid catabolic pathway. J. Infect. Dis. 210, 46–55. 10.1093/infdis/jiu024 24431277

[B56] Chagas T. P. Carvalho K. R. de Oliveira Santos I. C. Carvalho-Assef A. P. Asensi M. D. (2014). Characterization of carbapenem-resistant *Acinetobacter baumannii* in Brazil (2008-2011): countrywide spread of OXA-23-producing clones (CC15 and CC79). Diagn. Microbiol. Infect. Dis. 79, 468–472. 10.1016/j.diagmicrobio.2014.03.006 24880823

[B57] Chang H. C. Wei Y. F. Dijkshoorn L. Vaneechoutte M. Tang C. T. Chang T. C. (2005). Species-level identification of isolates of the *Acinetobacter calcoaceticus*-*Acinetobacter baumannii* complex by sequence analysis of the 16S-23S rRNA gene spacer region. J. Clin. Microbiol. 43, 1632–1639. 10.1128/JCM.43.4.1632-1639.2005 15814977 PMC1081347

[B58] Chang K. C. Lin M. F. Lin N. T. Wu W. J. Kuo H. Y. Lin T. Y.. (2012). Clonal spread of multidrug-resistant *Acinetobacter baumannii* in eastern Taiwan. J. Microbiol. Immunol. Infect. 45, 37–42. 10.1016/j.jmii.2011.09.019 22154678

[B59] Cheah S. E. Li J. Tsuji B. T. Forrest A. Bulitta J. B. Nation R. L. (2016). Colistin and polymyxin B dosage regimens against *Acinetobacter baumannii*: differences in activity and the emergence of resistance. Antimicrob. Agents Chemother. 60, 3921–3933. 10.1128/AAC.02927-15 27067324 PMC4914682

[B60] Chen C. H. Young T. G. Huang C. C. (2006). Predictive biomarkers for drug-resistant *Acinetobacter baumannii* isolates with *bla*_TEM-1_, AmpC-type *bla* and integrase 1 genotypes. J. Microbiol. Immunol. Infect. 39, 372–379. 17066198

[B61] Chen Q. Li X. Zhou H. Jiang Y. Chen Y. Hua X.. (2014). Decreased susceptibility to tigecycline in *Acinetobacter baumannii* mediated by a mutation in *trm* encoding SAM-dependent methyltransferase. J. Antimicrob. Chemother. 69, 72–76. 10.1093/jac/dkt319 23928024

[B62] Chen T. L. Lee Y. T. Kuo S. C. Hsueh P. R. Chang F. Y. Siu L. K.. (2010). Emergence and distribution of plasmids bearing the *bla*_OXA-51_-like gene with an upstream IS*Aba1* in carbapenem-resistant *Acinetobacter baumannii* isolates in Taiwan. Antimicrob. Agents Chemother. 54, 4575–4581. 10.1128/AAC.00764-10 20713680 PMC2976157

[B63] Chen T. L. Wu R. C. Shaio M. F. Fung C. P. Cho W. L. (2008). Acquisition of a plasmid-borne *bla*_OXA-58_ gene with an upstream IS*1008* insertion conferring a high level of carbapenem resistance to *Acinetobacter baumannii*. Antimicrob. Agents Chemother. 52, 2573–2580. 10.1128/AAC.00393-08 18443121 PMC2443897

[B64] Chen Y. Zhou Z. Jiang Y. Yu Y. (2011). Emergence of NDM-1-producing *Acinetobacter baumannii* in China. J. Antimicrob. Chemother. 66, 1255–1259. 10.1093/jac/dkr082 21398294

[B65] Chihi H. Bonnin R. A. Bourouis A. Mahrouki S. Besbes S. Moussa M. B.. (2016). GES-11-producing *Acinetobacter baumannii* clinical isolates from Tunisian hospitals: long-term dissemination of GES-type carbapenemases in North Africa. J. Glob. Antimicrob. Resist. 5, 47–50. 10.1016/j.jgar.2016.03.005 27436466

[B66] Chin C. Y. Gregg K. A. Napier B. A. Ernst R. K. Weiss D. S. (2015). A PmrB-regulated deacetylase required for lipid A modification and polymyxin resistance in *Acinetobacter baumannii*. Antimicrob. Agents Chemother. 59, 7911–7914. 10.1128/AAC.00515-15 26459891 PMC4649237

[B67] Chiu C. H. Liu Y. H. Wang Y. C. Lee Y. T. Kuo S. C. Chen T. L.. (2016). *In vitro* activity of SecA inhibitors in combination with carbapenems against carbapenem-hydrolysing class D β-lactamase-producing *Acinetobacter baumannii*. J. Antimicrob. Chemother. 71, 3441–3448. 10.1093/jac/dkw331 27543656

[B68] Cho Y. J. Moon D. C. Jin J. S. Choi C. H. Lee Y. C. Lee J. C. (2009). Genetic basis of resistance to aminoglycosides in *Acinetobacter* spp. and spread of *armA* in *Acinetobacter baumannii* sequence group 1 in Korean hospitals. Diagn. Microbiol. Infect. Dis. 64, 185–190. 10.1016/j.diagmicrobio.2009.02.010 19361944

[B69] Choi C. H. Hyun S. H. Lee J. Y. Lee J. S. Lee Y. S. Kim S. A.. (2008a). *Acinetobacter baumannii* outer membrane protein A targets the nucleus and induces cytotoxicity. Cell. Microbiol. 10, 309–319. 10.1111/j.1462-5822.2007.01041.x 17760880

[B70] Choi C. H. Lee E. Y. Lee Y. C. Park T. I. Kim H. J. Hyun S. H.. (2005). Outer membrane protein 38 of *Acinetobacter baumannii* localizes to the mitochondria and induces apoptosis of epithelial cells. Cell Microbiol. 7, 1127–1138. 10.1111/j.1462-5822.2005.00538.x 16008580

[B71] Choi C. H. Lee J. S. Lee Y. C. Park T. I. Lee J. C. (2008b). *Acinetobacter baumannii* invades epithelial cells and outer membrane protein A mediates interactions with epithelial cells. BMC Microbiol. 8:216. 10.1186/1471-2180-8-216 19068136 PMC2615016

[B72] Chu Y. W. Afzal-Shah M. Houang E. T. Palepou M. I. Lyon D. J. Woodford N.. (2001). IMP-4, a novel metallo-β-lactamase from nosocomial *Acinetobacter* spp. collected in Hong Kong between 1994 and 1998. Antimicrob. Agents Chemother. 45, 710–714. 10.1128/AAC.45.3.710-714.2001 11181348 PMC90361

[B73] Chung J. H. Bhat A. Kim C. J. Yong D. Ryu C. M. (2016). Combination therapy with polymyxin B and netropsin against clinical isolates of multidrug-resistant *Acinetobacter baumannii*. Sci. Rep. 6:28168. 10.1038/srep28168 27306928 PMC4910107

[B74] Cirioni O. Simonetti O. Pierpaoli E. Barucca A. Ghiselli R. Orlando F.. (2016). Colistin enhances therapeutic efficacy of daptomycin or teicoplanin in a murine model of multiresistant *Acinetobacter baumannii* sepsis. Diagn. Microbiol. Infect. Dis. 86, 392–398. 10.1016/j.diagmicrobio.2016.09.010 27712928

[B75] Cirz R. T. Romesberg F. E. (2007). Controlling mutation: intervening in evolution as a therapeutic strategy. Crit. Rev. Biochem. Mol. Biol. 42, 341–354. 10.1080/10409230701597741 17917871

[B76] Cisneros J. M. Rodríguez-Baño J. (2002). Nosocomial bacteremia due to *Acinetobacter baumannii*: epidemiology, clinical features and treatment. Clin. Microbiol. Infect. 8, 687–693. 10.1046/j.1469-0691.2002.00487.x 12445005

[B77] Clemmer K. M. Bonomo R. A. Rather P. N. (2011). Genetic analysis of surface motility in *Acinetobacter baumannii*. Microbiology 157, 2534–2544. 10.1099/mic.0.049791-0 21700662 PMC3352170

[B78] Corbin B. D. Seeley E. H. Raab A. Feldmann J. Miller M. R. Torres V. J.. (2008). Metal chelation and inhibition of bacterial growth in tissue abscesses. Science 319, 962–965. 10.1126/science.1152449 18276893

[B79] Cordoba J. Coronado-Alvarez N. M. Parra D. Parra-Ruiz J. (2015). *In vitro* activities of novel antimicrobial combinations against extensively drug-resistant *Acinetobacter baumannii*. Antimicrob. Agents Chemother. 59, 7316–7319. 10.1128/AAC.00493-15 26369956 PMC4649143

[B80] Corvec S. Caroff N. Espaze E. Giraudeau C. Drugeon H. Reynaud A. (2003). AmpC cephalosporinase hyperproduction in *Acinetobacter baumannii* clinical strains. J. Antimicrob. Chemother. 52, 629–635. 10.1093/jac/dkg407 12951337

[B81] Corvec S. Poirel L. Naas T. Drugeon H. Nordmann P. (2007). Genetics and expression of the carbapenem-hydrolyzing oxacillinase gene *bla*_OXA−23_ in *Acinetobacter baumannii*. Antimicrob. Agents Chemother. 51, 1530–1533. 10.1128/AAC.01132-06 17220422 PMC1855470

[B82] Coyne S. Courvalin P. Perichon B. (2011). Efflux-mediated antibiotic resistance in *Acinetobacter* spp. Antimicrob. Agents Chemother. 55, 947–953. 10.1128/AAC.01388-10 21173183 PMC3067115

[B83] Coyne S. Rosenfeld N. Lambert T. Courvalin P. Perichon B. (2010). Overexpression of resistance-nodulation-cell division pump AdeFGH confers multidrug resistance in *Acinetobacter baumannii*. Antimicrob. Agents Chemother. 54, 4389–4393. 10.1128/AAC.00155-10 20696879 PMC2944555

[B84] Croxatto A. Prod'hom G. Greub G. (2012). Applications of MALDI-TOF mass spectrometry in clinical diagnostic microbiology. FEMS Microbiol. Rev. 36, 380–407. 10.1111/j.1574-6976.2011.00298.x 22092265

[B85] Curcio D. Fernandez F. (2008). Tigecycline for *Acinetobacter baumannii* infection: other considerations. Clin. Infect. Dis. 46, 1797–1798; author reply 1798–1799. 10.1086/588051 18462123

[B86] Damier-Piolle L. Magnet S. Bremont S. Lambert T. Courvalin P. (2008). AdeIJK, a resistance-nodulation-cell division pump effluxing multiple antibiotics in *Acinetobacter baumannii*. Antimicrob. Agents Chemother. 52, 557–562. 10.1128/AAC.00732-07 18086852 PMC2224764

[B87] Davison M. Treangen T. J. Koren S. Pop M. Bhaya D. (2016). Diversity in a polymicrobial community revealed by analysis of viromes, endolysins and CRISPR spacers. PLoS ONE 11:e0160574. 10.1371/journal.pone.0160574 27611571 PMC5017753

[B88] de Breij A. Dijkshoorn L. Lagendijk E. van der Meer J. Koster A. Bloemberg G.. (2010). Do biofilm formation and interactions with human cells explain the clinical success of *Acinetobacter baumannii*? PLoS ONE 5:e10732. 10.1371/journal.pone.0010732 20505779 PMC2874002

[B89] Defraine V. Schuermans J. Grymonprez B. Govers S. K. Aertsen A. Fauvart M.. (2016). Efficacy of artilysin Art-175 against resistant and persistent *Acinetobacter baumannii*. Antimicrob. Agents Chemother. 60, 3480–3488. 10.1128/AAC.00285-16 27021321 PMC4879360

[B90] del Mar Tomas M. Beceiro A. Perez A. Velasco D. Moure R. Villanueva R.. (2005). Cloning and functional analysis of the gene encoding the 33- to 36-kilodalton outer membrane protein associated with carbapenem resistance in *Acinetobacter baumannii*. Antimicrob. Agents Chemother. 49, 5172–5175. 10.1128/AAC.49.12.5172-5175.2005 16304197 PMC1315955

[B91] Deng M. Zhu M. H. Li J. J. Bi S. Sheng Z. K. Hu F. S.. (2014). Molecular epidemiology and mechanisms of tigecycline resistance in clinical isolates of *Acinetobacter baumannii* from a Chinese university hospital. Antimicrob. Agents Chemother. 58, 297–303. 10.1128/AAC.01727-13 24165187 PMC3910737

[B92] de Sa Cavalcanti F. L. Mendes-Marques C. L. Vasconcelos C. R. de Lima Campos T. Rezende A. M. Xavier D. E.. (2016). High frequency of OXA-253-producing *Acinetobacter baumannii* in different hospitals in Recife, Brazil: a new threat? Antimicrob. Agents Chemother. 61:e01309–16. 10.1128/AAC.01309-16 27855080 PMC5192159

[B93] Dhabaan G. N. AbuBakar S. Cerqueira G. M. Al-Haroni M. Pang S. P. Hassan H. (2015). Imipenem treatment induces expression of important genes and phenotypes in a resistant *Acinetobacter baumannii* isolate. Antimicrob. Agents Chemother. 60, 1370–1376. 10.1128/AAC.01696-15 26666943 PMC4775920

[B94] Dijkshoorn L. Aucken H. Gerner-Smidt P. Janssen P. Kaufmann M. E. Garaizar J.. (1996). Comparison of outbreak and nonoutbreak *Acinetobacter baumannii* strains by genotypic and phenotypic methods. J. Clin. Microbiol. 34, 1519–1525. 8735109 10.1128/jcm.34.6.1519-1525.1996 PMC229053

[B95] Dizbay M. Tozlu D. K. Cirak M. Y. Isik Y. Ozdemir K. Arman D. (2010). *In vitro* synergistic activity of tigecycline and colistin against XDR-*Acinetobacter baumannii*. J. Antibiot. (Tokyo) 63, 51–53. 10.1038/ja.2009.117 19942947

[B96] Doi Y. Murray G. L. Peleg A. Y. (2015). *Acinetobacter baumannii*: evolution of antimicrobial resistance-treatment options. Semin. Respir. Crit. Care Med. 36, 85–98. 10.1055/s-0034-1398388 25643273 PMC4465586

[B97] Doi Y. Wachino J. Yamane K. Shibata N. Yagi T. Shibayama K.. (2004). Spread of novel aminoglycoside resistance gene *aac(6*′*)*-Iad among *Acinetobacter* clinical isolates in Japan. Antimicrob. Agents Chemother. 48, 2075–2080. 10.1128/AAC.48.6.2075-2080.2004 15155202 PMC415623

[B98] Donnarumma F. Sergi S. Indorato C. Mastromei G. Monnanni R. Nicoletti P.. (2010). Molecular characterization of acinetobacter isolates collected in intensive care units of six hospitals in Florence, Italy, during a 3-year surveillance program: a population structure analysis. J. Clin. Microbiol. 48, 1297–1304. 10.1128/JCM.01916-09 20181903 PMC2849567

[B99] Dortet L. Bonnin R. A. Bernabeu S. Escaut L. Vittecoq D. Girlich D.. (2016). First occurrence of OXA-72-producing *Acinetobacter baumannii* in Serbia. Antimicrob. Agents Chemother. 60, 5724–5730. 10.1128/AAC.01016-16 27431216 PMC5038319

[B100] Dubois D. Prasadarao N. V. Mittal R. Bret L. Roujou-Gris M. Bonnet R. (2009). CTX-M β-lactamase production and virulence of *Escherichia coli* K1. Emerg. Infect. Dis. 15, 1988–1990. 10.3201/eid1512.090928 19961682 PMC3044546

[B101] Dubrovskaya Y. Prasad N. Lee Y. Esaian D. Figueroa D. A. Tam V. H. (2015). Risk factors for nephrotoxicity onset associated with polymyxin B therapy. J. Antimicrob. Chemother. 70, 1903–1907. 10.1093/jac/dkv014 25652747

[B102] Dupont M. Pages J. M. Lafitte D. Siroy A. Bollet C. (2005). Identification of an OprD homologue in *Acinetobacter baumannii*. J. Proteome. Res. 4, 2386–2390. 10.1021/pr050143q 16335991

[B103] Elhosseiny N. M. Amin M. A. Yassin A. S. Attia A. S. (2015). *Acinetobacter baumannii* universal stress protein A plays a pivotal role in stress response and is essential for pneumonia and sepsis pathogenesis. Int. J. Med. Microbiol. 305, 114–123. 10.1016/j.ijmm.2014.11.008 25466824

[B104] Elhosseiny N. M. El-Tayeb O. M. Yassin A. S. Lory S. Attia A. S. (2016). The secretome of *Acinetobacter baumannii* ATCC 17978 type II secretion system reveals a novel plasmid encoded phospholipase that could be implicated in lung colonization. Int. J. Med. Microbiol. 306, 633–641. 10.1016/j.ijmm.2016.09.006 27713027

[B105] Ellis T. N. Kuehn M. J. (2010). Virulence and immunomodulatory roles of bacterial outer membrane vesicles. Microbiol. Mol. Biol. Rev. 74, 81–94. 10.1128/MMBR.00031-09 20197500 PMC2832350

[B106] Endimiani A. Luzzaro F. Migliavacca R. Mantengoli E. Hujer A. M. Hujer K. M.. (2007). Spread in an Italian hospital of a clonal *Acinetobacter baumannii s*train producing the TEM-92 extended-spectrum β-lactamase. Antimicrob. Agents Chemother. 51, 2211–2214. 10.1128/AAC.01139-06 17404005 PMC1891385

[B107] Erridge C. Moncayo-Nieto O. L. Morgan R. Young M. Poxton I. R. (2007). *Acinetobacter baumannii* lipopolysaccharides are potent stimulators of human monocyte activation via Toll-like receptor 4 signalling. J. Med. Microbiol. 56, 165–171. 10.1099/jmm.0.46823-0 17244795

[B108] Espinal P. Fugazza G. Lopez Y. Kasma M. Lerman Y. Malhotra-Kumar S.. (2011). Dissemination of an NDM-2-producing *Acinetobacter baumannii* clone in an Israeli rehabilitation center. Antimicrob. Agents Chemother. 55, 5396–5398. 10.1128/AAC.00679-11 21825296 PMC3195055

[B109] Evans B. A. Brown S. Hamouda A. Findlay J. Amyes S. G. (2007). Eleven novel OXA-51-like enzymes from clinical isolates of *Acinetobacter baumannii*. Clin. Microbiol. Infect. 13, 1137–1138. 10.1111/j.1469-0691.2007.01828.x 17850339

[B110] Fajardo Bonin R. Chapeaurouge A. Perales J. da Silva J. G. Jr. do Nascimento H. J. D'Alincourt Carvalho Assef A. P.. (2014). Identification of immunogenic proteins of the bacterium *Acinetobacter baumannii* using a proteomic approach. Proteomics Clin. Appl. 8, 916–923. 10.1002/prca.201300133 24899143

[B111] Fan B. Guan J. Wang X. Cong Y. (2016). Activity of colistin in combination with meropenem, tigecycline, fosfomycin, fusidic acid, rifampin or sulbactam against extensively drug-resistant *Acinetobacter baumannii* in a murine thigh-infection model. PLoS ONE 11:e0157757. 10.1371/journal.pone.0157757 27315107 PMC4912081

[B112] Fang F. Wang S. Dang Y. X. Wang X. Yu G. Q. (2016). Molecular characterization of carbapenemase genes in *Acinetobacter baumannii* in China. Genet. Mol. Res. 15:gmr7432. 10.4238/gmr.15017432 27051026

[B113] Fernandez-Cuenca F. Smani Y. Gomez-Sanchez M. C. Docobo-Perez F. Caballero-Moyano F. J. Dominguez-Herrera J.. (2011). Attenuated virulence of a slow-growing pandrug-resistant *Acinetobacter baumannii* is associated with decreased expression of genes encoding the porins CarO and OprD-like. Int. J. Antimicrob. Agents 38, 548–549. 10.1016/j.ijantimicag.2011.08.002 21940150

[B114] Fernando D. Kumar A. (2012). Growth phase-dependent expression of RND efflux pump- and outer membrane porin-encoding genes in *Acinetobacter baumannii* ATCC 19606. J. Antimicrob. Chemother. 67, 569–572. 10.1093/jac/dkr519 22146875

[B115] Fiester S. E. Arivett B. A. Schmidt R. E. Beckett A. C. Ticak T. Carrier M. V.. (2016). Iron-regulated phospholipase C activity contributes to the cytolytic activity and virulence of *Acinetobacter baumannii*. PLoS ONE 11:e0167068. 10.1371/journal.pone.0167068 27875572 PMC5119829

[B116] Flores-Diaz M. Monturiol-Gross L. Naylor C. Alape-Giron A. Flieger A. (2016). Bacterial sphingomyelinases and phospholipases as virulence factors. Microbiol. Mol. Biol. Rev. 80, 597–628. 10.1128/MMBR.00082-15 27307578 PMC4981679

[B117] Fonseca E. L. Scheidegger E. Freitas F. S. Cipriano R. Vicente A. C. (2013). Carbapenem-resistant *Acinetobacter baumannii* from Brazil: role of *carO* alleles expression and *bla*_OXA-23_ gene. BMC Microbiol. 13:245. 10.1186/1471-2180-13-245 24195496 PMC4228306

[B118] Fournier P. E. Richet H. (2006). The epidemiology and control of *Acinetobacter baumannii* in health care facilities. Clin. Infect. Dis. 42, 692–699. 10.1086/500202 16447117

[B119] Fournier P. E. Vallenet D. Barbe V. Audic S. Ogata H. Poirel L.. (2006). Comparative genomics of multidrug resistance in *Acinetobacter baumannii*. PLoS Genet. 2:e7. 10.1371/journal.pgen.0020007 16415984 PMC1326220

[B120] Fulnecky E. J. Wright D. Scheld W. M. Kanawati L. Shoham S. (2005). Amikacin and colistin for treatment of *Acinetobacter baumannii* meningitis. J. Infect. 51, e249–e251. 10.1016/j.jinf.2005.04.003 15913780

[B121] Gaddy J. A. Arivett B. A. McConnell M. J. Lopez-Rojas R. Pachon J. Actis L. A. (2012). Role of acinetobactin-mediated iron acquisition functions in the interaction of *Acinetobacter baumannii* strain ATCC 19606T with human lung epithelial cells, *Galleria mellonella* caterpillars, and mice. Infect. Immun. 80, 1015–1024. 10.1128/IAI.06279-11 22232188 PMC3294665

[B122] Gaddy J. A. Tomaras A. P. Actis L. A. (2009). The *Acinetobacter baumannii* 19606 OmpA protein plays a role in biofilm formation on abiotic surfaces and in the interaction of this pathogen with eukaryotic cells. Infect. Immun. 77, 3150–3160. 10.1128/IAI.00096-09 19470746 PMC2715673

[B123] Gales A. C. Tognim M. C. Reis A. O. Jones R. N. Sader H. S. (2003). Emergence of an IMP-like metallo-enzyme in an *Acinetobacter baumannii* clinical strain from a Brazilian teaching hospital. Diagn. Microbiol. Infect. Dis. 45, 77–79. 10.1016/S0732-8893(02)00500-X 12573555

[B124] Gallego L. Towner K. J. (2001). Carriage of class 1 integrons and antibiotic resistance in clinical isolates of *Acinetobacter baumannii* from northern Spain. J. Med. Microbiol. 50, 71–77. 10.1099/0022-1317-50-1-71 11192508

[B125] Garcia-Quintanilla M. Carretero-Ledesma M. Moreno-Martinez P. Martin-Pena R. Pachon J. McConnell M. J. (2015). Lipopolysaccharide loss produces partial colistin dependence and collateral sensitivity to azithromycin, rifampicin and vancomycin in *Acinetobacter baumannii*. Int. J. Antimicrob. Agents 46, 696–702. 10.1016/j.ijantimicag.2015.07.017 26391380

[B126] Garcia-Salguero C. Rodriguez-Avial I. Picazo J. J. Culebras E. (2015). Can plazomicin alone or in combination be a therapeutic pption against carbapenem-resistant *Acinetobacter baumannii*? Antimicrob. Agents Chemother. 59, 5959–5966. 10.1128/AAC.00873-15 26169398 PMC4576036

[B127] Gebhardt M. J. Gallagher L. A. Jacobson R. K. Usacheva E. A. Peterson L. R. Zurawski D. V.. (2015). Joint transcriptional control of virulence and resistance to antibiotic and environmental stress in *Acinetobacter baumannii*. MBio 6, e01660–15. 10.1128/mBio.01660-15 26556274 PMC4659468

[B128] Gehrlein M. Leying H. Cullmann W. Wendt S. Opferkuch W. (1991). Imipenem resistance in *Acinetobacter baumanii* is due to altered penicillin-binding proteins. Chemotherapy 37, 405–412. 1760939 10.1159/000238887

[B129] Geisinger E. Isberg R. R. (2015). Antibiotic modulation of capsular exopolysaccharide and virulence in *Acinetobacter baumannii*. PLoS Pathog. 11:e1004691. 10.1371/journal.ppat.1004691 25679516 PMC4334535

[B130] Giannouli M. Tomasone F. Agodi A. Vahaboglu H. Daoud Z. Triassi M.. (2009). Molecular epidemiology of carbapenem-resistant *Acinetobacter baumannii* strains in intensive care units of multiple Mediterranean hospitals. J. Antimicrob. Chemother. 63, 828–830. 10.1093/jac/dkp032 19223304

[B131] Gogou V. Pournaras S. Giannouli M. Voulgari E. Piperaki E. T. Zarrilli R.. (2011). Evolution of multidrug-resistant *Acinetobacter baumannii* clonal lineages: a 10 year study in Greece (2000–09). J. Antimicrob. Chemother. 66, 2767–2772. 10.1093/jac/dkr390 21933784

[B132] Goic-Barisic I. Towner K. J. Kovacic A. Sisko-Kraljevic K. Tonkic M. Novak A.. (2011). Outbreak in Croatia caused by a new carbapenem-resistant clone of *Acinetobacter baumannii* producing OXA-72 carbapenemase. J. Hosp. Infect. 77, 368–369. 10.1016/j.jhin.2010.12.003 21316806

[B133] Gonzalez-Villoria A. M. Tamayo-Legorreta E. Garza-Ramos U. Barrios H. Sanchez-Perez A. Rodriguez-Medina N.. (2016). A multicenter study in Mexico finds *Acinetobacter baumannii* clinical isolates belonging to clonal complexes 636B (113B) and 92B harboring OXA-72, OXA-239, and OXA-469. Antimicrob. Agents Chemother. 60, 2587–2588. 10.1128/AAC.02042-15 26833167 PMC4808170

[B134] Gordon N. C. Wareham D. W. (2009). A review of clinical and microbiological outcomes following treatment of infections involving multidrug-resistant *Acinetobacter baumannii* with tigecycline. J. Antimicrob. Chemother. 63, 775–780. 10.1093/jac/dkn555 19158109

[B135] Gordon N. C. Wareham D. W. (2010). Multidrug-resistant *Acinetobacter baumannii*: mechanisms of virulence and resistance. Int. J. Antimicrob. Agents 35, 219–226. 10.1016/j.ijantimicag.2009.10.024 20047818

[B136] Hagihara M. Housman S. T. Nicolau D. P. Kuti J. L. (2014). *In vitro* pharmacodynamics of polymyxin B and tigecycline alone and in combination against carbapenem-resistant *Acinetobacter baumannii*. Antimicrob. Agents Chemother. 58, 874–879. 10.1128/AAC.01624-13 24277022 PMC3910875

[B137] Hammerstrom T. G. Beabout K. Clements T. P. Saxer G. Shamoo Y. (2015). *Acinetobacter baumannii* repeatedly evolves a hypermutator phenotype in response to tigecycline that effectively surveys evolutionary trajectories to resistance. PLoS ONE 10:e0140489. 10.1371/journal.pone.0140489 26488727 PMC4619398

[B138] Hamouda A. Evans B. A. Towner K. J. Amyes S. G. (2010). Characterization of epidemiologically unrelated *Acinetobacter baumannii* isolates from four continents by use of multilocus sequence typing, pulsed-field gel electrophoresis, and sequence-based typing of *bla*_OXA-51*-like*_ genes. J. Clin. Microbiol. 48, 2476–2483. 10.1128/JCM.02431-09 20421437 PMC2897490

[B139] Harding C. M. Kinsella R. L. Palmer L. D. Skaar E. P. Feldman M. F. (2016). Medically relevant *Acinetobacter* species require a type II secretion system and specific membrane-associated chaperones for the export of multiple substrates and full virulence. PLoS Pathog. 12:e1005391. 10.1371/journal.ppat.1005391 26764912 PMC4713064

[B140] Hasani A. Sheikhalizadeh V. Ahangarzadeh Rezaee M. Rahmati-Yamchi M. Hasani A. Ghotaslou R.. (2016). Frequency of aminoglycoside-modifying enzymes and ArmA among different sequence groups of *Acinetobacter baumannii* in Iran. Microb. Drug Resist. 22, 347–353. 10.1089/mdr.2015.0254 26779992

[B141] Hassan A. Naz A. Obaid A. Paracha R. Z. Naz K. Awan F. M.. (2016). Pangenome and immuno-proteomics analysis of *Acinetobacter baumannii* strains revealed the core peptide vaccine targets. BMC Genomics. 17:732. 10.1186/s12864-016-2951-4 27634541 PMC5025611

[B142] Hawley J. S. Murray C. K. Jorgensen J. H. (2007). Development of colistin-dependent *Acinetobacter baumannii*-*Acinetobacter calcoaceticus* complex. Antimicrob. Agents Chemother. 51, 4529–4530. 10.1128/AAC.01115-07 17876007 PMC2168019

[B143] He S. He H. Chen Y. Chen Y. Wang W. Yu D. (2015). *In vitro* and *in vivo* analysis of antimicrobial agents alone and in combination against multi-drug resistant *Acinetobacter baumannii*. Front. Microbiol. 6, 507. 10.3389/fmicb.2015.00507 26074898 PMC4444844

[B144] He X. Lu F. Yuan F. Jiang D. Zhao P. Zhu J.. (2015). Biofilm formation caused by clinical *Acinetobacter baumannii* isolates is associated with overexpression of the AdeFGH efflux pump. Antimicrob. Agents Chemother. 59, 4817–4825. 10.1128/AAC.00877-15 26033730 PMC4505227

[B145] Heritier C. Poirel L. Aubert D. Nordmann P. (2003). Genetic and functional analysis of the chromosome-encoded carbapenem-hydrolyzing oxacillinase OXA-40 of *Acinetobacter baumannii*. Antimicrob. Agents Chemother. 47, 268–273. 10.1128/AAC.47.1.268-273.2003 12499201 PMC149012

[B146] Heritier C. Poirel L. Fournier P. E. Claverie J. M. Raoult D. Nordmann P. (2005a). Characterization of the naturally occurring oxacillinase of *Acinetobacter baumannii*. Antimicrob. Agents Chemother. 49, 4174–4179. 10.1128/AAC.49.10.4174-4179.2005 16189095 PMC1251506

[B147] Heritier C. Poirel L. Lambert T. Nordmann P. (2005b). Contribution of acquired carbapenem-hydrolyzing oxacillinases to carbapenem resistance in *Acinetobacter baumannii*. Antimicrob. Agents Chemother. 49, 3198–3202. 10.1128/AAC.49.8.3198-3202.2005 16048925 PMC1196226

[B148] Heritier C. Poirel L. Nordmann P. (2006). Cephalosporinase over-expression resulting from insertion of IS*Aba1* in *Acinetobacter baumannii*. Clin. Microbiol. Infect. 12, 123–130. 10.1111/j.1469-0691.2005.01320.x 16441449

[B149] Higgins P. G. Janssen K. Fresen M. M. Wisplinghoff H. Seifert H. (2012). Molecular epidemiology of *Acinetobacter baumannii* bloodstream isolates obtained in the United States from 1995 to 2004 using rep-PCR and multilocus sequence typing. J. Clin. Microbiol. 50, 3493–3500. 10.1128/JCM.01759-12 22895032 PMC3486219

[B150] Higgins P. G. Perez-Llarena F. J. Zander E. Fernandez A. Bou G. Seifert H. (2013). OXA-235, a novel class D β-lactamase involved in resistance to carbapenems in *Acinetobacter baumannii*. Antimicrob. Agents Chemother. 57, 2121–2126. 10.1128/AAC.02413-12 23439638 PMC3632948

[B151] Higgins P. G. Poirel L. Lehmann M. Nordmann P. Seifert H. (2009). OXA-143, a novel carbapenem-hydrolyzing class D β-lactamase in *Acinetobacter baumannii*. Antimicrob. Agents Chemother. 53, 5035–5038. 10.1128/AAC.00856-09 19770279 PMC2786334

[B152] Higgins P. G. Wisplinghoff H. Stefanik D. Seifert H. (2004). Selection of topoisomerase mutations and overexpression of *adeB* mRNA transcripts during an outbreak of *Acinetobacter baumannii*. J. Antimicrob. Chemother. 54, 821–823. 10.1093/jac/dkh427 15355942

[B153] Hiraki Y. Yoshida M. Masuda Y. Inoue D. Tsuji Y. Kamimura H.. (2013). Successful treatment of skin and soft tissue infection due to carbapenem-resistant *Acinetobacter baumannii* by ampicillin-sulbactam and meropenem combination therapy. Int. J. Infect. Dis. 17, e1234–e1236. 10.1016/j.ijid.2013.05.002 23791858

[B154] Hogg G. M. Barr J. G. Webb C. H. (1998). *In-vitro* activity of the combination of colistin and rifampicin against multidrug-resistant strains of *Acinetobacter baumannii*. J Antimicrob Chemother. 41, 494–495. 9598783 10.1093/jac/41.4.494

[B155] Hong S. B. Shin K. S. Ha J. Han K. (2013). Co-existence of *bla*_OXA-23_ and *armA* in multidrug-resistant *Acinetobacter baumannii* isolated from a hospital in South Korea. J. Med. Microbiol. 62, 836–844. 10.1099/jmm.0.055384-0 23518656

[B156] Hong Y. K. Lee J. Y. Wi Y. M. Ko K. S. (2016). High rate of colistin dependence in *Acinetobacter baumannii*. J. Antimicrob. Chemother. 71, 2346–2348. 10.1093/jac/dkw121 27076109

[B157] Hood M. I. Jacobs A. C. Sayood K. Dunman P. M. Skaar E. P. (2010). *Acinetobacter baumannii* increases tolerance to antibiotics in response to monovalent cations. Antimicrob. Agents Chemother. 54, 1029–1041. 10.1128/AAC.00963-09 20028819 PMC2825970

[B158] Hood M. I. Mortensen B. L. Moore J. L. Zhang Y. Kehl-Fie T. E. Sugitani N.. (2012). Identification of an *Acinetobacter baumannii* zinc acquisition system that facilitates resistance to calprotectin-mediated zinc sequestration. PLoS Pathog. 8:e1003068. 10.1371/journal.ppat.1003068 23236280 PMC3516566

[B159] Hornsey M. Ellington M. J. Doumith M. Thomas C. P. Gordon N. C. Wareham D. W.. (2010). AdeABC-mediated efflux and tigecycline MICs for epidemic clones of *Acinetobacter baumannii*. J. Antimicrob. Chemother. 65, 1589–1593. 10.1093/jac/dkq218 20554571

[B160] Hornsey M. Loman N. Wareham D. W. Ellington M. J. Pallen M. J. Turton J. F.. (2011). Whole-genome comparison of two *Acinetobacter baumannii* isolates from a single patient, where resistance developed during tigecycline therapy. J. Antimicrob. Chemother. 66, 1499–1503. 10.1093/jac/dkr168 21565804

[B161] Hornsey M. Wareham D. W. (2011). *In vivo* efficacy of glycopeptide-colistin combination therapies in a *Galleria mellonella* model of *Acinetobacter baumannii* infection. Antimicrob. Agents Chemother. 55, 3534–3537. 10.1128/AAC.00230-11 21502628 PMC3122470

[B162] Hou C. Yang F. (2015). Drug-resistant gene of *bla*_OXA-23_, *bla*_OXA-24_, *bla*_OXA-51_and *bla*_OXA-58_ in *Acinetobacter baumannii*. Int. J. Clin. Exp. Med. 8, 13859–13863. 26550338 PMC4613023

[B163] Houang E. T. Chu Y. W. Chu K. Y. Ng K. C. Leung C. M. Cheng A. F. (2003). Significance of genomic DNA group delineation in comparative studies of antimicrobial susceptibility of *Acinetobacter* spp. Antimicrob. Agents Chemother. 47, 1472–1475. 10.1128/AAC.47.4.1472-1475.2003 12654697 PMC152486

[B164] Hu D. Liu B. Dijkshoorn L. Wang L. Reeves P. R. (2013). Diversity in the major polysaccharide antigen of *Acinetobacter baumannii* assessed by DNA sequencing, and development of a molecular serotyping scheme. PLoS ONE 8:e70329. 10.1371/journal.pone.0070329 23922982 PMC3726653

[B165] Hu W. S. Yao S. M. Fung C. P. Hsieh Y. P. Liu C. P. Lin J. F. (2007). An OXA-66/OXA-51-like carbapenemase and possibly an efflux pump are associated with resistance to imipenem in *Acinetobacter baumannii*. Antimicrob. Agents Chemother. 51, 3844–3852. 10.1128/AAC.01512-06 17724156 PMC2151406

[B166] Huang W. Yao Y. Long Q. Yang X. Sun W. Liu C.. (2014). Immunization against multidrug-resistant *Acinetobacter baumannii* effectively protects mice in both pneumonia and sepsis models. PLoS ONE 9:e100727. 10.1371/journal.pone.0100727 24956279 PMC4067354

[B167] Huang W. Yao Y. Wang S. Xia Y. Yang X. Long Q.. (2016). Immunization with a 22-kDa outer membrane protein elicits protective immunity to multidrug-resistant *Acinetobacter baumannii*. Sci. Rep. 6:20724. 10.1038/srep20724 26853590 PMC4745112

[B168] Hujer K. M. Hamza N. S. Hujer A. M. Perez F. Helfand M. S. Bethel C. R.. (2005). Identification of a new allelic variant of the *Acinetobacter baumannii* cephalosporinase, ADC-7 β-lactamase: defining a unique family of class C enzymes. Antimicrob. Agents Chemother. 49, 2941–2948. 10.1128/AAC.49.7.2941-2948.2005 15980372 PMC1168656

[B169] Iwashkiw J. A. Seper A. Weber B. S. Scott N. E. Vinogradov E. Stratilo C.. (2012). Identification of a general O-linked protein glycosylation system in *Acinetobacter baumannii* and its role in virulence and biofilm formation. PLoS Pathog. 8:e1002758. 10.1371/journal.ppat.1002758 22685409 PMC3369928

[B170] Jacobs A. C. Hood I. Boyd K. L. Olson P. D. Morrison J. M. Carson S.. (2010). Inactivation of phospholipase D diminishes *Acinetobacter baumannii* pathogenesis. Infect. Immun. 78, 1952–1962. 10.1128/IAI.00889-09 20194595 PMC2863507

[B171] Jara L. M. Perez-Varela M. Corral J. Arch M. Cortes P. Bou G.. (2015). Novobiocin inhibits the antimicrobial resistance acquired through DNA damage-induced mutagenesis in *Acinetobacter baumannii*. Antimicrob. Agents Chemother. 60, 637–639. 10.1128/AAC.01810-15 26503651 PMC4704144

[B172] Jaruratanasirikul S. Wongpoowarak W. Wattanavijitkul T. Sukarnjanaset W. Samaeng M. Nawakitrangsan M.. (2016). Population pharmacokinetics and pharmacodynamics modeling to optimize dosage regimens of sulbactam in critically ill patients with severe sepsis caused by *Acinetobacter baumannii*. Antimicrob. Agents Chemother. 60, 7236–7244. 10.1128/AAC.01669-16 27671056 PMC5119003

[B173] Jeon J. H. Lee J. H. Lee J. J. Park K. S. Karim A. M. Lee C. R.. (2015). Structural basis for carbapenem-hydrolyzing mechanisms of carbapenemases conferring antibiotic resistance. Int. J. Mol. Sci. 16, 9654–9692. 10.3390/ijms16059654 25938965 PMC4463611

[B174] Jeon J. Ryu C. M. Lee J. Y. Park J. H. Yong D. Lee K. (2016). *In vivo* application of bacteriophage as a potential therapeutic agent to control OXA-66-like carbapenemase-producing *Acinetobacter baumannii* strains belonging to sequence type 357. Appl. Environ. Microbiol. 82, 4200–4208. 10.1128/AEM.00526-16 27208124 PMC4959215

[B175] Jeong H. W. Cheong H. J. Kim W. J. Kim M. J. Song K. J. Song J. W.. (2009). Loss of the 29-kilodalton outer membrane protein in the presence of OXA-51-like enzymes in *Acinetobacter baumannii* is associated with decreased imipenem susceptibility. Microb. Drug Resist. 15, 151–158. 10.1089/mdr.2009.0828 19728771

[B176] Jeong S. H. Bae I. K. Kwon S. B. Lee K. Yong D. Woo G. J.. (2005). Investigation of a nosocomial outbreak of *Acinetobacter baumannii* producing PER-1 extended-spectrum β-lactamase in an intensive care unit. J. Hosp. Infect. 59, 242–248. 10.1016/j.jhin.2004.09.025 15694982

[B177] Jin J. S. Kwon S. O. Moon D. C. Gurung M. Lee J. H. Kim S. I.. (2011). *Acinetobacter baumannii* secretes cytotoxic outer membrane protein A via outer membrane vesicles. PLoS ONE 6:e17027. 10.1371/journal.pone.0017027 21386968 PMC3046175

[B178] Johnson T. L. Waack U. Smith S. Mobley H. Sandkvist M. (2015). *Acinetobacter baumannii* is dependent on the type II secretion system and its substrate LipA for lipid utilization and *in vivo* fitness. J. Bacteriol. 198, 711–719. 10.1128/JB.00622-15 26668261 PMC4751819

[B179] Jones C. L. Clancy M. Honnold C. Singh S. Snesrud E. Onmus-Leone F.. (2015). Fatal outbreak of an emerging clone of extensively drug-resistant *Acinetobacter baumannii* with enhanced virulence. Clin. Infect. Dis. 61, 145–154. 10.1093/cid/civ225 25824815

[B180] Jun S. H. Lee J. H. Kim B. R. Kim S. I. Park T. I. Lee J. C.. (2013). *Acinetobacter baumannii* outer membrane vesicles elicit a potent innate immune response via membrane proteins. PLoS ONE 8:e71751. 10.1371/journal.pone.0071751 23977136 PMC3743744

[B181] Juttukonda L. J. Chazin W. J. Skaar E. P. (2016). *Acinetobacter baumannii* coordinates urea metabolism with metal import to resist host-mediated metal limitation. MBio 7:e01475–16. 10.1128/mBio.01475-16 27677795 PMC5050338

[B182] Kalin G. Alp E. Akin A. Coskun R. Doganay M. (2014). Comparison of colistin and colistin/sulbactam for the treatment of multidrug resistant *Acinetobacter baumannii* ventilator-associated pneumonia. Infection. 42, 37–42. 10.1007/s15010-013-0495-y 23828559

[B183] Kaplan J. B. (2011). Antibiotic-induced biofilm formation. Int. J. Artif. Organs. 34, 737–751. 10.5301/ijao.5000027 22094552

[B184] Karthikeyan K. Thirunarayan M. A. Krishnan P. (2010). Coexistence of *bla*_OXA-23_ with *bla*_NDM-1_ and *armA* in clinical isolates of *Acinetobacter baumannii* from India. J. Antimicrob. Chemother. 65, 2253–2254. 10.1093/jac/dkq273 20650909

[B185] Kenyon J. J. Hall R. M. (2013). Variation in the complex carbohydrate biosynthesis loci of *Acinetobacter baumannii* genomes. PLoS ONE 8:e62160. 10.1371/journal.pone.0062160 23614028 PMC3628348

[B186] Kim C. K. Lee Y. Lee H. Woo G. J. Song W. Kim M. N.. (2010). Prevalence and diversity of carbapenemases among imipenem-nonsusceptible *Acinetobacter* isolates in Korea: emergence of a novel OXA-182. Diagn. Microbiol. Infect. Dis. 68, 432–438. 10.1016/j.diagmicrobio.2010.07.014 20884158

[B187] Kim S. W. Choi C. H. Moon D. C. Jin J. S. Lee J. H. Shin J. H.. (2009). Serum resistance of *Acinetobacter baumannii* through the binding of factor H to outer membrane proteins. FEMS Microbiol. Lett. 301, 224–231. 10.1111/j.1574-6968.2009.01820.x 19878322

[B188] Kim Y. J. Kim S. I. Kim Y. R. Hong K. W. Wie S. H. Park Y. J.. (2012). Carbapenem-resistant *Acinetobacter baumannii*: diversity of resistant mechanisms and risk factors for infection. Epidemiol. Infect. 140, 137–145. 10.1017/S0950268811000744 21554783

[B189] Knight D. Dimitrova D. D. Rudin S. D. Bonomo R. A. Rather P. N. (2016). Mutations decreasing intrinsic β-lactam resistance are linked to cell division in the nosocomial pathogen *Acinetobacter baumannii*. Antimicrob. Agents Chemother. 60, 3751–3758. 10.1128/AAC.00361-16 27067318 PMC4879375

[B190] Kobayashi N. Nishino K. Yamaguchi A. (2001). Novel macrolide-specific ABC-type efflux transporter in *Escherichia coli*. J. Bacteriol. 183, 5639–5644. 10.1128/JB.183.19.5639-5644.2001 11544226 PMC95455

[B191] Koeleman J. G. Stoof J. Biesmans D. J. Savelkoul P. H. Vandenbroucke-Grauls C. M. (1998). Comparison of amplified ribosomal DNA restriction analysis, random amplified polymorphic DNA analysis, and amplified fragment length polymorphism fingerprinting for identification of *Acinetobacter* genomic species and typing of *Acinetobacter baumannii*. J. Clin. Microbiol. 36, 2522–2529. 9705386 10.1128/jcm.36.9.2522-2529.1998 PMC105156

[B192] Koeleman J. G. van der Bijl M. W. Stoof J. Vandenbroucke-Grauls C. M. Savelkoul P. H. (2001). Antibiotic resistance is a major risk factor for epidemic behavior of *Acinetobacter baumannii*. Infect. Control Hosp Epidemiol. 22, 284–288. 10.1086/501901 11428438

[B193] Koenigs A. Stahl J. Averhoff B. Gottig S. Wichelhaus T. A. Wallich R.. (2016). CipA of *Acinetobacter baumannii* is a novel plasminogen binding and complement inhibitory protein. J. Infect. Dis. 213, 1388–1399. 10.1093/infdis/jiv601 26681776

[B194] Koenigs A. Zipfel P. F. Kraiczy P. (2015). Translation elongation factor Tuf of *Acinetobacter baumannii* is a plasminogen-binding protein. PLoS ONE 10:e0134418. 10.1371/journal.pone.0134418 26230848 PMC4521846

[B195] Koh T. H. Sng L. H. Wang G. C. Hsu L. Y. Zhao Y. (2007). IMP-4 and OXA beta-lactamases in *Acinetobacter baumannii* from Singapore. J. Antimicrob. Chemother. 59, 627–632. 10.1093/jac/dkl544 17284537

[B196] Koh T. H. Tan T. T. Khoo C. T. Ng S. Y. Tan T. Y. Hsu L. Y.. (2012). *Acinetobacter calcoaceticus*-*Acinetobacter baumannii* complex species in clinical specimens in Singapore. Epidemiol. Infect. 140, 535–538. 10.1017/S0950268811001129 21733253

[B197] Kohlenberg A. Brummer S. Higgins P. G. Sohr D. Piening B. C. de Grahl C.. (2009). Outbreak of carbapenem-resistant *Acinetobacter baumannii* carrying the carbapenemase OXA-23 in a German university medical centre. J. Med. Microbiol. 58, 1499–1507. 10.1099/jmm.0.012302-0 19589905

[B198] Konstantinidis T. Kambas K. Mitsios A. Panopoulou M. Tsironidou V. Dellaporta E.. (2016). Immunomodulatory role of clarithromycin in *Acinetobacter baumannii* infection via formation of neutrophil extracellular traps. Antimicrob. Agents Chemother. 60, 1040–1048. 10.1128/AAC.02063-15 26643338 PMC4750671

[B199] Koomanachai P. Kim A. Nicolau D. P. (2009). Pharmacodynamic evaluation of tigecycline against *Acinetobacter baumannii* in a murine pneumonia model. J. Antimicrob. Chemother. 63, 982–987. 10.1093/jac/dkp056 19279050

[B200] Korotkov K. V. Sandkvist M. Hol W. G. (2012). The type II secretion system: biogenesis, molecular architecture and mechanism. Nat. Rev. Microbiol. 10, 336–351. 10.1038/nrmicro2762 22466878 PMC3705712

[B201] Kostoulias X. Murray G. L. Cerqueira G. M. Kong J. B. Bantun F. Mylonakis E.. (2015). Impact of a cross-kingdom signaling molecule of *Candida albicans* on *Acinetobacter baumannii* physiology. Antimicrob. Agents Chemother. 60, 161–167. 10.1128/AAC.01540-15 26482299 PMC4704244

[B202] Krizova L. Poirel L. Nordmann P. Nemec A. (2013). TEM-1 β-lactamase as a source of resistance to sulbactam in clinical strains of *Acinetobacter baumannii*. J. Antimicrob. Chemother. 68, 2786–2791. 10.1093/jac/dkt275 23838947

[B203] Kulp A. Kuehn M. J. (2010). Biological functions and biogenesis of secreted bacterial outer membrane vesicles. Annu. Rev. Microbiol. 64, 163–184. 10.1146/annurev.micro.091208.073413 20825345 PMC3525469

[B204] Kumar M. (2016). Identification of a novel NDM variant, *bla*_*NDM*−3_, from a multidrug-resistant *Acinetobacter baumannii*. Infect. Control. Hosp. Epidemiol. 37, 747–748. 10.1017/ice.2016.66 27071840

[B205] Kuo H. Y. Hsu P. J. Chen J. Y. Liao P. C. Lu C. W. Chen C. H.. (2016). Clonal spread of *bla*_OXA-72_-carrying *Acinetobacter baumannii* sequence type 512 in Taiwan. Int. J. Antimicrob. Agents 48, 111–113. 10.1016/j.ijantimicag.2016.04.020 27242318

[B206] Kuo H. Y. Yang C. M. Lin M. F. Cheng W. L. Tien N. Liou M. L. (2010). Distribution of *bla*_OXA-*carrying*_ imipenem-resistant *Acinetobacter* spp. in 3 hospitals in Taiwan. Diagn. Microbiol. Infect. Dis. 66, 195–199. 10.1016/j.diagmicrobio.2009.09.013 19836186

[B207] Kuo L. C. Lai C. C. Liao C. H. Hsu C. K. Chang Y. L. Chang C. Y.. (2007). Multidrug-resistant *Acinetobacter baumannii* bacteraemia: clinical features, antimicrobial therapy and outcome. Clin. Microbiol. Infect. 13, 196–198. 10.1111/j.1469-0691.2006.01601.x 17328733

[B208] Kuo S. C. Chang S. C. Wang H. Y. Lai J. F. Chen P. C. Shiau Y. R.. (2012). Emergence of extensively drug-resistant *Acinetobacter baumannii* complex over 10 years: nationwide data from the Taiwan Surveillance of Antimicrobial Resistance (TSAR) program. BMC Infect Dis. 12:200. 10.1186/1471-2334-12-200 22929085 PMC3462144

[B209] Kusradze I. Karumidze N. Rigvava S. Dvalidze T. Katsitadze M. Amiranashvili I.. (2016). Characterization and testing the efficiency of *Acinetobacter baumannii* phage *vB-GEC_Ab-M-G7* as an antibacterial agent. Front. Microbiol. 7:1590. 10.3389/fmicb.2016.01590 27757110 PMC5047890

[B210] Kwon S. O. Gho Y. S. Lee J. C. Kim S. I. (2009). Proteome analysis of outer membrane vesicles from a clinical *Acinetobacter baumannii* isolate. FEMS Microbiol. Lett. 297, 150–156. 10.1111/j.1574-6968.2009.01669.x 19548894

[B211] La Scola B. Gundi V. A. Khamis A. Raoult D. (2006). Sequencing of the *rpoB* gene and flanking spacers for molecular identification of *Acinetobacter* species. J. Clin. Microbiol. 44, 827–832. 10.1128/JCM.44.3.827-832.2006 16517861 PMC1393131

[B212] Lee C. H. Tang Y. F. Su L. H. Chien C. C. Liu J. W. (2008). Antimicrobial effects of varied combinations of meropenem, sulbactam, and colistin on a multidrug-resistant *Acinetobacter baumannii* isolate that caused meningitis and bacteremia. Microb. Drug Resist. 14, 233–237. 10.1089/mdr.2008.0840 18707240

[B213] Lee C. R. Cho I. H. Jeong B. C. Lee S. H. (2013a). Strategies to minimize antibiotic resistance. Int. J. Environ. Res. Public Health. 10, 4274–4305. 10.3390/ijerph10094274 24036486 PMC3799537

[B214] Lee C. R. Lee J. H. Jeong B. C. Lee S. H. (2013b). Lipid a biosynthesis of multidrug-resistant pathogens - a novel drug target. Curr. Pharm. Des. 19, 6534–6550. 10.2174/13816128113199990494 23829374

[B215] Lee C. R. Lee J. H. Park K. S. Jeong B. C. Lee S. H. (2015). Quantitative proteomic view associated with resistance to clinically important antibiotics in Gram-positive bacteria: a systematic review. Front. Microbiol. 6:828. 10.3389/fmicb.2015.00828 26322035 PMC4531251

[B216] Lee C. R. Lee J. H. Park K. S. Kim Y. B. Jeong B. C. Lee S. H. (2016). Global Dissemination of Carbapenemase-Producing *Klebsiella pneumoniae*: epidemiology, Genetic Context, Treatment Options, and Detection Methods. Front. Microbiol. 7:895. 10.3389/fmicb.2016.00895 27379038 PMC4904035

[B217] Lee J. H. Choi C. H. Kang H. Y. Lee J. Y. Kim J. Lee Y. C.. (2007). Differences in phenotypic and genotypic traits against antimicrobial agents between *Acinetobacter baumannii* and *Acinetobacter* genomic species 13TU. J. Antimicrob. Chemother. 59, 633–639. 10.1093/jac/dkm007 17339277

[B218] Lee J. H. Lee J. J. Park K. S. Lee S. H. (2015). Urgent need for β-lactam-β-lactamase inhibitors. Lancet Infect. Dis. 15, 876–877. 10.1016/S1473-3099(15)00143-7 26227756

[B219] Lee J. H. Park K. S. Karim A. M. Lee C. R. Lee S. H. (2016). How to minimise antibiotic resistance. Lancet Infect. Dis. 16, 17–18. 10.1016/S1473-3099(15)00467-3 26738826

[B220] Lee J. S. Choi C. H. Kim J. W. Lee J. C. (2010). *Acinetobacter baumannii* outer membrane protein A induces dendritic cell death through mitochondrial targeting. J. Microbiol. 48, 387–392. 10.1007/s12275-010-0155-1 20571958

[B221] Lee K. Yum J. H. Yong D. Lee H. M. Kim H. D. Docquier J. D.. (2005). Novel acquired metallo-β-lactamase gene, *bla*_*SIM-*1_, in a class 1 integron from *Acinetobacter baumannii* clinical isolates from Korea. Antimicrob. Agents Chemother. 49, 4485–4491. 10.1128/AAC.49.11.4485-4491.2005 16251286 PMC1280121

[B222] Lee M. F. Peng C. F. Hsu H. J. Chen Y. H. (2008). Molecular characterisation of the metallo-β-lactamase genes in imipenem-resistant Gram-negative bacteria from a university hospital in southern Taiwan. Int. J. Antimicrob. Agents 32, 475–480. 10.1016/j.ijantimicag.2008.07.009 18804966

[B223] Lee Y. Kim C. K. Lee H. Jeong S. H. Yong D. Lee K. (2011). A novel insertion sequence, IS*Aba10*, inserted into ISAba1 adjacent to the *bla*_OXA-23_ gene and disrupting the outer membrane protein gene *carO* in *Acinetobacter baumannii*. Antimicrob. Agents Chemother. 55, 361–363. 10.1128/AAC.01672-09 20937784 PMC3019662

[B224] Lee Y. T. Tsao S. M. Hsueh P. R. (2013). Clinical outcomes of tigecycline alone or in combination with other antimicrobial agents for the treatment of patients with healthcare-associated multidrug-resistant *Acinetobacter baumannii* infections. Eur. J. Clin. Microbiol Infect Dis. 32, 1211–1220. 10.1007/s10096-013-1870-4 23553594

[B225] Lees-Miller R. G. Iwashkiw J. A. Scott N. E. Seper A. Vinogradov E. Schild S.. (2013). A common pathway for O-linked protein-glycosylation and synthesis of capsule in *Acinetobacter baumannii*. Mol. Microbiol. 89, 816–830. 10.1111/mmi.12300 23782391

[B226] Lenhard J. R. Bulitta J. B. Connell T. D. King-Lyons N. Landersdorfer C. B. Cheah S. E.. (2016a). High-intensity meropenem combinations with polymyxin B: new strategies to overcome carbapenem resistance in *Acinetobacter baumannii*. J. Antimicrob. Chemother. 72, 153–165. 10.1093/jac/dkw355 27634916 PMC5161044

[B227] Lenhard J. R. Gall J. S. Bulitta J. B. Thamlikitkul V. Landersdorfer C. B. Forrest A.. (2016b). Comparative pharmacodynamics of four different carbapenems in combination with polymyxin B against carbapenem-resistant *Acinetobacter baumannii*. Int. J. Antimicrob. Agents 48, 719–724. 10.1016/j.ijantimicag.2016.07.024 27773498 PMC5237376

[B228] Li L. Hassan K. A. Brown M. H. Paulsen I. T. (2016). Rapid multiplexed phenotypic screening identifies drug resistance functions for three novel efflux pumps in *Acinetobacter baumannii*. J. Antimicrob. Chemother. 71, 1223–1232. 10.1093/jac/dkv460 26832750

[B229] Li X. Liu L. Ji J. Chen Q. Hua X. Jiang Y.. (2015). Tigecycline resistance in *Acinetobacter baumannii* mediated by frameshift mutation in *plsC*, encoding 1-acyl-*sn*-glycerol-3-phosphate acyltransferase. Eur. J. Clin. Microbiol. Infect. Dis. 34, 625–631. 10.1007/s10096-014-2272-y 25407371

[B230] Li X. M. Choi J. A. Choi I. S. Kook J. K. Chang Y. H. Park G.. (2016). Development and evaluation of species-specific PCR for detection of nine *Acinetobacter* species. Ann. Clin. Lab. Sci. 46, 270–278. 27312551

[B231] Li X. Quan J. Yang Y. Ji J. Liu L. Fu Y.. (2016). Abrp, a new gene, confers reduced susceptibility to tetracycline, glycylcine, chloramphenicol and fosfomycin classes in *Acinetobacter baumannii*. Eur. J. Clin. Microbiol. Infect. Dis. 35, 1371–1375. 10.1007/s10096-016-2674-0 27220329

[B232] Li Y. Guo Q. Wang P. Zhu D. Ye X. Wu S.. (2015). Clonal dissemination of extensively drug-resistant *Acinetobacter baumannii* producing an OXA-23 β-lactamase at a teaching hospital in Shanghai, China. J. Microbiol. Immunol. Infect. 48, 101–108. 10.1016/j.jmii.2014.04.005 24863499

[B233] Li Z. T. Zhang R. L. Bi X. G. Xu L. Fan M. Xie D.. (2015). Outer membrane vesicles isolated from two clinical *Acinetobacter baumannii* strains exhibit different toxicity and proteome characteristics. Microb. Pathog. 81, 46–52. 10.1016/j.micpath.2015.03.009 25773772

[B234] Liang W. Liu X. F. Huang J. Zhu D. M. Li J. Zhang J. (2011). Activities of colistin- and minocycline-based combinations against extensive drug resistant *Acinetobacter baumannii* isolates from intensive care unit patients. BMC Infect. Dis. 11:109. 10.1186/1471-2334-11-109 21521536 PMC3098177

[B235] Liao Y. T. Kuo S. C. Chiang M. H. Lee Y. T. Sung W. C. Chen Y. H.. (2015). *Acinetobacter baumannii* extracellular OXA-58 is primarily and selectively released via outer membrane vesicles after Sec-dependent periplasmic translocation. Antimicrob. Agents Chemother. 59, 7346–7354. 10.1128/AAC.01343-15 26369971 PMC4649246

[B236] Lin L. Tan B. Pantapalangkoor P. Ho T. Baquir B. Tomaras A.. (2012). Inhibition of LpxC protects mice from resistant *Acinetobacter baumannii* by modulating inflammation and enhancing phagocytosis. MBio 3:e00312–12. 10.1128/mBio.00312-12 23033474 PMC3518917

[B237] Lin L. Tan B. Pantapalangkoor P. Ho T. Hujer A. M. Taracila M. A.. (2013). *Acinetobacter baumannii* rOmpA vaccine dose alters immune polarization and immunodominant epitopes. Vaccine 31, 313–318. 10.1016/j.vaccine.2012.11.008 23153442 PMC3557524

[B238] Lin M. F. Chang K. C. Lan C. Y. Chou J. Kuo J. W. Chang C. K.. (2011a). Molecular epidemiology and antimicrobial resistance determinants of multidrug-resistant *Acinetobacter baumannii i*n five proximal hospitals in Taiwan. Jpn. J. Infect. Dis. 64, 222–227. 21617307

[B239] Lin M. F. Chang K. C. Yang C. Y. Yang C. M. Xiao C. C. Kuo H. Y.. (2010). Role of integrons in antimicrobial susceptibility patterns of *Acinetobacter baumannii*. Jpn. J. Infect. Dis. 63, 440–443. 21099097

[B240] Lin M. F. Kuo H. Y. Yeh H. W. Yang C. M. Sung C. H. Tu C. C.. (2011b). Emergence and dissemination of *bla*_OXA-23_-carrying imipenem-resistant *Acinetobacter* sp. in a regional hospital in Taiwan. J. Microbiol. Immunol. Infect. 44, 39–44. 10.1016/j.jmii.2011.01.008 21531351

[B241] Lin M. F. Lan C. Y. (2014). Antimicrobial resistance in Acinetobacter baumannii: from bench to bedside. World J. Clin. Cases. 2, 787–814. 10.12998/wjcc.v2.i12.787 25516853 PMC4266826

[B242] Lin M. F. Lin Y. Y. Lan C. Y. (2015). The role of the two-component system BaeSR in disposing chemicals through regulating transporter systems in *Acinetobacter baumannii*. PLoS ONE 10:e0132843. 10.1371/journal.pone.0132843 26161744 PMC4498774

[B243] Lin M. F. Lin Y. Y. Yeh H. W. Lan C. Y. (2014). Role of the BaeSR two-component system in the regulation of *Acinetobacter baumannii adeAB* genes and its correlation with tigecycline susceptibility. BMC Microbiol. 14:119. 10.1186/1471-2180-14-119 24885279 PMC4101873

[B244] Lin M. F. Liou M. L. Tu C. C. Yeh H. W. Lan C. Y. (2013). Molecular epidemiology of integron-associated antimicrobial gene cassettes in the clinical isolates of *Acinetobacter baumannii* from northern Taiwan. Ann. Lab. Med. 33, 242–247. 10.3343/alm.2013.33.4.242 23826559 PMC3698301

[B245] Liou M. L. Soo P. C. Ling S. R. Kuo H. Y. Tang C. Y. Chang K. C. (2014). The sensor kinase BfmS mediates virulence in *Acinetobacter baumannii*. J. Microbiol. Immunol. Infect. 47, 275–281. 10.1016/j.jmii.2012.12.004 23453128

[B246] Liu C. C. Kuo H. Y. Tang C. Y. Chang K. C. Liou M. L. (2014). Prevalence and mapping of a plasmid encoding a type IV secretion system in *Acinetobacter baumannii*. Genomics. 104, 215–223. 10.1016/j.ygeno.2014.07.011 25072866

[B247] Liu D. Liu Z. S. Hu P. Cai L. Fu B. Q. Li Y. S.. (2016). Characterization of surface antigen protein 1 (SurA1) from *Acinetobacter baumannii* and its role in virulence and fitness. Vet. Microbiol. 186, 126–138. 10.1016/j.vetmic.2016.02.018 27016767

[B248] Liu Q. Li W. Feng Y. Tao C. (2014). Efficacy and safety of polymyxins for the treatment of *Acinectobacter baumannii* infection: a systematic review and meta-analysis. PLoS ONE 9:e98091. 10.1371/journal.pone.0098091 24911658 PMC4049575

[B249] Liu X. Zhao M. Chen Y. Bian X. Li Y. Shi J.. (2016). Synergistic killing by meropenem and colistin combination of carbapenem-resistant *Acinetobacter baumannii* isolates from Chinese patients in an *in vitro* pharmacokinetic/pharmacodynamic model. Int. J. Antimicrob. Agents 48, 559–563. 10.1016/j.ijantimicag.2016.07.018 27670371

[B250] Liu Y. Liu X. (2015). Detection of AmpC β-lactamases in *Acinetobacter baumannii* in the Xuzhou region and analysis of drug resistance. Exp. Ther. Med. 10, 933–936. 10.3892/etm.2015.2612 26622417 PMC4533231

[B251] Lolans K. Rice T. W. Munoz-Price L. S. Quinn J. P. (2006). Multicity outbreak of carbapenem-resistant *Acinetobacter baumannii* isolates producing the carbapenemase OXA-40. Antimicrob. Agents Chemother. 50, 2941–2945. 10.1128/AAC.00116-06 16940085 PMC1563549

[B252] Lomovskaya O. Lewis K. (1992). Emr, an *Escherichia coli* locus for multidrug resistance. Proc. Natl. Acad. Sci. U.S.A. 89, 8938–8942. 1409590 10.1073/pnas.89.19.8938 PMC50039

[B253] Luke N. R. Sauberan S. L. Russo T. A. Beanan J. M. Olson R. Loehfelm T. W.. (2010). Identification and characterization of a glycosyltransferase involved in *Acinetobacter baumannii* lipopolysaccharide core biosynthesis. Infect. Immun. 78, 2017–2023. 10.1128/IAI.00016-10 20194587 PMC2863528

[B254] Luo G. Lin L. Ibrahim A. S. Baquir B. Pantapalangkoor P. Bonomo R. A.. (2012). Active and passive immunization protects against lethal, extreme drug resistant-*Acinetobacter baumannii* infection. PLoS ONE 7:e29446. 10.1371/journal.pone.0029446 22253723 PMC3254619

[B255] Lu P. L. Doumith M. Livermore D. M. Chen T. P. Woodford N. (2009). Diversity of carbapenem resistance mechanisms in *Acinetobacter baumannii* from a Taiwan hospital: spread of plasmid-borne OXA-72 carbapenemase. J. Antimicrob. Chemother. 63, 641–647. 10.1093/jac/dkn553 19182237

[B256] Magnet S. Courvalin P. Lambert T. (2001). Resistance-nodulation-cell division-type efflux pump involved in aminoglycoside resistance in *Acinetobacter baumannii* strain BM4454. Antimicrob. Agents Chemother. 45, 3375–3380. 10.1128/AAC.45.12.3375-3380.2001 11709311 PMC90840

[B257] Mak J. K. Kim M. J. Pham J. Tapsall J. White P. A. (2009). Antibiotic resistance determinants in nosocomial strains of multidrug-resistant *Acinetobacter baumannii*. J. Antimicrob. Chemother. 63, 47–54. 10.1093/jac/dkn454 18988680

[B258] Marchand I. Damier-Piolle L. Courvalin P. Lambert T. (2004). Expression of the RND-type efflux pump AdeABC in *Acinetobacter baumannii* is regulated by the AdeRS two-component system. Antimicrob. Agents Chemother. 48, 3298–3304. 10.1128/AAC.48.9.3298-3304.2004 15328088 PMC514774

[B259] Martinez-Guitian M. Vazquez-Ucha J. C. Odingo J. Parish T. Poza M. Waite R. D.. (2016). Synergy between colistin and the signal peptidase inhibitor MD3 is dependent on the mechanism of colistin resistance in *Acinetobacter baumannii*. Antimicrob. Agents Chemother. 60, 4375–4379. 10.1128/AAC.00510-16 27139471 PMC4914650

[B260] Martinez T. Martinez I. Vazquez G. J. Aquino E. E. Robledo I. E. (2016). Genetic environment of the KPC gene in *Acinetobacter baumannii* ST2 clone from Puerto Rico and genomic insights into its drug resistance. J. Med. Microbiol. 65, 784–792. 10.1099/jmm.0.000289 27259867 PMC5756493

[B261] McConnell M. J. Actis L. Pachon J. (2013). *Acinetobacter baumannii*: human infections, factors contributing to pathogenesis and animal models. FEMS Microbiol. Rev. 37, 130–155. 10.1111/j.1574-6976.2012.00344.x 22568581

[B262] McConnell M. J. Rumbo C. Bou G. Pachon J. (2011). Outer membrane vesicles as an acellular vaccine against *Acinetobacter baumannii*. Vaccine 29, 5705–5710. 10.1016/j.vaccine.2011.06.001 21679737

[B263] McQueary C. N. Kirkup B. C. Si Y. Barlow M. Actis L. A. Craft D. W.. (2012). Extracellular stress and lipopolysaccharide modulate *Acinetobacter baumannii* surface-associated motility. J. Microbiol. 50, 434–443. 10.1007/s12275-012-1555-1 22752907

[B264] Megeed A. A. Hayssam M. A. Salem M. Z. El-Shikh M. S. Talea I. A. Alogaibi Y. A. (2016). Investigation of the virulence factors and molecular characterization of the clonal relations of multidrug-resistant *Acinetobacter baumannii* isolates. J. AOAC Int. [Epub ahead of print]. 10.5740/jaoacint.16-0139 27765082

[B265] Mendes R. E. Bell J. M. Turnidge J. D. Castanheira M. Jones R. N. (2009). Emergence and widespread dissemination of OXA-23, -24/40 and -58 carbapenemases among *Acinetobacter* spp. in Asia-Pacific nations: report from the SENTRY Surveillance Program. J. Antimicrob. Chemother. 63, 55–59. 10.1093/jac/dkn434 18957398

[B266] Mendes R. E. Farrell D. J. Sader H. S. Jones R. N. (2010). Comprehensive assessment of tigecycline activity tested against a worldwide collection of *Acinetobacter* spp. (2005–2009). Diagn. Microbiol. Infect. Dis. 68, 307–311. 10.1016/j.diagmicrobio.2010.07.003 20955916

[B267] Menegucci T. C. Albiero J. Migliorini L. B. Alves J. L. Viana G. F. Mazucheli J.. (2016). Strategies for the treatment of polymyxin B-resistant *Acinetobacter baumannii* infections. Int. J. Antimicrob. Agents 47, 380–385. 10.1016/j.ijantimicag.2016.02.007 27068675

[B268] Merino M. Acosta J. Poza M. Sanz F. Beceiro A. Chaves F.. (2010). OXA-24 carbapenemase gene flanked by XerC/XerD-like recombination sites in different plasmids from different *Acinetobacter* species isolated during a nosocomial outbreak. Antimicrob. Agents Chemother. 54, 2724–2727. 10.1128/AAC.01674-09 20385865 PMC2876395

[B269] Moffatt J. H. Harper M. Harrison P. Hale J. D. Vinogradov E. Seemann T.. (2010). Colistin resistance in *Acinetobacter baumannii* is mediated by complete loss of lipopolysaccharide production. Antimicrob. Agents Chemother. 54, 4971–4977. 10.1128/AAC.00834-10 20855724 PMC2981238

[B270] Moland E. S. Craft D. W. Hong S. G. Kim S. Y. Hachmeister L. Sayed S. D.. (2008). *In vitro* activity of tigecycline against multidrug-resistant *Acinetobacter baumannii* and selection of tigecycline-amikacin synergy. Antimicrob. Agents Chemother. 52, 2940–2942. 10.1128/AAC.01581-07 18519722 PMC2493109

[B271] Montero A. Ariza J. Corbella X. Domenech A. Cabellos C. Ayats J.. (2004). Antibiotic combinations for serious infections caused by carbapenem-resistant *Acinetobacter baumannii* in a mouse pneumonia model. J. Antimicrob. Chemother. 54, 1085–1091. 10.1093/jac/dkh485 15546972

[B272] Moon D. C. Choi C. H. Lee J. H. Choi C. W. Kim H. Y. Park J. S.. (2012). *Acinetobacter baumannii* outer membrane protein A modulates the biogenesis of outer membrane vesicles. J. Microbiol. 50, 155–160. 10.1007/s12275-012-1589-4 22367951

[B273] Mosqueda N. Espinal P. Cosgaya C. Viota S. Plasensia V. Alvarez-Lerma F.. (2013). Globally expanding carbapenemase finally appears in Spain: nosocomial outbreak of *Acinetobacter baumannii* producing plasmid-encoded OXA-23 in Barcelona, Spain. Antimicrob. Agents Chemother. 57, 5155–5157. 10.1128/AAC.01486-13 23877694 PMC3811394

[B274] Motaouakkil S. Charra B. Hachimi A. Nejmi H. Benslama A. Elmdaghri N.. (2006). Colistin and rifampicin in the treatment of nosocomial infections from multiresistant *Acinetobacter baumannii*. J. Infect. 53, 274–278. 10.1016/j.jinf.2005.11.019 16442632

[B275] Moubareck C. Bremont S. Conroy M. C. Courvalin P. Lambert T. (2009). GES-11, a novel integron-associated GES variant in *Acinetobacter baumannii*. Antimicrob. Agents Chemother. 53, 3579–3581. 10.1128/AAC.00072-09 19451292 PMC2715621

[B276] Mougous J. D. Cuff M. E. Raunser S. Shen A. Zhou M. Gifford C. A.. (2006). A virulence locus of *Pseudomonas aeruginosa* encodes a protein secretion apparatus. Science 312, 1526–1530. 10.1126/science.1128393 16763151 PMC2800167

[B277] Moya B. Juan C. Alberti S. Perez J. L. Oliver A. (2008). Benefit of having multiple *ampD* genes for acquiring β-lactam resistance without losing fitness and virulence in *Pseudomonas aeruginosa*. Antimicrob. Agents Chemother. 52, 3694–3700. 10.1128/AAC.00172-08 18644952 PMC2565882

[B278] Mugnier P. D. Poirel L. Naas T. Nordmann P. (2010). Worldwide dissemination of the *bla*_OXA-23_ carbapenemase gene of *Acinetobacter baumannii*. Emerg. Infect. Dis. 16, 35–40. 10.3201/eid1601.090852 20031040 PMC2874364

[B279] Mussi M. A. Limansky A. S. Viale A. M. (2005). Acquisition of resistance to carbapenems in multidrug-resistant clinical strains of *Acinetobacter baumannii*: natural insertional inactivation of a gene encoding a member of a novel family of b-barrel outer membrane proteins. Antimicrob. Agents Chemother. 49, 1432–1440. 10.1128/AAC.49.4.1432-1440.2005 15793123 PMC1068641

[B280] Mussi M. A. Relling V. M. Limansky A. S. Viale A. M. (2007). CarO, an *Acinetobacter baumannii* outer membrane protein involved in carbapenem resistance, is essential for L-ornithine uptake. FEBS Lett. 581, 5573–5578. 10.1016/j.febslet.2007.10.063 17997983

[B281] Muthusamy D. Sudhishnaa S. Boppe A. (2016). *In vitro* activities of polymyxins and rifampicin against carbapenem resistant *Acinetobacter baumannii* at a tertiary care hospital from south India. J. Clin. Diagn. Res. 10, DC15–DC18. 10.7860/JCDR/2016/19968.8535 PMC5071932 27790432

[B282] Mutlu Yilmaz E. Sunbul M. Aksoy A. Yilmaz H. Guney A. K. Guvenc T. (2012). Efficacy of tigecycline/colistin combination in a pneumonia model caused by extensively drug-resistant *Acinetobacter baumannii*. Int. J. Antimicrob. Agents 40, 332–336. 10.1016/j.ijantimicag.2012.06.003 22831842

[B283] Naas T. Coignard B. Carbonne A. Blanckaert K. Bajolet O. Bernet C.. (2006). VEB-1 Extended-spectrum β-lactamase-producing *Acinetobacter baumannii*, France. Emerg. Infect. Dis. 12, 1214–1222. 10.3201/eid1208.051547 16965700 PMC3291215

[B284] Naas T. Levy M. Hirschauer C. Marchandin H. Nordmann P. (2005). Outbreak of carbapenem-resistant *Acinetobacter baumannii* producing the carbapenemase OXA-23 in a tertiary care hospital of Papeete, French Polynesia. J. Clin. Microbiol. 43, 4826–4829. 10.1128/JCM.43.9.4826-4829.2005 16145150 PMC1234128

[B285] Naas T. Namdari F. Reglier-Poupet H. Poyart C. Nordmann P. (2007). Panresistant extended-spectrum β-lactamase SHV-5-producing *Acinetobacter baumannii* from New York City. J. Antimicrob. Chemother. 60, 1174–1176. 10.1093/jac/dkm366 17881631

[B286] Nagano N. Nagano Y. Cordevant C. Shibata N. Arakawa Y. (2004). Nosocomial transmission of CTX-M-2 β-lactamase-producing *Acinetobacter baumannii* in a neurosurgery ward. J. Clin. Microbiol. 42, 3978–3984. 10.1128/JCM.42.9.3978-3984.2004 15364979 PMC516360

[B287] Nairn B. L. Lonergan Z. R. Wang J. Braymer J. J. Zhang Y. Calcutt M. W.. (2016). The Response of *Acinetobacter baumannii* to Zinc Starvation. Cell Host Microbe. 19, 826–836. 10.1016/j.chom.2016.05.007 27281572 PMC4901392

[B288] Navia M. M. Ruiz J. Vila J. (2002). Characterization of an integron carrying a new class D β-lactamase (OXA-37) in *Acinetobacter baumannii*. Microb. Drug Resist. 8, 261–265. 10.1089/10766290260469516 12523622

[B289] Navon-Venezia S. Leavitt A. Carmeli Y. (2007). High tigecycline resistance in multidrug-resistant *Acinetobacter baumannii*. J. Antimicrob. Chemother. 59, 772–774. 10.1093/jac/dkm018 17353223

[B290] Nemec A. Dolzani L. Brisse S. van den Broek P. Dijkshoorn L. (2004). Diversity of aminoglycoside-resistance genes and their association with class 1 integrons among strains of pan-European *Acinetobacter baumannii* clones. J. Med. Microbiol. 53, 1233–1240. 10.1099/jmm.0.45716-0 15585503

[B291] Nepka M. Perivolioti E. Kraniotaki E. Politi L. Tsakris A. Pournaras S. (2016). *In vitro* bactericidal activity of trimethoprim-sulfamethoxazole alone and in combination with colistin, against carbapenem-resistant *Acinetobacter baumannii* clinical isolates. Antimicrob. Agents Chemother. 10.1128/AAC.01082-16 27550356 PMC5075104

[B292] Ni W. Li Y. Guan J. Zhao J. Cui J. Wang R.. (2016). Effects of efflux pump inhibitors on colistin resistance in multidrug-resistant Gram-negative gacteria. Antimicrob. Agents Chemother. 60, 3215–3218. 10.1128/AAC.00248-16 26953203 PMC4862505

[B293] Norton M. D. Spilkia A. J. Godoy V. G. (2013). Antibiotic resistance acquired through a DNA damage-inducible response in *Acinetobacter baumannii*. J. Bacteriol. 195, 1335–1345. 10.1128/JB.02176-12 23316046 PMC3591989

[B294] Nowak-Zaleska A. Wieczor M. Czub J. Nierzwicki L. Kotlowski R. Mikucka A.. (2016). Correlation between the number of Pro-Ala repeats in the EmrA homologue of *Acinetobacter baumannii* and resistance to netilmicin, tobramycin, imipenem and ceftazidime. J. Glob. Antimicrob. Resist. 7, 145–149. 10.1016/j.jgar.2016.09.004 27835840

[B295] Ozbek B. Senturk A. (2010). Postantibiotic effects of tigecycline, colistin sulfate, and levofloxacin alone or tigecycline-colistin sulfate and tigecycline-levofloxacin combinations against *Acinetobacter baumannii*. Chemotherapy 56, 466–471. 10.1159/000321015 21088399

[B296] Pachon-Ibanez M. E. Docobo-Perez F. Lopez-Rojas R. Dominguez-Herrera J. Jimenez-Mejias M. E. Garcia-Curiel A.. (2010). Efficacy of rifampin and its combinations with imipenem, sulbactam, and colistin in experimental models of infection caused by imipenem-resistant *Acinetobacter baumannii*. Antimicrob. Agents Chemother. 54, 1165–1172. 10.1128/AAC.00367-09 20047914 PMC2825983

[B297] Pachon-Ibanez M. E. Jimenez-Mejias M. E. Pichardo C. Llanos A. C. Pachon J. (2004). Activity of tigecycline (GAR-936) against *Acinetobacter baumannii* strains, including those resistant to imipenem. Antimicrob. Agents Chemother. 48, 4479–4481. 10.1128/AAC.48.11.4479-4481.2004 15504889 PMC525443

[B298] Pailhories H. Kempf M. Belmonte O. Joly-Guillou M. L. Eveillard M. (2016). First case of OXA-24-producing *Acinetobacter baumannii* in cattle from Reunion Island, France. Int. J. Antimicrob. Agents 48, 763–764. 10.1016/j.ijantimicag.2016.09.005 27771188

[B299] Pankuch G. A. Lin G. Seifert H. Appelbaum P. C. (2008). Activity of meropenem with and without ciprofloxacin and colistin against *Pseudomonas aeruginosa* and *Acinetobacter baumannii*. Antimicrob. Agents Chemother. 52, 333–336. 10.1128/AAC.00689-07 17967915 PMC2223893

[B300] Pannek S. Higgins P. G. Steinke P. Jonas D. Akova M. Bohnert J. A.. (2006). Multidrug efflux inhibition in *Acinetobacter baumannii*: comparison between 1-(1-naphthylmethyl)-piperazine and phenyl-arginine-b-naphthylamide. J. Antimicrob. Chemother. 57, 970–974. 10.1093/jac/dkl081 16531429

[B301] Pantopoulou A. Giamarellos-Bourboulis E. J. Raftogannis M. Tsaganos T. Dontas I. Koutoukas P.. (2007). Colistin offers prolonged survival in experimental infection by multidrug-resistant *Acinetobacter baumannii*: the significance of co-administration of rifampicin. Int. J. Antimicrob. Agents 29, 51–55. 10.1016/j.ijantimicag.2006.09.009 17189095

[B302] Papa A. Koulourida V. Souliou E. (2009). Molecular epidemiology of carbapenem-resistant *Acinetobacter baumannii* in a newly established Greek hospital. Microb. Drug Resist. 15, 257–260. 10.1089/mdr.2009.0060 19857131

[B303] Park Y. K. Ko K. S. (2015). Effect of carbonyl cyanide 3-chlorophenylhydrazone (CCCP) on killing *Acinetobacter baumannii* by colistin. J. Microbiol. 53, 53–59. 10.1007/s12275-015-4498-5 25557480

[B304] Parra Millan R. Jimenez Mejias M. E. Sanchez Encinales V. Ayerbe Algaba R. Gutierrez Valencia A. Pachon Ibanez M. E.. (2016). Efficacy of lysophosphatidylcholine in combination with antimicrobial agents against *Acinetobacter baumannii* in experimental murine peritoneal sepsis and pneumonia models. Antimicrob. Agents Chemother. 60, 4464–4470. 10.1128/AAC.02708-15 27161639 PMC4958192

[B305] Pasteran F. Rapoport M. Petroni A. Faccone D. Corso A. Galas M.. (2006). Emergence of PER-2 and VEB-1a in *Acinetobacter baumannii* strains in the Americas. Antimicrob. Agents Chemother. 50, 3222–3224. 10.1128/AAC.00284-06 16940137 PMC1563550

[B306] Peck K. R. Kim M. J. Choi J. Y. Kim H. S. Kang C. I. Cho Y. K.. (2012). *In vitro* time-kill studies of antimicrobial agents against blood isolates of imipenem-resistant *Acinetobacter baumannii*, including colistin- or tigecycline-resistant isolates. J. Med. Microbiol. 61, 353–360. 10.1099/jmm.0.036939-0 22016557

[B307] Peleg A. Y. Adams J. Paterson D. L. (2007). Tigecycline efflux as a mechanism for nonsusceptibility in *Acinetobacter baumannii*. Antimicrob. Agents Chemother. 51, 2065–2069. 10.1128/AAC.01198-06 17420217 PMC1891386

[B308] Peleg A. Y. Seifert H. Paterson D. L. (2008). *Acinetobacter baumannii*: emergence of a successful pathogen. Clin. Microbiol. Rev. 21, 538–582. 10.1128/CMR.00058-07 18625687 PMC2493088

[B309] Pelletier M. R. Casella L. G. Jones J. W. Adams M. D. Zurawski D. V. Hazlett K. R.. (2013). Unique structural modifications are present in the lipopolysaccharide from colistin-resistant strains of *Acinetobacter baumannii*. Antimicrob. Agents Chemother. 57, 4831–4840. 10.1128/AAC.00865-13 23877686 PMC3811424

[B310] Penwell W. F. Arivett B. A. Actis L. A. (2012). The *Acinetobacter baumannii entA* gene located outside the acinetobactin cluster is critical for siderophore production, iron acquisition and virulence. PLoS ONE 7:e36493. 10.1371/journal.pone.0036493 22570720 PMC3343012

[B311] Perez A. Merino M. Rumbo-Feal S. Alvarez-Fraga L. Vallejo J. A. Beceiro A.. (2016). The FhaB/FhaC two-partner secretion system is involved in adhesion of *Acinetobacter baumannii* AbH12O-A2 strain. Virulence. [Epub ahead of print]. 10.1080/21505594.2016.1262313 27858524 PMC5626241

[B312] Perez F. Hujer A. M. Hujer K. M. Decker B. K. Rather P. N. Bonomo R. A. (2007). Global challenge of multidrug-resistant *Acinetobacter baumannii*. Antimicrob. Agents Chemother. 51, 3471–3484. 10.1128/AAC.01464-06 17646423 PMC2043292

[B313] Petrosillo N. Chinello P. Proietti M. F. Cecchini L. Masala M. Franchi C.. (2005). Combined colistin and rifampicin therapy for carbapenem-resistant *Acinetobacter baumannii* infections: clinical outcome and adverse events. Clin. Microbiol. Infect. 11, 682–683. 10.1111/j.1469-0691.2005.01198.x 16008625

[B314] Pfeifer Y. Wilharm G. Zander E. Wichelhaus T. A. Gottig S. Hunfeld K. P.. (2011). Molecular characterization of *bla*_NDM−1_ in an *Acinetobacter baumannii* strain isolated in Germany in 2007. J. Antimicrob. Chemother. 66, 1998–2001. 10.1093/jac/dkr256 21693460

[B315] Phee L. M. Betts J. W. Bharathan B. Wareham D. W. (2015). Colistin and fusidic acid, a novel potent synergistic combination for treatment of multidrug-resistant *Acinetobacter baumannii* infections. Antimicrob. Agents Chemother. 59, 4544–4550. 10.1128/AAC.00753-15 25987639 PMC4505221

[B316] Pichardo C. Pachon-Ibanez M. E. Docobo-Perez F. Lopez-Rojas R. Jimenez-Mejias M. E. Garcia-Curiel A.. (2010). Efficacy of tigecycline vs. imipenem in the treatment of experimental Acinetobacter baumannii murine pneumonia. Eur. J. Clin. Microbiol. Infect. Dis. 29, 527–531. 10.1007/s10096-010-0890-6 20182760

[B317] Pires J. Siriwardena T. N. Stach M. Tinguely R. Kasraian S. Luzzaro F.. (2015). *In vitro* activity of the novel antimicrobial peptide dendrimer G3KL against multidrug-resistant *Acinetobacter baumannii* and *Pseudomonas aeruginosa*. Antimicrob. Agents Chemother. 59, 7915–7918. 10.1128/AAC.01853-15 26459893 PMC4649178

[B318] Ploy M. C. Giamarellou H. Bourlioux P. Courvalin P. Lambert T. (1994). Detection of *aac(6*′*)*-I genes in amikacin-resistant *Acinetobacter* spp. by PCR. Antimicrob. Agents Chemother. 38, 2925–2928. 7695286 10.1128/aac.38.12.2925 PMC188310

[B319] Poirel L. Cabanne L. Vahaboglu H. Nordmann P. (2005a). Genetic environment and expression of the extended-spectrum β-lactamase *bla*_PER-1_ gene in gram-negative bacteria. Antimicrob. Agents Chemother. 49, 1708–1713. 10.1128/AAC.49.5.1708-1713.2005 15855485 PMC1087670

[B320] Poirel L. Corvec S. Rapoport M. Mugnier P. Petroni A. Pasteran F.. (2007). Identification of the novel narrow-spectrum β-lactamase SCO-1 in *Acinetobacter* spp. from Argentina. Antimicrob. Agents Chemother. 51, 2179–2184. 10.1128/AAC.01600-06 17420213 PMC1891420

[B321] Poirel L. Mansour W. Bouallegue O. Nordmann P. (2008). Carbapenem-resistant *Acinetobacter baumannii* isolates from Tunisia producing the OXA-58-like carbapenem-hydrolyzing oxacillinase OXA-97. Antimicrob. Agents Chemother. 52, 1613–1617. 10.1128/AAC.00978-07 18299404 PMC2346634

[B322] Poirel L. Marque S. Heritier C. Segonds C. Chabanon G. Nordmann P. (2005b). OXA-58, a novel class D β-lactamase involved in resistance to carbapenems in *Acinetobacter baumannii*. Antimicrob. Agents Chemother. 49, 202–208. 10.1128/AAC.49.1.202-208.2005 15616297 PMC538857

[B323] Poirel L. Mugnier P. D. Toleman M. A. Walsh T. R. Rapoport M. J. Petroni A.. (2009). IS*CR2*, another vehicle for *bla*_VEB_ gene acquisition. Antimicrob. Agents Chemother. 53, 4940–4943. 10.1128/AAC.00414-09 19704129 PMC2772328

[B324] Pongpech P. Amornnopparattanakul S. Panapakdee S. Fungwithaya S. Nannha P. Dhiraputra C.. (2010). Antibacterial activity of carbapenem-based combinations againts multidrug-resistant *Acinetobacter baumannii*. J. Med. Assoc. Thai. 93, 161–171. 20301995

[B325] Potron A. Munoz-Price L. S. Nordmann P. Cleary T. Poirel L. (2011). Genetic features of CTX-M-15-producing *Acinetobacter baumannii* from Haiti. Antimicrob. Agents Chemother. 55, 5946–5948. 10.1128/AAC.05124-11 21930877 PMC3232807

[B326] Potron A. Poirel L. Croize J. Chanteperdrix V. Nordmann P. (2009). Genetic and biochemical characterization of the first extended-spectrum CARB-type b-lactamase, RTG-4, from *Acinetobacter baumannii*. Antimicrob. Agents Chemother. 53, 3010–3016. 10.1128/AAC.01164-08 19380596 PMC2704689

[B327] Pournaras S. Markogiannakis A. Ikonomidis A. Kondyli L. Bethimouti K. Maniatis A. N.. (2006). Outbreak of multiple clones of imipenem-resistant *Acinetobacter baumannii* isolates expressing OXA-58 carbapenemase in an intensive care unit. J. Antimicrob. Chemother. 57, 557–561. 10.1093/jac/dkl004 16431857

[B328] Principe L. Capone A. Mazzarelli A. D'Arezzo S. Bordi E. Di Caro A.. (2013). *In vitro* activity of doripenem in combination with various antimicrobials against multidrug-resistant *Acinetobacter baumannii*: possible options for the treatment of complicated infection. Microb. Drug Resist. 19, 407–414. 10.1089/mdr.2012.0250 23659601

[B329] Principe L. D'Arezzo S. Capone A. Petrosillo N. Visca P. (2009). *In vitro* activity of tigecycline in combination with various antimicrobials against multidrug resistant *Acinetobacter baumannii*. Ann. Clin. Microbiol. Antimicrob. 8:18. 10.1186/1476-0711-8-18 19460166 PMC2693502

[B330] Principe L. Piazza A. Giani T. Bracco S. Caltagirone M. S. Arena F.. (2014). Epidemic diffusion of OXA-23-producing *Acinetobacter baumannii* isolates in Italy: results of the first cross-sectional countrywide survey. J. Clin. Microbiol. 52, 3004–3010. 10.1128/JCM.00291-14 24920776 PMC4136168

[B331] Pukatzki S. Ma A. T. Sturtevant D. Krastins B. Sarracino D. Nelson W. C.. (2006). Identification of a conserved bacterial protein secretion system in *Vibrio cholerae* using the Dictyostelium host model system. Proc. Natl. Acad. Sci. U.S.A. 103, 1528–1533. 10.1073/pnas.0510322103 16432199 PMC1345711

[B332] Qi C. Malczynski M. Parker M. Scheetz M. H. (2008). Characterization of genetic diversity of carbapenem-resistant *Acinetobacter baumannii* clinical strains collected from 2004 to 2007. J. Clin. Microbiol. 46, 1106–1109. 10.1128/JCM.01877-07 18216212 PMC2268351

[B333] Quale J. Bratu S. Landman D. Heddurshetti R. (2003). Molecular epidemiology and mechanisms of carbapenem resistance in *Acinetobacter baumannii* endemic in New York City. Clin. Infect. Dis. 37, 214–220. 10.1086/375821 12856214

[B334] Quinteira S. Grosso F. Ramos H. Peixe L. (2007). Molecular epidemiology of imipenem-resistant *Acinetobacter haemolyticus* and *Acinetobacter baumannii* isolates carrying plasmid-mediated OXA-40 from a Portuguese hospital. Antimicrob. Agents Chemother. 51, 3465–3466. 10.1128/AAC.00267-07 17606684 PMC2043188

[B335] Rafailidis P. I. Ioannidou E. N. Falagas M. E. (2007). Ampicillin/sulbactam: current status in severe bacterial infections. Drugs 67, 1829–1849. 10.2165/00003495-200767130-00003 17722953

[B336] Rajamohan G. Srinivasan V. B. Gebreyes W. A. (2010). Molecular and functional characterization of a novel efflux pump, AmvA, mediating antimicrobial and disinfectant resistance in *Acinetobacter baumannii*. J. Antimicrob. Chemother. 65, 1919–1925. 10.1093/jac/dkq195 20573661

[B337] Rakin A. Schneider L. Podladchikova O. (2012). Hunger for iron: the alternative siderophore iron scavenging systems in highly virulent *Yersinia*. Front. Cell. Infect. Microbiol. 2:151. 10.3389/fcimb.2012.00151 23226687 PMC3510459

[B338] Ramirez M. S. Don M. Merkier A. K. Bistue A. J. Zorreguieta A. Centron D.. (2010a). Naturally competent *Acinetobacter baumannii* clinical isolate as a convenient model for genetic studies. J. Clin. Microbiol. 48, 1488–1490. 10.1128/JCM.01264-09 20181905 PMC2849597

[B339] Ramirez M. S. Pineiro S. Argentinian Integron Study Group Centron D. (2010b). Novel insights about class 2 integrons from experimental and genomic epidemiology. Antimicrob. Agents Chemother. 54, 699–706. 10.1128/AAC.01392-08 19917745 PMC2812161

[B340] Rao G. G. Ly N. S. Bulitta J. B. Soon R. L. San Roman M. D. Holden P. N.. (2016a). Polymyxin B in combination with doripenem against heteroresistant *Acinetobacter baumannii:* pharmacodynamics of new dosing strategies. J. Antimicrob. Chemother. 71, 3148–3156. 10.1093/jac/dkw293 27494922 PMC5079300

[B341] Rao G. G. Ly N. S. Diep J. Forrest A. Bulitta J. B. Holden P. N.. (2016b). Combinatorial pharmacodynamics of polymyxin B and tigecycline against heteroresistant *Acinetobacter baumannii*. Int. J. Antimicrob. Agents 48, 331–336. 10.1016/j.ijantimicag.2016.06.006 27449542 PMC5256686

[B342] Ravasi P. Limansky A. S. Rodriguez R. E. Viale A. M. Mussi M. A. (2011). IS*Aba825*, a functional insertion sequence modulating genomic plasticity and *bla*_OXA-58_ expression in *Acinetobacter baumannii*. Antimicrob. Agents Chemother. 55, 917–920. 10.1128/AAC.00491-10 21098239 PMC3028783

[B343] Reid G. Food Agricultural Organization of the United Nation the WHO (2005). The importance of guidelines in the development and application of probiotics. Curr. Pharm. Des. 11, 11–16. 10.2174/1381612053382395 15638748

[B344] Repizo G. D. Gagne S. Foucault-Grunenwald M. L. Borges V. Charpentier X. Limansky A. S.. (2015). Differential Role of the T6SS in *Acinetobacter baumannii* Virulence. PLoS ONE 10:e0138265. 10.1371/journal.pone.0138265 26401654 PMC4581634

[B345] Ribera A. Roca I. Ruiz J. Gibert I. Vila J. (2003a). Partial characterization of a transposon containing the *tet(A)* determinant in a clinical isolate of *Acinetobacter baumannii*. J. Antimicrob. Chemother. 52, 477–480. 10.1093/jac/dkg344 12888597

[B346] Ribera A. Ruiz J. Vila J. (2003b). Presence of the Tet M determinant in a clinical isolate of *Acinetobacter baumannii*. Antimicrob. Agents Chemother. 47, 2310–2312. 10.1128/AAC.47.7.2310-2312.2003 12821485 PMC161866

[B347] Riccio M. L. Franceschini N. Boschi L. Caravelli B. Cornaglia G. Fontana R.. (2000). Characterization of the metallo-β-lactamase determinant of *Acinetobacter baumannii* AC-54/97 reveals the existence of *bla*_IMP_ allelic variants carried by gene cassettes of different phylogeny. Antimicrob. Agents Chemother. 44, 1229–1235. 10.1128/AAC.44.5.1229-1235.2000 10770756 PMC89849

[B348] Ritchie D. J. Garavaglia-Wilson A. (2014). A review of intravenous minocycline for treatment of multidrug-resistant *Acinetobacter* infections. Clin. Infect. Dis. 59 (Suppl. 6), S374–S380. 10.1093/cid/ciu613 25371513

[B349] Robledo I. E. Aquino E. E. Sante M. I. Santana J. L. Otero D. M. Leon C. F.. (2010). Detection of KPC in *Acinetobacter* spp. in Puerto Rico. Antimicrob. Agents Chemother. 54, 1354–1357. 10.1128/AAC.00899-09 20038618 PMC2825984

[B350] Roca I. Marti S. Espinal P. Martinez P. Gibert I. Vila J. (2009). CraA, a major facilitator superfamily efflux pump associated with chloramphenicol resistance in *Acinetobacter baumannii*. Antimicrob. Agents Chemother. 53, 4013–4014. 10.1128/AAC.00584-09 19581458 PMC2737869

[B351] Rodriguez-Bano J. Marti S. Soto S. Fernandez-Cuenca F. Cisneros J. M. Pachon J.. (2008). Biofilm formation in *Acinetobacter baumannii:* associated features and clinical implications. Clin. Microbiol. Infect. 14, 276–278. 10.1111/j.1469-0691.2007.01916.x 18190568

[B352] Rodriguez C. H. De Ambrosio A. Bajuk M. Spinozzi M. Nastro M. Bombicino K.. (2010). *In vitro* antimicrobials activity against endemic *Acinetobacter baumannii* multiresistant clones. J. Infect. Dev. Ctries. 4, 164–167. 10.3855/jidc.604 20351457

[B353] Rodriguez C. H. Nastro M. Vay C. Famiglietti A. (2015). *In vitro* activity of minocycline alone or in combination in multidrug-resistant *Acinetobacter baumannii* isolates. J. Med. Microbiol. 64, 1196–1200. 10.1099/jmm.0.000147 26238719

[B354] Rodriguez-Rubio L. Chang W. L. Gutierrez D. Lavigne R. Martinez B. Rodriguez A.. (2016). ‘Artilysation’ of endolysin lambdaSa2lys strongly improves its enzymatic and antibacterial activity against streptococci. Sci. Rep. 6:35382. 10.1038/srep35382 27775093 PMC5075790

[B355] Rosenfeld N. Bouchier C. Courvalin P. Perichon B. (2012). Expression of the resistance-nodulation-cell division pump AdeIJK in *Acinetobacter baumannii* is regulated by AdeN, a TetR-type regulator. Antimicrob. Agents Chemother. 56, 2504–2510. 10.1128/AAC.06422-11 22371895 PMC3346617

[B356] Ruiz F. M. Santillana E. Spinola-Amilibia M. Torreira E. Culebras E. Romero A. (2015). Crystal Structure of Hcp from *Acinetobacter baumannii:* a Component of the Type VI Secretion System. PLoS ONE 10:e0129691. 10.1371/journal.pone.0129691 26079269 PMC4469607

[B357] Ruiz M. Marti S. Fernandez-Cuenca F. Pascual A. Vila J. (2007). High prevalence of carbapenem-hydrolysing oxacillinases in epidemiologically related and unrelated *Acinetobacter baumannii* clinical isolates in Spain. Clin. Microbiol. Infect. 13, 1192–1198. 10.1111/j.1469-0691.2007.01825.x 17850347

[B358] Rumbo C. Fernandez-Moreira E. Merino M. Poza M. Mendez J. A. Soares N. C.. (2011). Horizontal transfer of the OXA-24 carbapenemase gene via outer membrane vesicles: a new mechanism of dissemination of carbapenem resistance genes in *Acinetobacter baumannii*. Antimicrob. Agents Chemother. 55, 3084–3090. 10.1128/AAC.00929-10 21518847 PMC3122458

[B359] Rumbo C. Tomas M. Fernandez Moreira E. Soares N. C. Carvajal M. Santillana E.. (2014). The *Acinetobacter baumannii* Omp33-36 porin is a virulence factor that induces apoptosis and modulates autophagy in human cells. Infect. Immun. 82, 4666–4680. 10.1128/IAI.02034-14 25156738 PMC4249306

[B360] Rumbo C. Vallejo J. A. Cabral M. P. Martinez-Guitian M. Perez A. Beceiro A.. (2016). Assessment of antivirulence activity of several D-amino acids against *Acinetobacter baumannii* and *Pseudomonas aeruginosa*. J. Antimicrob. Chemother. 71, 3473–3481. 10.1093/jac/dkw342 27605598

[B361] Russo T. A. Beanan J. M. Olson R. MacDonald U. Cox A. D. St. Michael F.. (2013). The K1 capsular polysaccharide from *Acinetobacter baumannii* is a potential therapeutic target via passive immunization. Infect. Immun. 81, 915–922. 10.1128/IAI.01184-12 23297385 PMC3584894

[B362] Russo T. A. Luke N. R. Beanan J. M. Olson R. Sauberan S. L. MacDonald U.. (2010). The K1 capsular polysaccharide of *Acinetobacter baumannii* strain 307-0294 is a major virulence factor. Infect. Immun. 78, 3993–4000. 10.1128/IAI.00366-10 20643860 PMC2937447

[B363] Russo T. A. MacDonald U. Beanan J. M. Olson R. MacDonald I. J. Sauberan S. L.. (2009). Penicillin-binding protein 7/8 contributes to the survival of *Acinetobacter baumannii in vitro* and *in vivo*. J. Infect. Dis. 199, 513–521. 10.1086/596317 19143563

[B364] Russo T. A. Manohar A. Beanan J. M. Olson R. MacDonald U. Graham J.. (2016). The response regulator BfmR is a potential drug target for *Acinetobacter baumannii*. mSphere 1:e00082–16. 10.1128/mSphere.00082-16 27303741 PMC4888885

[B365] Ruzin A. Keeney D. Bradford P. A. (2007). AdeABC multidrug efflux pump is associated with decreased susceptibility to tigecycline in *Acinetobacter calcoaceticus*-*Acinetobacter baumannii* complex. J. Antimicrob. Chemother. 59, 1001–1004. 10.1093/jac/dkm058 17363424

[B366] Saha R. Saha N. Donofrio R. S. Bestervelt L. L. (2013). Microbial siderophores: a mini review. J. Basic Microbiol. 53, 303–317. 10.1002/jobm.201100552 22733623

[B367] Sahly H. Navon-Venezia S. Roesler L. Hay A. Carmeli Y. Podschun R.. (2008). Extended-spectrum β-lactamase production is associated with an increase in cell invasion and expression of fimbrial adhesins in *Klebsiella pneumoniae*. Antimicrob. Agents Chemother. 52, 3029–3034. 10.1128/AAC.00010-08 18573929 PMC2533491

[B368] Sakoulas G. Rose W. Berti A. Olson J. Mungia J. Nonejuie P.. (2016). Classical β-lactamase inhibitors potentiate the activity of daptomycin against methicillin-resistant *Staphylococcus aureus* and colistin against *Acinetobacter baumannii*. Antimicrob. Agents Chemother. 61:e01745–16. 10.1128/AAC.01745-16 27872080 PMC5278754

[B369] Sandri A. M. Landersdorfer C. B. Jacob J. Boniatti M. M. Dalarosa M. G. Falci D. R.. (2013). Population pharmacokinetics of intravenous polymyxin B in critically ill patients: implications for selection of dosage regimens. Clin. Infect. Dis. 57, 524–531. 10.1093/cid/cit334 23697744

[B370] Santimaleeworagun W. Wongpoowarak P. Chayakul P. Pattharachayakul S. Tansakul P. Garey K. W. (2011). *In vitro* activity of colistin or sulbactam in combination with fosfomycin or imipenem against clinical isolates of carbapenem-resistant *Acinetobacter baumannii* producing OXA-23 carbapenemases. Southeast Asian J. Trop. Med. Public Health. 42, 890–900. 22299471

[B371] Schroder W. Goerke C. Wolz C. (2013). Opposing effects of aminocoumarins and fluoroquinolones on the SOS response and adaptability in *Staphylococcus aureus*. J. Antimicrob. Chemother. 68, 529–538. 10.1093/jac/dks456 23169893

[B372] Sechi L. A. Karadenizli A. Deriu A. Zanetti S. Kolayli F. Balikci E.. (2004). PER-1 type β-lactamase production in *Acinetobacter baumannii* is related to cell adhesion. Med. Sci. Monit. 10, BR180–BR184. 15173664

[B373] Segal H. Nelson E. C. Elisha B. G. (2004). Genetic environment and transcription of *ampC* in an *Acinetobacter baumannii* clinical isolate. Antimicrob. Agents Chemother. 48, 612–614. 10.1128/AAC.48.2.612-614.2004 14742218 PMC321557

[B374] Sharma A. Sharma R. Bhattacharyya T. Bhando T. Pathania R. (2016). Fosfomycin resistance in *Acinetobacter baumannii* is mediated by efflux through a major facilitator superfamily (MFS) transporter-AbaF. J. Antimicrob. Chemother. 72, 68–74. 10.1093/jac/dkw382 27650185

[B375] Sheng W. H. Wang J. T. Li S. Y. Lin Y. C. Cheng A. Chen Y. C.. (2011). Comparative *in vitro* antimicrobial susceptibilities and synergistic activities of antimicrobial combinations against carbapenem-resistant *Acinetobacter* species: *Acinetobacter baumannii* versus *Acinetobacter* genospecies 3 and 13TU. Diagn. Microbiol. Infect. Dis. 70, 380–386. 10.1016/j.diagmicrobio.2011.03.003 21558048

[B376] Shin B. Park W. (2015). Synergistic effect of oleanolic acid on aminoglycoside antibiotics against *Acinetobacter baumannii*. PLoS ONE 10:e0137751. 10.1371/journal.pone.0137751 26360766 PMC4567131

[B377] Shneider M. M. Buth S. A. Ho B. T. Basler M. Mekalanos J. J. Leiman P. G. (2013). PAAR-repeat proteins sharpen and diversify the type VI secretion system spike. Nature 500, 350–353. 10.1038/nature12453 23925114 PMC3792578

[B378] Siroy A. Molle V. Lemaitre-Guillier C. Vallenet D. Pestel-Caron M. Cozzone A. J.. (2005). Channel formation by CarO, the carbapenem resistance-associated outer membrane protein of *Acinetobacter baumannii*. Antimicrob. Agents Chemother. 49, 4876–4883. 10.1128/AAC.49.12.4876-4883.2005 16304148 PMC1315959

[B379] Skalweit M. J. Li M. (2016). Bulgecin A as a β-lactam enhancer for carbapenem-resistant *Pseudomonas aeruginosa* and carbapenem-resistant *Acinetobacter baumannii* clinical isolates containing various resistance mechanisms. Drug Des. Devel. Ther. 10, 3013–3020. 10.2147/DDDT.S110193 27703329 PMC5036594

[B380] Smani Y. Dominguez-Herrera J. Pachon J. (2013). Association of the outer membrane protein Omp33 with fitness and virulence of *Acinetobacter baumannii*. J. Infect. Dis. 208, 1561–1570. 10.1093/infdis/jit386 23908480

[B381] Smani Y. Fabrega A. Roca I. Sanchez-Encinales V. Vila J. Pachon J. (2014). Role of OmpA in the multidrug resistance phenotype of *Acinetobacter baumannii*. Antimicrob. Agents Chemother. 58, 1806–1808. 10.1128/AAC.02101-13 24379205 PMC3957889

[B382] Smani Y. McConnell M. J. Pachon J. (2012). Role of fibronectin in the adhesion of *Acinetobacter baumannii* to host cells. PLoS ONE 7:e33073. 10.1371/journal.pone.0033073 22514602 PMC3326023

[B383] Smith S. G. Mahon V. Lambert M. A. Fagan R. P. (2007). A molecular Swiss army knife: OmpA structure, function and expression. FEMS Microbiol. Lett. 273, 1–11. 10.1111/j.1574-6968.2007.00778.x 17559395

[B384] Smolyakov R. Borer A. Riesenberg K. Schlaeffer F. Alkan M. Porath A.. (2003). Nosocomial multi-drug resistant *Acinetobacter baumannii* bloodstream infection: risk factors and outcome with ampicillin-sulbactam treatment. J. Hosp. Infect. 54, 32–38. 10.1016/S0195-6701(03)00046-X 12767844

[B385] Songer J. G. (1997). Bacterial phospholipases and their role in virulence. Trends Microbiol. 5, 156–161. 10.1016/S0966-842X(97)01005-6 9141190

[B386] Song J. Y. Lee J. Heo J. Y. Noh J. Y. Kim W. J. Cheong H. J.. (2008). Colistin and rifampicin combination in the treatment of ventilator-associated pneumonia caused by carbapenem-resistant *Acinetobacter baumannii*. Int. J. Antimicrob. Agents 32, 281–284. 10.1016/j.ijantimicag.2008.04.013 18650070

[B387] Srinivasan V. B. Rajamohan G. Gebreyes W. A. (2009). Role of AbeS, a novel efflux pump of the SMR family of transporters, in resistance to antimicrobial agents in *Acinetobacter baumannii*. Antimicrob. Agents Chemother. 53, 5312–5316. 10.1128/AAC.00748-09 19770280 PMC2786332

[B388] Srinivasan V. B. Venkataramaiah M. Mondal A. Rajamohan G. (2015). Functional characterization of AbeD, an RND-type membrane transporter in antimicrobial resistance in *Acinetobacter baumannii*. PLoS ONE 10:e0141314. 10.1371/journal.pone.0141314 26496475 PMC4619830

[B389] Stahl J. Bergmann H. Gottig S. Ebersberger I. Averhoff B. (2015). *Acinetobacter baumannii* virulence is mediated by the concerted action of three phospholipases D. PLoS ONE 10:e0138360. 10.1371/journal.pone.0138360 26379240 PMC4574555

[B390] Stoeva T. Higgins P. G. Bojkova K. Seifert H. (2008). Clonal spread of carbapenem-resistant OXA-23-positive *Acinetobacter baumannii* in a Bulgarian university hospital. Clin. Microbiol. Infect. 14, 723–727. 10.1111/j.1469-0691.2008.02018.x 18558947

[B391] Su C. H. Wang J. T. Hsiung C. A. Chien L. J. Chi C. L. Yu H. T.. (2012). Increase of carbapenem-resistant *Acinetobacter baumannii* infection in acute care hospitals in Taiwan: association with hospital antimicrobial usage. PLoS ONE 7:e37788. 10.1371/journal.pone.0037788 22629456 PMC3357347

[B392] Sugawara E. Nikaido H. (2012). OmpA is the principal nonspecific slow porin of *Acinetobacter baumannii*. J. Bacteriol. 194, 4089–4096. 10.1128/JB.00435-12 22636785 PMC3416538

[B393] Sun J. R. Jeng W. Y. Perng C. L. Yang Y. S. Soo P. C. Chiang Y. S.. (2016). Single amino acid substitution Gly186Val in AdeS restores tigecycline susceptibility of *Acinetobacter baumannii*. J. Antimicrob. Chemother. 71, 1488–1492. 10.1093/jac/dkw002 26850720

[B394] Sun J. R. Perng C. L. Chan M. C. Morita Y. Lin J. C. Su C. M.. (2012). A truncated AdeS kinase protein generated by IS*Aba1* insertion correlates with tigecycline resistance in *Acinetobacter baumannii*. PLoS ONE 7:e49534. 10.1371/journal.pone.0049534 23166700 PMC3498117

[B395] Su X. Z. Chen J. Mizushima T. Kuroda T. Tsuchiya T. (2005). AbeM, an H^+^-coupled *Acinetobacter baumannii* multidrug efflux pump belonging to the MATE family of transporters. Antimicrob. Agents Chemother. 49, 4362–4364. 10.1128/AAC.49.10.4362-4364.2005 16189122 PMC1251516

[B396] Tada T. Miyoshi-Akiyama T. Shimada K. Shimojima M. Kirikae T. (2014). Dissemination of 16S rRNA methylase ArmA-producing *Acinetobacter baumannii* and emergence of OXA-72 carbapenemase coproducers in Japan. Antimicrob. Agents Chemother. 58, 2916–2920. 10.1128/AAC.01212-13 24550340 PMC3993269

[B397] Taitt C. R. Leski T. A. Stockelman M. G. Craft D. W. Zurawski D. V. Kirkup B. C.. (2014). Antimicrobial resistance determinants in *Acinetobacter baumannii* isolates taken from military treatment facilities. Antimicrob. Agents Chemother. 58, 767–781. 10.1128/AAC.01897-13 24247131 PMC3910874

[B398] Thandar M. Lood R. Winer B. Y. Deutsch D. R. Euler C. W. Fischetti V. A. (2016). Novel engineered peptides of a phage lysin as effective antimicrobials against multidrug-resistant *Acinetobacter baumannii*. Antimicrob. Agents Chemother. 60, 2671–2679. 10.1128/AAC.02972-15 26856847 PMC4862495

[B399] Thummeepak R. Kitti T. Kunthalert D. Sitthisak S. (2016). Enhanced antibacterial activity of *Acinetobacter baumannii* bacteriophage OABP-01 endolysin (LysABP-01) in combination with colistin. Front. Microbiol. 7:1402. 10.3389/fmicb.2016.01402 27656173 PMC5013039

[B400] Timurkaynak F. Can F. Azap O. K. Demirbilek M. Arslan H. Karaman S. O. (2006). *In vitro* activities of non-traditional antimicrobials alone or in combination against multidrug-resistant strains of *Pseudomonas aeruginosa* and *Acinetobacter baumannii* isolated from intensive care units. Int. J. Antimicrob. Agents 27, 224–228. 10.1016/j.ijantimicag.2005.10.012 16464562

[B401] Tipton K. A. Rather P. N. (2016). An ompR/envZ two-component system ortholog regulates phase variation, osmotic tolerance, motility, and virulence in *Acinetobacter baumannii* strain AB5075. J. Bacteriol. [Epub ahead of print]. 10.1128/JB.00705-16 27872182 PMC5237114

[B402] Tognim M. C. Gales A. C. Penteado A. P. Silbert S. Sader H. S. (2006). Dissemination of IMP-1 metallo- beta -lactamase-producing *Acinetobacter* species in a Brazilian teaching hospital. Infect. Control Hosp. Epidemiol. 27, 742–747. 10.1086/504356 16807851

[B403] Tomaras A. P. Dorsey C. W. Edelmann R. E. Actis L. A. (2003). Attachment to and biofilm formation on abiotic surfaces by *Acinetobacter baumannii:* involvement of a novel chaperone-usher pili assembly system. Microbiology 149, 3473–3484. 10.1099/mic.0.26541-0 14663080

[B404] Tomaras A. P. Flagler M. J. Dorsey C. W. Gaddy J. A. Actis L. A. (2008). Characterization of a two-component regulatory system from *Acinetobacter baumannii* that controls biofilm formation and cellular morphology. Microbiology 154, 3398–3409. 10.1099/mic.0.2008/019471-0 18957593

[B405] Touchon M. Cury J. Yoon E. J. Krizova L. Cerqueira G. C. Murphy C.. (2014). The genomic diversification of the whole *Acinetobacter* genus: origins, mechanisms, and consequences. Genome Biol. Evol. 6, 2866–2882. 10.1093/gbe/evu225 25313016 PMC4224351

[B406] Traglia G. M. Chua K. Centron D. Tolmasky M. E. Ramirez M. S. (2014). Whole-genome sequence analysis of the naturally competent *Acinetobacter baumannii* clinical isolate A118. Genome Biol. Evol. 6, 2235–2239. 10.1093/gbe/evu176 25164683 PMC4202317

[B407] Traglia G. M. Quinn B. Schramm S. T. Soler-Bistue A. Ramirez M. S. (2016). Serum Albumin and Ca^2+^ Are Natural Competence Inducers in the Human Pathogen *Acinetobacter baumannii*. Antimicrob. Agents Chemother. 60, 4920–4929. 10.1128/AAC.00529-16 27270286 PMC4958237

[B408] Trebosc V. Gartenmann S. Royet K. Manfredi P. Totzl M. Schellhorn B.. (2016). A novel genome-editing platform for drug-resistant *Acinetobacter baumannii* reveals an AdeR-unrelated tigecycline resistance mechanism. Antimicrob. Agents Chemother. 60, 7263–7271. 10.1128/AAC.01275-16 27671072 PMC5119006

[B409] Tsakris A. Ikonomidis A. Poulou A. Spanakis N. Vrizas D. Diomidous M.. (2008). Clusters of imipenem-resistant *Acinetobacter baumannii* clones producing different carbapenemases in an intensive care unit. Clin. Microbiol. Infect. 14, 588–594. 10.1111/j.1469-0691.2008.01996.x 18397334

[B410] Tsakris A. Ikonomidis A. Pournaras S. Tzouvelekis L. S. Sofianou D. Legakis N. J.. (2006). VIM-1 metallo-β-lactamase in *Acinetobacter baumannii*. Emerg. Infect. Dis. 12, 981–983. 10.3201/eid1206.051097 16707056 PMC3373053

[B411] Tsakris A. Ikonomidis A. Spanakis N. Pournaras S. Bethimouti K. (2007). Identification of a novel *bla*_OXA-51_ variant, *bla*_OXA-92_, from a clinical isolate of *Acinetobacter baumannii*. Clin. Microbiol. Infect. 13, 348–349. 10.1111/j.1469-0691.2006.01598.x 17391399

[B412] Tsuji B. T. Landersdorfer C. B. Lenhard J. R. Cheah S. E. Thamlikitkul V. Rao G. G.. (2016). Paradoxical effect of polymyxin B: high drug exposure amplifies resistance in *Acinetobacter baumannii*. Antimicrob. Agents Chemother. 60, 3913–3920. 10.1128/AAC.02831-15 27067330 PMC4914656

[B413] Turner P. J. Greenhalgh J. M. MYSTIC Study Group (Europe) (2003). The activity of meropenem and comparators against *Acinetobacter* strains isolated from European hospitals, 1997–2000. Clin. Microbiol. Infect. 9, 563–567. 10.1046/j.1469-0691.2003.00591.x 12848736

[B414] Turton J. F. Ward M. E. Woodford N. Kaufmann M. E. Pike R. Livermore D. M.. (2006a). The role of IS*Aba1* in expression of OXA carbapenemase genes in *Acinetobacter baumannii*. FEMS Microbiol. Lett. 258, 72–77. 10.1111/j.1574-6968.2006.00195.x 16630258

[B415] Turton J. F. Woodford N. Glover J. Yarde S. Kaufmann M. E. Pitt T. L. (2006b). Identification of *Acinetobacter baumannii* by detection of the *bla*_OXA-51*-like*_ carbapenemase gene intrinsic to this species. J. Clin Microbiol. 44, 2974–2976. 10.1128/JCM.01021-06 16891520 PMC1594603

[B416] Umland T. C. Schultz L. W. MacDonald U. Beanan J. M. Olson R. Russo T. A. (2012). *In vivo*-validated essential genes identified in *Acinetobacter baumannii* by using human ascites overlap poorly with essential genes detected on laboratory media. MBio. 3:e00113–12. 10.1128/mBio.00113-12 22911967 PMC3428692

[B417] Vahaboglu H. Budak F. Kasap M. Gacar G. Torol S. Karadenizli A.. (2006). High prevalence of OXA-51-type class D β-lactamases among ceftazidime-resistant clinical isolates of *Acinetobacter* spp.: co-existence with OXA-58 in multiple centres. J. Antimicrob. Chemother. 58, 537–542. 10.1093/jac/dkl273 16816400

[B418] Valenzuela J. K. Thomas L. Partridge S. R. van der Reijden T. Dijkshoorn L. Iredell J. (2007). Horizontal gene transfer in a polyclonal outbreak of carbapenem-resistant *Acinetobacter baumannii*. J. Clin. Microbiol. 45, 453–460. 10.1128/JCM.01971-06 17108068 PMC1829019

[B419] Vijayakumar S. Gopi R. Gunasekaran P. Bharathy M. Walia K. Anandan S.. (2016). Molecular characterization of invasive carbapenem-resistant *Acinetobacter baumannii* from a tertiary care hospital in south India. Infect. Dis. Ther. 5, 379–387. 10.1007/s40121-016-0125-y 27553951 PMC5019981

[B420] Vilacoba E. Almuzara M. Gulone L. Traglia G. M. Figueroa S. A. Sly G.. (2013). Emergence and spread of plasmid-borne *tet(B)*::IS*CR2* in minocycline-resistant *Acinetobacter baumannii* isolates. Antimicrob. Agents Chemother. 57, 651–654. 10.1128/AAC.01751-12 23147737 PMC3535920

[B421] Vila J. Navia M. Ruiz J. Casals C. (1997). Cloning and nucleotide sequence analysis of a gene encoding an OXA-derived β-lactamase in *Acinetobacter baumannii*. Antimicrob. Agents Chemother. 41, 2757–2759. 9420053 10.1128/aac.41.12.2757 PMC164203

[B422] Vila J. Ruiz J. Goni P. Marcos A. Jimenez de Anta T. (1995). Mutation in the *gyrA* gene of quinolone-resistant clinical isolates of *Acinetobacter baumannii*. Antimicrob. Agents Chemother. 39, 1201–1203. 7625818 10.1128/aac.39.5.1201 PMC162713

[B423] Vincent J. L. Rello J. Marshall J. Silva E. Anzueto A. Martin C. D.. (2009). International study of the prevalence and outcomes of infection in intensive care units. JAMA 302, 2323–2329. 10.1001/jama.2009.1754 19952319

[B424] Voulgari E. Politi L. Pitiriga V. Dendrinos J. Poulou A. Georgiadis G.. (2016). First report of an NDM-1 metallo-β-lactamase-producing *Acinetobacter baumannii* clinical isolate in Greece. Int. J. Antimicrob. Agents 48, 761–762. 10.1016/j.ijantimicag.2016.09.006 27773496

[B425] Wang H. Guo P. Sun H. Wang H. Yang Q. Chen M. (2007). Molecular epidemiology of clinical isolates of carbapenem-resistant *Acinetobacter* spp. from Chinese hospitals. Antimicrob. Agents Chemother. 51, 4022–4028. 10.1128/AAC.01259-06 PMC2151426 17846127

[B426] Wang N. Ozer E. A. Mandel M. J. Hauser A. R. (2014). Genome-wide identification of *Acinetobacter baumannii* genes necessary for persistence in the lung. MBio 5, e01163–14. 10.1128/mBio.01163-14 24895306 PMC4049102

[B427] Wareham D. W. Gordon N. C. Hornsey M. (2011). *In vitro* activity of teicoplanin combined with colistin versus multidrug-resistant strains of *Acinetobacter baumannii*. J. Antimicrob. Chemother. 66, 1047–1051. 10.1093/jac/dkr069 21393131

[B428] Weber B. S. Harding C. M. Feldman M. F. (2015a). Pathogenic Acinetobacter: from the Cell Surface to Infinity and Beyond. J. Bacteriol. 198, 880–887. 10.1128/JB.00906-15 26712938 PMC4772598

[B429] Weber B. S. Ly P. M. Irwin J. N. Pukatzki S. Feldman M. F. (2015b). A multidrug resistance plasmid contains the molecular switch for type VI secretion in *Acinetobacter baumannii*. Proc. Natl. Acad. Sci. U.S.A. 112, 9442–9447. 10.1073/pnas.1502966112 26170289 PMC4522760

[B430] Weber B. S. Miyata S. T. Iwashkiw J. A. Mortensen B. L. Skaar E. P. Pukatzki S.. (2013). Genomic and functional analysis of the type VI secretion system in *Acinetobacter*. PLoS ONE 8:e55142. 10.1371/journal.pone.0055142 23365692 PMC3554697

[B431] Wormser G. P. Keusch G. T. Heel R. C. (1982). Co-trimoxazole (trimethoprim-sulfamethoxazole): an updated review of its antibacterial activity and clinical efficacy. Drugs 24, 459–518. 6759092 10.2165/00003495-198224060-00002

[B432] Wright M. S. Haft D. H. Harkins D. M. Perez F. Hujer K. M. Bajaksouzian S.. (2014). New insights into dissemination and variation of the health care-associated pathogen *Acinetobacter baumannii* from genomic analysis. MBio 5, e00963–e00913. 10.1128/mBio.00963-13 24449752 PMC3903280

[B433] Wu X. Chavez J. D. Schweppe D. K. Zheng C. Weisbrod C. R. Eng J. K.. (2016). *In vivo* protein interaction network analysis reveals porin-localized antibiotic inactivation in *Acinetobacter baumannii* strain AB5075. Nat. Commun. 7:13414. 10.1038/ncomms13414 27834373 PMC5114622

[B434] Yamamoto M. Nagao M. Matsumura Y. Matsushima A. Ito Y. Takakura S.. (2011). Interspecies dissemination of a novel class 1 integron carrying *bla*_IMP−19_ among *Acinetobacter* species in Japan. J. Antimicrob. Chemother. 66, 2480–2483. 10.1093/jac/dkr336 21862476

[B435] Yang H. Wang M. Yu J. Wei H. (2015). Antibacterial activity of a novel peptide-modified lysin against *Acinetobacter baumannii* and *Pseudomonas aeruginosa*. Front. Microbiol. 6:1471. 10.3389/fmicb.2015.01471 26733995 PMC4686776

[B436] Yang Y. S. Lee Y. Tseng K. C. Huang W. C. Chuang M. F. Kuo S. C.. (2016). *In vivo* and *in vitro* efficacy of minocycline-based combination therapy for minocycline-resistant *Acinetobacter baumannii*. Antimicrob. Agents Chemother. 60, 4047–4054. 10.1128/AAC.02994-15 27114274 PMC4914616

[B437] Yokoyama Y. Matsumoto K. Ikawa K. Watanabe E. Morikawa N. Takeda Y. (2015). Population pharmacokinetic-pharmacodynamic target attainment analysis of sulbactam in patients with impaired renal function: dosing considerations for *Acinetobacter baumannii* infections. J. Infect. Chemother. 21, 284–289. 10.1016/j.jiac.2014.12.005 25638291

[B438] Yum J. H. Yi K. Lee H. Yong D. Lee K. Kim J. M.. (2002). Molecular characterization of metallo-β-lactamase-producing *Acinetobacter baumannii* and Acinetobacter genomospecies 3 from Korea: identification of two new integrons carrying the *bla*_VIM-2_ gene cassettes. J. Antimicrob. Chemother. 49, 837–840. 10.1093/jac/dkf043 12003980

[B439] Yu Y. S. Zhou H. Yang Q. Chen Y. G. Li L. J. (2007). Widespread occurrence of aminoglycoside resistance due to ArmA methylase in imipenem-resistant *Acinetobacter baumannii* isolates in China. J. Antimicrob. Chemother. 60, 454–455. 10.1093/jac/dkm208 17561497

[B440] Zavascki A. P. Goldani L. Z. Li J. Nation R. L. (2007). Polymyxin B for the treatment of multidrug-resistant pathogens: a critical review. J. Antimicrob. Chemother. 60, 1206–1215. 10.1093/jac/dkm357 17878146

[B441] Zhang X. Yang T. Cao J. Sun J. Dai W. Zhang L. (2016). Mucosal immunization with purified OmpA elicited protective immunity against infections caused by multidrug-resistant *Acinetobacter baumannii*. Microb. Pathog. 96, 20–25. 10.1016/j.micpath.2016.04.019 27133268

[B442] Zhu J. Wang C. Wu J. Jiang R. Mi Z. Huang Z. (2009). A novel aminoglycoside-modifying enzyme gene *aac(6*′*)-Ib* in a pandrug-resistant *Acinetobacter baumannii* strain. J. Hosp. Infect. 73, 184–185. 10.1016/j.jhin.2009.05.012 19703723

[B443] Zimbler D. L. Park T. M. Arivett B. A. Penwell W. F. Greer S. M. Woodruff T. M.. (2012). Stress response and virulence functions of the *Acinetobacter baumannii* NfuA Fe-S scaffold protein. J. Bacteriol. 194, 2884–2893. 10.1128/JB.00213-12 22467784 PMC3370640

[B444] Zoued A. Brunet Y. R. Durand E. Aschtgen M. S. Logger L. Douzi B.. (2014). Architecture and assembly of the Type VI secretion system. Biochim. Biophys. Acta 1843, 1664–1673. 10.1016/j.bbamcr.2014.03.018 24681160

